# Safety and immunogenicity of rVSVΔG-ZEBOV-GP Ebola vaccine in adults and children in Lambaréné, Gabon: A phase I randomised trial

**DOI:** 10.1371/journal.pmed.1002402

**Published:** 2017-10-06

**Authors:** Selidji T. Agnandji, José F. Fernandes, Emmanuel B. Bache, Régis M. Obiang Mba, Jessica S. Brosnahan, Lumeka Kabwende, Paul Pitzinger, Pieter Staarink, Marguerite Massinga-Loembe, Verena Krähling, Nadine Biedenkopf, Sarah Katharina Fehling, Thomas Strecker, David J. Clark, Henry M. Staines, Jay W. Hooper, Peter Silvera, Vasee Moorthy, Marie-Paule Kieny, Akim A. Adegnika, Martin P. Grobusch, Stephan Becker, Michael Ramharter, Benjamin Mordmüller, Bertrand Lell, VEBCON Consortium, Sanjeev Krishna, Peter G. Kremsner

**Affiliations:** 1 Centre de Recherches Médicales de Lambaréné, Lambaréné, Gabon; 2 Institut für Tropenmedizin, Universitätsklinikum Tübingen, Tübingen, Germany; 3 German Centre for Infection Research (DZIF) partner sites Universitätsklinikum Tübingen and Gießen-Marburg-Langen, Germany; 4 Bernhard Nocht Hospital for Tropical Diseases, Bernhard Nocht Institute for Tropical Medicine and University Medical Center Hamburg-Eppendorf, Hamburg, Germany; 5 Center of Tropical Medicine and Travel Medicine, Department of Infectious Diseases, Division of Internal Medicine, Academic Medical Center, Amsterdam, The Netherlands; 6 Institute for Virology, Philipps-Universität Marburg, Marburg, Germany; 7 Centre for Diagnostics and Antimicrobial Resistance, Institute for Infection & Immunity, St. George’s, University of London, London, United Kingdom; 8 US Army Medical Research Institute of Infectious Diseases, Fort Detrick, Maryland, United States of America; 9 World Health Organization, Geneva, Switzerland; 10 Department of Parasitology, Leiden University Medical Center, Leiden, The Netherlands; 11 St. George’s University Hospitals NHS Foundation Trust, London, United Kingdom; Mahidol-Oxford Tropical Medicine Research Unit, THAILAND

## Abstract

**Background:**

The rVSV**Δ**G-ZEBOV-GP vaccine prevented Ebola virus disease when used at 2 × 10^7^ plaque-forming units (PFU) in a trial in Guinea. This study provides further safety and immunogenicity data.

**Methods and findings:**

A randomised, open-label phase I trial in Lambaréné, Gabon, studied 5 single intramuscular vaccine doses of 3 × 10^3^, 3 × 10^4^, 3 × 10^5^, 3 × 10^6^, or 2 × 10^7^ PFU in 115 adults and a dose of 2 × 10^7^ PFU in 20 adolescents and 20 children. The primary objective was safety and tolerability 28 days post-injection. Immunogenicity, viraemia, and shedding post-vaccination were evaluated as secondary objectives. In adults, mild-to-moderate adverse events were frequent, but there were no serious or severe adverse events related to vaccination. Before vaccination, Zaire Ebola virus (ZEBOV)–glycoprotein (GP)–specific and ZEBOV antibodies were detected in 11% and 27% of adults, respectively. In adults, 74%–100% of individuals who received a dose 3 × 10^4^, 3 × 10^5^, 3 × 10^6^, or 2 × 10^7^ PFU had a ≥4.0-fold increase in geometric mean titres (GMTs) of ZEBOV-GP-specific antibodies at day 28, reaching GMTs of 489 (95% CI: 264–908), 556 (95% CI: 280–1,101), 1,245 (95% CI: 899–1,724), and 1,503 (95% CI: 931–2,426), respectively. Twenty-two percent of adults had a ≥4-fold increase of ZEBOV antibodies, with GMTs at day 28 of 1,015 (647–1,591), 1,887 (1,154–3,085), 1,445 (1,013–2,062), and 3,958 (2,249–6,967) for the same doses, respectively. These antibodies persisted up to day 180 for doses ≥3 × 10^5^ PFU. Adults with antibodies before vaccination had higher GMTs throughout. Neutralising antibodies were detected in more than 50% of participants at doses ≥3 × 10^5^ PFU. As in adults, no serious or severe adverse events related to vaccine occurred in adolescents or children. At day 2, vaccine RNA titres were higher for adolescents and children than adults. At day 7, 78% of adolescents and 35% of children had recombinant vesicular stomatitis virus RNA detectable in saliva. The vaccine induced high GMTs of ZEBOV-GP-specific antibodies at day 28 in adolescents, 1,428 (95% CI: 1,025–1,989), and children, 1,620 (95% CI: 806–3,259), and in both groups antibody titres increased up to day 180. The absence of a control group, lack of stratification for baseline antibody status, and imbalances in male/female ratio are the main limitations of this study.

**Conclusions:**

Our data confirm the acceptable safety and immunogenicity profile of the 2 × 10^7^ PFU dose in adults and support consideration of lower doses for paediatric populations and those who request boosting.

**Trial registration:**

Pan African Clinical Trials Registry PACTR201411000919191

## Abstract 2

Sanjeev Krishna and colleagues present a phase I randomised trial for safety and immunogenicity of an Ebola vaccine in both adults and children in Gabon.

## Author summary

**Why was this study done?:**

- The worst Ebola outbreak in history ended in 2016 after killing about 11,323 individuals and infecting 28,650 individuals worldwide.
- This public health emergency accelerated efforts to develop a vaccine as part of the strategy to contain the outbreak.
- Two vaccine candidates with preclinical safety and efficacy data obtained from non-human primates entered human trials.
- The one used in our study is the rVSV**Δ**G-ZEBOV-GP vaccine, containing a non-infectious portion of a gene from the Zaire Ebola virus introduced into a recombinant vesicular stomatitis virus (rVSV), which itself is unlikely to cause disease in humans.
- To generate data for deployment of the vaccine, several dose-ranging phase I trials were initiated across centres in the United States, Europe, and Africa.

**What did the researchers do and find?:**

- We allocated 115 adults aged 18–50 years to receive 1 of the 5 doses used in the trial. A single intramuscular dose ranging from 3 × 10^3^ to 2 × 10^7^ plaque-forming units (PFU) was given, and participants were followed up until 6 months post-injection for safety and immunogenicity.
- Preliminary results led to the selection of the 2 × 10^7^ PFU dose for further development.
- We also included 20 adolescents (13–17 years) and 20 children (6–12 years), who received the 2 × 10^7^ PFU dose and were followed-up in a similar way as the adults.
- No vaccine-related serious or severe adverse event was reported by any participant.
- A high proportion of our population—even though residing in an area with no history of Ebola outbreak—had pre-vaccination antibodies specific to the Zaire Ebola virus.
- In adults, antibodies persisted up to 6 months post-injection at doses of 3 × 10^5^ to 2 × 10^7^ PFU.
- In participants with baseline antibodies, a dose as low as 3 × 10^4^ PFU could induce high antibody titres up to day 56 post-injection.
- Higher vaccine replication, leading to shedding of the vaccine in saliva and urine, occurred in children and adolescents.

**What do these findings mean?:**

- Our results and other findings show that this vaccine is safe and immunogenic.
- Lower vaccine doses may be needed in paediatric populations as well as for boosting after primary vaccination or naturally acquired immunity.

## Introduction

The western African Ebola virus disease (EVD) public health emergency of international concern ended in June 2016 [1], after infecting approximately 28,650 individuals, of whom 11,323 died [2,3]. Global commitment led to landmarks in vaccine development against EVD, with 8 candidates out of 15 undergoing evaluation in phase I–III clinical trials worldwide by the end of 2015 [4–6]. A live-attenuated recombinant vaccine consisting of the vesicular stomatitis virus (VSV), strain Indiana, with the gene for the Kikwit-95 Zaire Ebola virus (ZEBOV) glycoprotein (GP) replacing the VSV glycoprotein (G) had given acceptable results in non-human primate challenge models and was selected for accelerated clinical development. In European and African populations, the VEBCON Consortium (VSV-EBola CONsortium) carried out parallel dose-escalation phase I trials of the recombinant VSV (rVSV)–ZEBOV candidate vaccine in Germany (NCT02283099), Kenya (NCT02296983), and Gabon (PACTR2014000089322) and a double-blind phase I/II randomised controlled trial in Switzerland (NCT02287480). Three further phase II/III trials were later launched in Guinea, Sierra Leone, and Liberia. Results from phase I trials in the US [7] and preclinical data supported selection of the 2 × 10^7^ plaque-forming units (PFU) dose as the most immunogenic for phase IIb/III trials in Guinea, Sierra Leone, and Liberia. A final analysis of the Guinea trial showed that a single dose of 2 × 10^7^ PFU given immediately after contact with an index case was 100% (95% CI: 70%–100%, *P =* 0.0045) efficacious in preventing EVD in individuals, and protected the population through a ring vaccination strategy 10 days or more post-vaccination [8].

Detailed dose-ranging studies (3 × 10^5^, 3 × 10^6^, 1 × 10^7^, 2 × 10^7^, and 5 × 10^7^ PFU) at the 4 VEBCON sites showed acceptable safety, dose-dependent reactogenicity [9], and high seroconversion rates among all participants on day 28 after vaccination [9,10].

In Gabon, 2 seroprevalence studies in epidemic and non-epidemic regions showed varying proportions of participants with pre-vaccination ZEBOV-specific IgG antibodies [11,12]. In Lambaréné, with no reported EVD outbreak, ZEBOV-GP-specific antibody responses after vaccination were similar at 2 tested doses (3 × 10^5^ and 3 × 10^6^ PFU) [9]. This finding contrasted with that in vaccinees in Geneva, where antibody titres at 3 × 10^5^ PFU were significantly lower than responses to higher vaccine doses, including 1 × 10^7^ PFU and 5 × 10^7^ PFU [10]. Additionally, irrespective of vaccine dose, delayed oligoarthritis and skin and mucous membrane lesions emerged as vaccine-related adverse events in a proportion of recipients more than 1 week after vaccination in Geneva [10]. These delayed complications were not observed in Gabon, despite the fact that the same vaccine batch and similar doses were used [9].

Because of these divergent site-specific observations, we need further assessments of the vaccine in Ebola virus endemic areas as well as in children. We present a comparison of safety and immunogenicity outcomes in participants vaccinated with (1) 2 × 10^7^ PFU, the dose used in the efficacy trial; (2) 2 previously reported doses, 3 × 10^6^ and 3 × 10^5^ PFU [9]; and (3) 2 lower doses, 3 × 10^4^ PFU and 3 × 10^3^ PFU, in African adults. Furthermore, we report, to our knowledge for the first time, on children and adolescents aged 6 to 17 years vaccinated with 2 × 10^7^ PFU.

## Methods

### Study design and participants

The trial protocol was approved by the Scientific Review Committee of Centre de Recherches Médicales de Lambaréné (CERMEL), the Institutional Ethics Committee of CERMEL, the National Ethics Committee of Gabon, the World Health Organization (WHO) Ethics Committee, and the Institutional Ethics Committee of the Universitätsklinikum Tübingen. The trial was registered with the Pan African Clinical Trials Registry (PACTR201411000919191).

The study was a randomised, open-label, dose-escalation phase I trial at CERMEL in Gabon. The trial was initially designed to escalate doses to 3 × 10^5^, 3 × 10^6^, and 2 × 10^7^ PFU in 60 adults. After successive protocol amendments, a total of 115 adults (18–50 years), 20 adolescents (13–17 years), and 20 children (6–12 years) were enrolled, between 17 November 2014 and 7 July 2015. Written informed consent was obtained from adults and parents/guardians of adolescents/children, and written assent from minors aged 11–17 years, prior to study-related procedures (details are in S3 Text).

Healthy consenting volunteers who were aged 6–50 years and resident in the study area—which had no history of an Ebola outbreak—and willing to minimise blood/body fluid exposure to their relatives for 5 days post-vaccination were included. Field workers used the door-to-door approach to invite individuals from the Lambaréné community to screen for the study. After screening, individuals with a history of severe local or systemic allergic reaction to vaccination, known allergy to constituents of the rVSV**Δ**G-ZEBOV-GP vaccine, or any acute or chronic clinically significant medical or psychiatric condition were excluded. All pregnant and lactating women were excluded. Volunteers who received a licensed vaccine within 14 days (or 30 days for a live vaccine), had a history of blood donation within 60 days prior to vaccination, were positive for HIV and/or hepatitis B or C virus infection, or had an immunocompromised member in the family were also excluded from the study.

### Randomisation and treatment allocation

Randomisation and allocation was performed by an independent investigator from 17 November 2014 until 13 April 2015 using a web-generated sequence. Randomisation in permuted blocks was performed in 2 stages. In the first, participants were assigned in a ratio of 1:1:1 to 3 × 10^5^, 3 × 10^6^, and 2 × 10^7^ PFU, and in the second stage, in a ratio of 1:1 to 3 × 10^3^ and 3 × 10^4^ PFU. On 9 December 2014, a temporary consortium-wide safety hold was placed on doses above 1 × 10^7^ PFU due to adverse events reported at the Swiss site with doses of 1 × 10^7^ and 5 × 10^7^ PFU. In Gabon, only 1 participant had been allocated to the 2 × 10^7^ PFU dose. In all, 20, 20, 1, 20, and 20 adults were randomised to a vaccine dose of 3 × 10^5^, 3 × 10^6^, 2 × 10^7^, 3 × 10^3^, and 3 × 10^4^ PFU, respectively. Preliminary data from the 20 participants vaccinated with 3 × 10^5^ PFU and the initial 19 vaccinated with 3 × 10^6^ PFU were previously reported [9].

An unblinded safety review of VEBCON Consortium trials by the data and safety monitoring board lifted the safety hold on 5 January 2015. After this and during the third stage of the study, 19 adults were vaccinated with 3 × 10^6^ PFU without randomisation. In addition, Merck Sharp & Dohme selected the 2 × 10^7^ PFU dose for further development, as being the most immunogenic dose with an acceptable safety profile (S. Gupta, oral presentation at the WHO Ebola Research and Development Summit, 11–12 May 2015, Geneva) [13,14]. A subsequent amendment included 20 adolescents and 20 children aged 13 to 17 years and 6 to 12 years, respectively, to be vaccinated with 2 × 10^7^ PFU. The National Ethics Committee of Gabon recommended that adults from this population should be vaccinated with the intended dose before administration to the paediatric cohorts, so an additional 15 adults were included in the study (S3 Text).

### Vaccine and vaccination procedures

The rVSV**Δ**G-ZEBOV-GP vaccine, developed by the Canadian National Laboratory under the patent number WO2004011488 A2 and licensed to BioProtection Systems (NewLink Genetics), was the unique intervention in this trial. The vaccine was subsequently sublicensed to Merck and was manufactured at IDT Biologika (Dessau-Rosslau, Germany). WHO supplied single-dose vials of 1 × 10^8^ PFU (lot no 0030513) to conduct the trial at CERMEL, from a donation of rVSV**Δ**G-ZEBOV-GP by the Canadian government to WHO. The dispensed vials were reconstituted in serial dilutions for vaccination. A single injection of 1 ml of the reconstituted vaccine for the required dose was administered intramuscularly into the deltoid muscle of volunteers at vaccination (S1 Fig).

### Safety assessments

The nature, frequency, and severity of adverse events constituted the primary safety endpoint of the trial. Local and systemic reactogenicity symptoms and signs (solicited adverse events) were recorded for 14 days post-injection. Unsolicited adverse events, including laboratory anomalies, were recorded up to 28 days post-injection. Detailed descriptions of all serious adverse events were recorded throughout the study follow-up visits, as a secondary safety endpoint.

Solicited adverse events (pain, swelling, redness) were obtained by direct examination of the injection site, or direct questioning when follow-up occurred by telephone. Arthralgia and arthritic symptoms were later added as a solicited adverse event upon the request of the data and safety monitoring board. Participants were asked specifically if they were experiencing these symptoms.

### rVSVΔG-ZEBOV-GP viraemia and shedding

Plasma, saliva, and urine samples (at screening and days 1, 2, and 7 post-vaccination) were processed and stored in Trizol LS at the study site, until rVSV**Δ**G-ZEBOV-GP viral load determinations were performed by reverse transcriptase quantitative PCR as a secondary outcome. The lower limit of detection for rVSV**Δ**G-ZEBOV-GP RNA was 30 copies/ml, and the lower level of quantification was 100 copies/ml [9].

### Immunological assessments

As a secondary objective, enzyme-linked immunosorbent assays (ELISAs) were performed on days 0, 28, 56, 84, and 180 after injection. ZEBOV-specific antibody assays were conducted at the Institute for Virology, Marburg. Antibodies were detected using an antibody capture ELISA based on inactivated Ebola Zaire Makona virus particles [15]. ELISA for ZEBOV-GP-specific antibodies was performed at the US Army Medical Research Institute of Infectious Diseases (USAMRIID) using the Kikwit-95 ZEBOV strain GP (standard operating procedure AP-03-35-00). Antibodies were reported as geometric mean titres (GMTs), or geometric mean concentrations, of arbitrary ELISA units (AEU) per millilitre with 95% confidence intervals, as indicated.

Neutralising antibodies (Nabs) were detected using either particles of Ebola virus (Zaire isolate Mayinga, AF086833), with the assays being performed in a BSL4 laboratory (Institute for Virology, Marburg), or VSV pseudovirions expressing the luciferase reporter gene complemented by GP from the Kikwit-95 ZEBOV strain, with assays being performed at USAMRIID.

All 4 assays were previously reported by our team [9,15] and other researchers working on this candidate vaccine in the US [7].

### Statistical analysis

WHO estimated that a sample size of 74–124 participants would be needed across the VEBCON Consortium sites to show a 2-fold change in ZEBOV-specific antibody titres between vaccine doses and proposed a target sample size of approximately 250 participants for all sites [10]. We described the frequency and intensity of adverse events using counts and percentages, means and standard deviations, or medians and interquartile ranges (IQRs), for skewed continuous variables. Chi-squared test or Fisher’s exact test was used to compare pairwise proportions. Seropositivity rates were defined as the percentage of participants having AEU above a cutoff per vaccine group. Seroconversion rates were defined as the percentage of converted participants in each group. McNemar’s test was used to compare the seropositivity between day 0 and other days. We used Fisher’s test to perform inter-group comparisons and to determine the association between the seroconversion rate and seropositivity rate at each time point. Antibody concentrations or units were normalised using log transformations, and responses are reported as GMTs with 95% confidence intervals or geometric mean of AEU per millilitre with 95% confidence intervals. Student’s test or Wilcoxon’s paired test was used to compare magnitudes of antibody induced between day 0 and other days. All statistical analyses were conducted in R statistical software version 3.1.2 [16], except for viraemias (copies/millilitre of plasma), which were analysed with a Kruskal–Wallis test combined with Dunn’s multiple comparison test using GraphPad Prism version 6.

## Results

**Fig 1:**
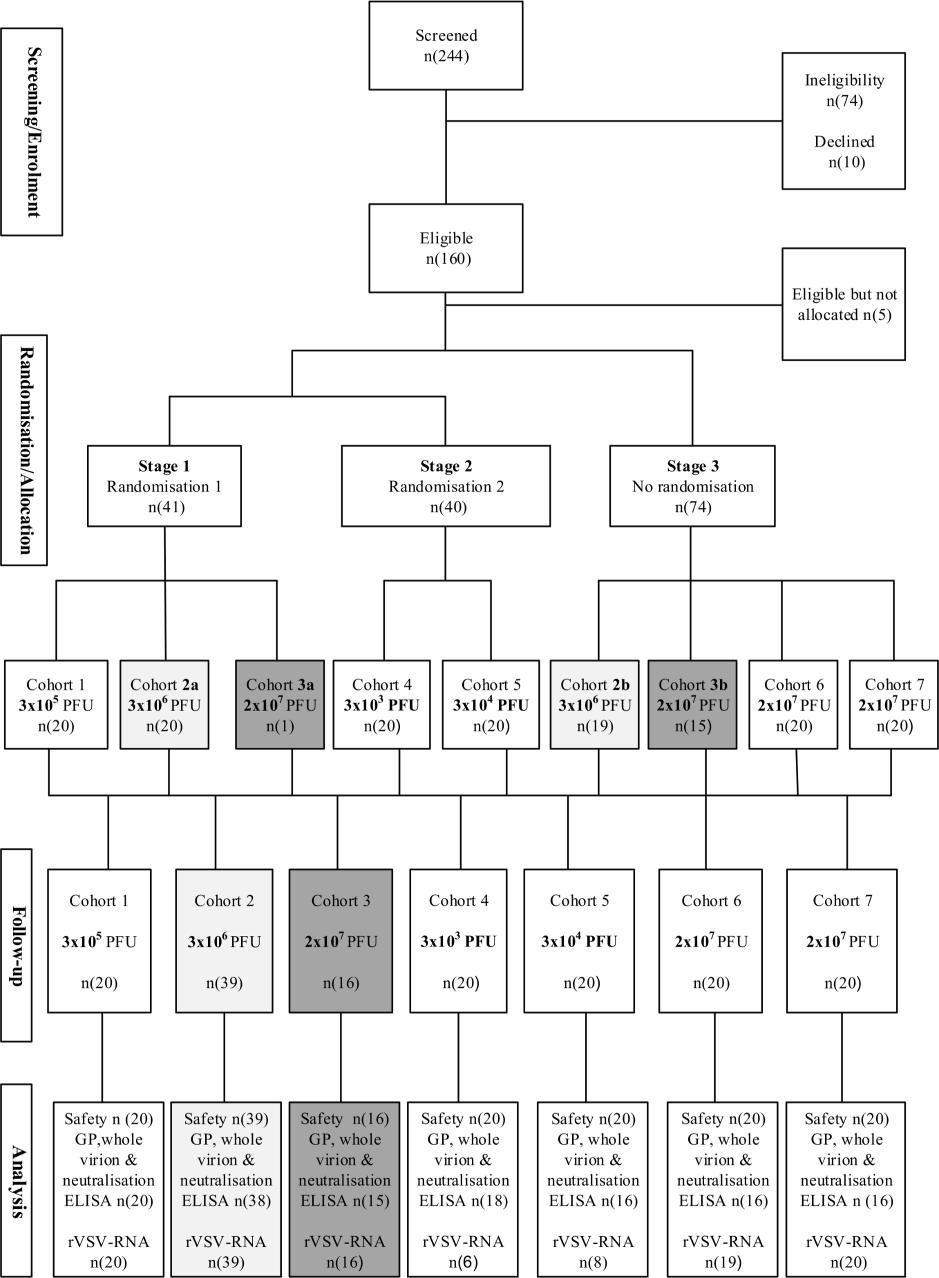
Participant flow diagram. Randomisation and flow of participants over a period of 6 months for adults (cohorts 1 to 5), adolescents (cohort 6; 13–17 years), and children (cohort 7; 6–12 years). Similar dose groups are matched with shading (light grey, 3 × 10^6^ PFU; dark grey, 2 × 10^7^ PFU). GP, glycoprotein; ELISA, enzyme-linked immunosorbent assay; PFU, plaque-forming units; rVSV, recombinant vesicular stomatitis virus.

From 21 November 2014 to 13 April 2015, 115 adults were vaccinated with a single injection of rVSV**Δ**G-ZEBOV-GP at 5 different doses. Twenty adolescents and 20 children were vaccinated between 8 May and 7 July 2015 (Fig 1).

Safety and immunogenicity data are reported until month 6 for adults, adolescents, and children for all 155 participants. In all, 108 (93%) adults and 36 (90%) adolescents and children attended all planned immunogenicity visits. Mean age and body mass index were similar among the 5 adult cohorts, with 21 adult women enrolled in the trial (Table 1).

**Table 1:** Baseline characteristics of study participants.

Characteristic	Adults (18–50 years)					Adolescents (13–17 years): 2 × 10^7^ PFU (*N =* 20)	Children (6–12 years): 2 × 10^7^ PFU (*N =* 20)
	3 × 10^3^ PFU (*N =* 20)	3 × 10^4^ PFU (*N =* 20)	3 × 10^5^ PFU*(*N =* 20)	3 × 10^6^ PFU*(*N =* 39)	2 × 10^7^ PFU (*N =* 16)		
**Sex, *n* (percent)**							
Male	13 (65)	17 (85)	14 (70)	35 (90)	15 (94)	19 (95)	16 (80)
Female	7 (35)	3 (15)	6 (30)	4 (10)	1 (6)	1 (5)	4 (2)
**Age (years), mean (SD)**	27 (8)	23 (5)	28 (7)	27 (7)	25 (6)	15 (1)	9 (1)
**BMI (kg/m**^**2**^**), mean (SD)**	23 (3)	23 (3)	23 (3)	23 (3)	23 (2)	18 (2)	16 (1)

*Already reported in The New England Journal of Medicine (doi: 10.1056/NEJMoa1502924) [9]: 3 × 10^5^ PFU, N = 20, and 3 × 10^6^ PFU, N = 19.

BMI, body mass index; PFU, plaque-forming units.

### Assessment of 5 vaccine doses in adult volunteers

#### Reactogenicity and tolerability

Headaches, fatigue, pain at injection site, gastrointestinal symptoms, and subjective fever were the most frequent symptoms. Of these, 68% were mild and 32% moderately intense, with similar frequencies up to day 28 across cohorts in adults. There were no vaccine-related severe adverse events (Tables 2 and S1).

**Table 2:** Reactogenicity to rVSVΔG-ZEBOV-GP vaccine until day 28 post-vaccination.

Adverse events/grading	Number (percent) of participants						
	Adults					Children: 2 × 10^7^ PFU (*N =* 20)	Adolescents: 2 × 10^7^ PFU (*N =* 20)
	3 × 10^3^ PFU (*N =* 20)	3 × 10^4^ PFU (*N =* 20)	3 × 10^5^ PFU* (*N =* 20)	3 × 10^6^ PFU* (*N =* 39)	2 × 10^7^ PFU (*N =* 16)		
***Adverse events (highest grade)***							
None	6^#^ (30%)	8 (40%)	2 (10%)	5 (13%)	1 (6%)	0 (0%)	0 (0%)
Mild	9^#^ (45%)	7 (35%)	11 (55%)	20 (51%)	10 (63%)	15 (75%)	14 (70%)
Moderate	5 (25%)	5 (25%)	7 (35%)	14 (36%)	5 (31%)	5 (25%)	6 (30%)
***Solicited injection site reactions***							
**Pain**							
None	17 (85%)	16 (80%)	17 (85%)	17 (44%)	7 (44%)	9 (45%)	10 (50%)
Mild	3 (15%)	3 (15%)	3 (15%)	22 (56%)	6 (37%)	9 (45%)	8 (40%)
Moderate	0 (0%)	1 (5%)	0 (0%)	0 (0%)	3 (19%)	2 (10%)	2 (10%)
**Swelling**							
None	20 (100%)	20 (100%)	20 (100%)	39 (100%)	15 (94%)	20 (100%)	19 (95%)
Mild	0 (0%)	0 (0%)	0 (0%)	0 (0%)	1 (6%)	0 (0%)	1 (5%)
***Solicited systemic reactions***							
**Headache**							
None	11 (55%)	15 (75%)	11 (55%)	17 (44%)	8 (50%)	10 (50%)	7 (35%)
Mild	6 (30%)	4 (20%)	6 (30%)	14 (36%)	6 (37%)	9 (45%)	10 (50%)
Moderate	3 (15%)	1 (5%)	3 (15%)	8 (20%)	2 (13%)	1 (5%)	3 (15%)
**Myalgia**							
None	17 (85%)	18 (90%)	18 (90%)	29 (74%)	14 (87%)	16 (80%)	14 (70%)
Mild	2 (10%)	1 (5%)	1 (5%)	6 (15%)	2 (13%)	3 (15%)	5 (25%)
Moderate	1 (5%)	1 (5%)	1 (5%)	4 (10%)	0 (0%)	1 (5%)	1 (5%)
**Subjective fever**							
None	18 (90%)	15 (75%)	19 (95%)	28 (72%)	9 (56%)	12 (60%)	12 (60%)
Mild	1 (5%)	3 (15%)	1 (5%)	9 (23%)	7 (44%)	7 (35%)	7 (35%)
Moderate	1 (5%)	2 (10%)	0 (0%)	2 (5%)	0 (0%)	1 (5%)	1 (5%)
**Fatigue**							
None	16 (80%)	14 (70%)	10 (50%)	20 (51%)	12 (75%)	10 (50%)	13 (65%)
Mild	3 (15%)	5 (25%)	7 (35%)	11 (28%)	3 (19%)	8 (40%)	3 (15%)
Moderate	1 (5%)	1 (5%)	3 (15%)	8 (20%)	1 (6%)	2 (10%)	4 (20%)
**Objective fever**							
None	17 (85%)	19 (95%)	19 (95%)	33 (85%)	12 (75%)	13 (65%)	15 (75%)
Mild	3 (15%)	1 (5%)	1 (5%)	3 (8%)	4 (25%)	7 (35%)	5 (25%)
Moderate	0 (0%)	0 (0%)	0 (0%)	3 (8%)	0 (0%)	0 (0%)	0 (0%)
**Gastrointestinal symptoms**							
None	17 (85%)	13 (65%)	10 (50%)	28 (72%)	10 (63%)	8 (40%)	14 (70%)
Mild	3 (15%)	6 (30%)	7 (35%)	10 (26%)	6 (38%)	10 (50%)	3 (15%)
Moderate	0 (0%)	1 (5%)	3 (15%)	1 (3%)	0 (0%)	2 (10%)	3 (15%)
**Chills**							
None	20 (100%)	20 (100%)	20 (100%)	35 (90%)	16 (100%)	15 (75%)	17 (85%)
Mild	0 (0%)	0 (0%)	0 (0%)	4 (10%)	0 (0%)	5 (25%)	3 (15%)
**Arthralgia**							
None	17 (85%)	17 (85%)	18 (90.0%)	26 (67%)	12 (75%)	17 (85%)	16 (80%)
Mild	0 (0%)	0 (0%)	0 (0%)	8 (21%)	1 (6%)	2 (10%)	3 (15%)
Moderate	3 (15%)	3 (15%)	2 (10%)	5 (13%)	3 (19%)	1 (5%)	1 (5%)
**Mouth ulcer**							
None	20 (100%)	20 (100%)	19 (95%)	37 (95%)	14 (88%)	18 (90%)	20 (100%)
Mild	0 (0%)	0 (0%)	1 (5%)	2 (5%)	1 (6%)	1 (5%)	0 (0%)
Moderate	0 (0%)	0 (0%)	0 (0%)	0 (0%)	1 (6%)	1 (5%)	0 (0%)
**Skin lesion**							
None	20 (100%)	19 (95%)	19 (95%)	38 (97%)	11 (69%)	19 (95%)	18 (90%)
Mild	0 (0%)	1 (5%)	1 (5%)	0 (0%)	4 (25%)	1 (5%)	2 (10%)
Moderate	0 (0%)	0 (0%)	0 (0%)	1 (3%)	1 (6%)	0 (0%)	0 (0%)
**Blister**							
None	20 (100%)	20 (100%)	20 (100%)	39 (100%)	15 (94%)	20 (100%)	20 (100%)
Mild	0 (0%)	0 (0%)	0 (0%)	0 (0%)	1 (6%)	0 (0%)	0 (0%)
***Unsolicited adverse events***							
**Malaria**							
None	19 (95%)	20 (100%)	15 (75%)	36 (92%)	8 (50%)	18 (90%)	17 (85%)
Mild	1 (5%)	0 (0%)	0 (0%)	0 (0%)	4 (25%)	0 (0%)	2 (10%)
Moderate	0 (0%)	0 (0%)	5 (25%)	3 (8%)	4 (25%)	2 (10%)	1 (5%)
**Rhinitis**							
None	18 (90%)	19 (95%)	18 (90%)	38 (97%)	12 (75%)	19 (95%)	17 (85%)
Mild	2 (10%)	1 (5%)	2 (10%)	1 (3%)	4 (25%)	1 (5%)	3 (15%)
**Cough**							
None	14 (70%)	17 (85%)	20 (100%)	39 (100%)	12 (75%)	17 (85%)	20 (100%)
Mild	3 (15%)	2 (10%)	0 (0%)	0 (0%)	4 (25%)	2 (10%)	0 (0%)
Moderat**e**	3 (15%)	1 (5%)	0 (0%)	0 (0%)	0 (0%)	1 (5%)	0 (0%)
**Other**							
None	14 (70%)	11 (55%)	3 (15%)	17 (44%)	0 (0%)	10 (50%)	14 (70%)
Mild	2 (10%)	4 (20%)	10 (50%)	13 (33%)	17 (68%)	8 (40%)	6 (30%)
Moderate	4 (20%)	5 (25%)	7 (35%)	9 (23%)	8 (32%)	2 (10%)	0 (0%)

*Already reported in The New England Journal of Medicine (doi: 10.1056/NEJMoa1502924) [9]: 3 × 10^5^ PFU, N = 20, and 3 × 10^6^ PFU, N = 19.

^#^Total number of participants reporting at least 1 event within 28 days after vaccination with rVSV**Δ**G-ZEBOV-GP vaccine. Only events with the highest grade are reported per cohort.

PFU, plaque-forming units.

Monocytes increased and lymphocytes decreased in the first week after vaccination in a dose-dependent fashion (S5 Table).

Mild-to-moderate symptoms reported at days 56, 84, and 180 and during unscheduled visits were considered unrelated to the study vaccine (S4 Table). Few haematological or biochemical changes of clinical significance were captured as adverse events, and these were followed up until resolution without sequelae.

A total of 11 adult participants experienced a serious adverse event. Six participants had malaria requiring hospitalisation, 2 underwent surgery due to appendicitis, and 1 was diagnosed with glaucoma. The last probably had the condition prior to enrolment after a detailed history was obtained, and is now receiving specialised care. Two individuals were hospitalised for bleeding after dental surgery and gastritis, respectively. All of these events were judged unrelated to the vaccine. Three women became pregnant after vaccination; they were monitored until delivery. Their neonates had no safety complications.

#### Immunogenicity

Eleven adults were excluded from either sampling and/or analysis of immunogenicity data: a male participant was HIV positive, 3 women became pregnant beyond day 28 after vaccination, and 7 participants received anti-tetanus vaccine/immunoglobulin. These participants were not sampled on subsequent visits (days 56, 84, and 180).

#### ZEBOV-GP-specific and ZEBOV antibodies

In all, 70%–100% of adult participants vaccinated with all doses ≥3 × 10^4^ PFU reached a greater than 4.0-fold increase of ZEBOV-GP-specific GMT at day 28. ZEBOV-GP antibody GMTs peaked at day 56, with antibody levels persistently higher than baseline up to 6 months post-vaccination (Table 3).

**Table 3:** Endpoint geometric mean titres, seropositivity rates, and proportions of seroresponders to rVSVΔG-ZEBOV-GP measured by ZEBOV-GP ELISA in adults.

Dose	Time*	*N*	GMT (95% CI)	Seropositivity (>200 AEU/ml), *N* (percent)	Seroresponse (≥4×), *N* (percent)	*P* value			
						Early change in GMT^†^	Change in seropositivity^‡^	Seropositivity and seroresponse^Ω^	Later change in GMT^β^
**3 × 10**^**3**^ **PFU**	D0	20	24 (9–60)	4 (20)	0 (0)	—	—	—	—
	D28	19	81 (35–184)	5 (26)	7 (37)	0.08	1	**0.03**	0.5
	D56	18	43 (14–131)	5 (28)	8 (44)	0.3	1	**0.006**	0.5
**3 × 10**^**4**^ **PFU**	D0	20	23 (9–63)	2 (10)	0 (0)	—	—	—	**—**
	D28	19	489 (264–908)	14 (74)	16 (84)	**<0.001**	**0.001**	**0.01**	**—**
	D56	16	633 (305–1,314)	14 (88)	15 (94)	**<0.001**	**0.001**	0.1	**—**
**3 × 10**^**5**^ **PFU**	D0	19	10 (4–22)	0 (0)	0 (0)	—	—	—	**—**
	D28	20	556 (280–1,101)	15 (75)	18 (90)	**<0.001**	**<0.001**	0.05	0.1
	D56	17	676 (246–1,859)	13 (77)	15 (88)	**<0.001**	**0.001**	**0.04**	**0.01**
	D84	17	536 (215–1,338)	14 (82)	15 (882)	**<0.001**	**<0.001**	**0.02**	**0.01**
	D180	16	365 (187–713)	12 (75)	15 (94)	**<0.001**	**0.002**	0.2	**—**
**3 × 10**^**6**^ **PFU**	D0	39	16 (9–27)	3 (8)	0 (0)	—	—	—	**—**
	D28	39	1,245 (899–1,724)	39 (100)	39 (100)	**<0.001**	**<0.001**	1	**<0.001**
	D56	37	1,331 (977–1,813)	36 (973)	37 (100)	**<0.001**	**<0.001**	1	**<0.001**
	D84	35	994 (731–1,352)	33 (943)	35 (100)	**<0.001**	**<0.001**	1	**<0.001**
	D180	37	685 (546–858)	35 (95)	36 (97)	**<0.001**	**<0.001**	0.05	**—**
**2 × 10**^**7**^ **PFU**	D0	16	47 (19–115)	4 (25)	0 (0)	—	—	—	**—**
	D28	16	1,503 (931–2,426)	16 (100)	16 (100)	**<0.001**	**0.001**	1	0.3
	D56	13	2,590 (1,604–4,182)	13 (100)	12 (92)	**<0.001**	**0.007**	1	**0.004**
	D84	14	1,826 (1,134–2,940)	14 (100)	13 (93)	**<0.001**	**0.004**	1	0.09
	D180	15	1,514 (997–2,301)	15 (100)	15 (100)	**<0.001**	**0.002**	1	**—**

ZEBOV-GP-specific antibodies are expressed in GMTs with 95% confidence intervals. Seropositivity is defined by geometric mean concentration > 200 AEU/ml. Seroresponse is defined by a ≥4-fold rise in GMT. P values < 0.05 are given in bold.

*Time point in day(s) since vaccination.

^†^Wilcoxon’s test for paired data. P < 0.05 indicates a statistical difference in antibody titre between day 0 and other days.

^‡^McNemar’s test. P < 0.05 indicates a statistical difference in seropositivity rate between day 0 and other days (28, 56, and 84 days post-vaccination).

^Ω^Fisher’s test. P < 0.05 indicates a statistical association between seropositivity and seroresponse for each time point.

^β^Wilcoxon’s test for paired data. P < 0.05 indicates a statistical difference in antibody titre between day 180 post-vaccination and days 28, 56, and 84 post-vaccination.

AEU, arbitrary enzyme-linked immunosorbent assay units; ELISA, enzyme-linked immunosorbent assay; GMT, geometric mean titre; PFU, plaque-forming units; ZEBOV, Zaire Ebola virus.

About 11% (13/114) of adult participants had ZEBOV-GP-specific ELISA antibody concentrations > 200 AEU/ml at baseline. The proportions of individuals with high concentrations at baseline were inconsistent across vaccine groups and ranged from 0% to 25%. Antibody concentrations were significantly higher at day 56 post-vaccination in individuals with prior antibodies following vaccination with doses of 3 × 10^3^, 3 × 10^4^, and 3 × 10^6^ PFU (Table 4).

**Table 4:** Endpoint geometric mean titres measured by USAMRIID ZEBOV-GP ELISA in adults with and without baseline specific antibodies.

Dose	Time *	With baseline GP-specific antibodies		Without baseline GP-specific antibodies		*P* value
		*N*	GMT (95% CI)	*N*	GMT (95% CI)	
**3 × 10**^**3**^ **PFU**	D0	4	346 (244–492)	16	12 (59–30)	**0.002**
	D28	4	305 (157–592)	15	57 (22–148)	0.08
	D56	4	295 (157–554)	14	25 (7–90)	**0.04**
**3 × 10**^**4**^ **PFU**	D0	2	549 (328–919)	18	16 (6–43)	**0.02**
	D28	2	3,489 (1,083–11,245)	17	388 (215–701)	**0.04**
	D56	2	5,229 (2,435–11,232)	14	468 (234–937)	**0.03**
**3 × 10**^**5**^ **PFU**	D0	—	—	19	10 (4–22)	—
	D28	—	—	20	556 (280–1,101)	—
	D56	—	—	17	676 (246–1,859)	—
	D84	—	—	17	536 (215–1,338)	—
	D180	—	—	16	365 (187–713)	—
**3 × 10**^**6**^ **PFU**	D0	3	310 (181–532)	36	12 (7–20)	**0.004**
	D28	3	6,307 (1,125–35,368)	36	1,088 (813–1,454)	**0.06**
	D56	3	4,263 (1,885–9,640)	34	1,201 (882–1,634)	**0.01**
	D84	3	2,984 (1,720–5,175)	32	897 (657–1,223)	**0.004**
	D180	3	1,616 (1,225–2,133)	34	635 (505–797)	**0.004**
**2 × 10**^**7**^ **PFU**	D0	4	375 (223–632)	12	23 (10–56)	**<0.001**
	D28	4	1,466 (761–2,824)	12	1,516 (820–2,801)	0.8
	D56	4	2,174 (1,713–2,760)	9	2,799 (1,401–5,590)	0.7
	D84	4	1,514 (882–2,597)	10	1,968 (1,036–3,738)	0.6
	D180	4	1,013 (672–1,526)	11	1,753 (1,028–2,990)	0.1

Seropositivity at day 0 (D0) defined by a GMT > 200 AEU/ml. P values < 0.05 are given in bold.

*Time point in day(s) since vaccination.

^†^Wilcoxon’s test. P < 0.05 indicates a statistical difference in antibody titre at each time point between the adults with and without the antibodies at D0.

AEU, arbitrary enzyme-linked immunosorbent assay units; ELISA, enzyme-linked immunosorbent assay; GMT, geometric mean titre; GP, glycoprotein; PFU, plaque-forming units; USAMRIID, US Army Medical Research Institute of Infectious Diseases; ZEBOV, Zaire Ebola virus.

The whole-virion assay is a less sensitive method to detect vaccine-induced antibody responses, which are directed against GP; 34% and 63% of vaccinees who received a dose equal to or more than 3 × 10^5^ PFU reached a greater than 2.0-fold increase in ZEBOV antibody GMT at day 28 and 56, respectively. Only 40% (30/74) of participants in the dose groups 3 × 10^5^, 3 × 10^6^, and 2 × 10^7^ PFU had a greater than 2.0-fold increase in antibody persisting up to 6 months post-injection (Table 5).

**Table 5:** Geometric mean titres, seropositivity rates, and proportions of seroresponders to rVSVΔG-ZEBOV-GP measured by whole-virion ELISA in adults.

Dose	Time point*	*N*	GMT (95% CI)	Seropositivity (>500 AEU/ml), *N* (percent)	Seroresponse, *N* (percent)		*P* value			
					≥2×	≥4×	Change in GMT^†^	Change in seropositivity^‡^	Seropositivity and seroresponse (≥2×)^Ω^	Seropositivity and seroresponse (≥4×)^β^
**3 × 10**^**3**^ **PFU**	D0	20	718 (529–975)	5 (25)	—	—	—		—	—
	D28	20	673 (975–896)	4 (20)	0 (0)	0 (0)	0.28	—	1	1
	D56	18	803 (565–1,141)	6 (33)	1 (6	0 (0)	0.40	—	0.33	1
**3 × 10**^**4**^ **PFU**	D0	20	949 (627–1,435)	7 (35)	—	—	—	—	—	—
	D28	20	1,015 (647–1,591)	7 (35)	1 (5)	0 (0)	0.20	—	0.35	1
	D56	16	1,029 (628–1,686)	5 (31)	1 (6)	0 (0)	0.40	—	0.31	0.31
**3 × 10**^**5**^ **PFU**	D0	19	575 (440–751)	1 (5)	—	—	—	—	—	—
	D7	20	641 (481–851)	3 (15)	2 (11)	0 (0)	0.42	0.47	**0.02**	1
	D14	18	674 (502–905)	4 (22)	2 (12)	1 (6)	0.58	0.24	**0.04**	**1**
	D28	20	1,887 (1,154–30,853)	13 (65)	11 (58)	10 (53)	**0.01**	**0.002**	**<0.001**	**<0.001**
	D56	17	1,402 (842–2,333)	8 (47)	7 (44)	7 (44	**0.02**	**0.02**	**<0.001**	**<0.001**
	D84	17	1,667 (1,098–2,531)	12 (71)	10 (63)	6 (38)	**0.03**	**0.004**	**0.001**	**0.09**
	D180	16	1,194 (809–1,762)	9 (56)	5 (33)	5 (33)	0.18	**0.02**	**0.02**	**0.02**
**3 × 10**^**6**^ **PFU**	D0	39	693 (565–850)	9 (23)	—	—	—	—	—	—
	D7	38	726 (573–920)	8 (21)	2 (5)	0 (0)	0.18	1	**0.04**	1
	D14	38	1,037 (734–1,463)	15 (40)	11 (29)	4 (11)	**0.003**	0.07	**<0.001**	**0.01**
	D28	39	1,445 (1,013–2,062)	21 (54)	17 (44)	11 (28)	**<0.001**	**0.001**	**<0.001**	**<0.001**
	D56	37	1,824 (1,316–2,527)	27 (73)	24 (65)	10 (27)	**<0.001**	**<0.001**	**<0.001**	**0.03**
	D84	36	1,586 (1,179–2,133)	25 (69)	19 (53)	8 (22)	**<0.001**	**<0.001**	**<0.001**	**0.07**
	D180	38	1,450 (1,105–1,903)	26 (68)	19 (50)	7 (18)	**<0.001**	**<0.001**	**<0.001**	**0.07**
**2 × 10**^**7**^ **PFU**	D0	16	1,625 (879–3,006)	9 (56)	—	—	—	—	—	—
	D7	16	1,220 (695–2,142)	7 (44)	0 (0)	0 (0)	**0.01**	0.47	1	1
	D14	16	2,153 (1,140–4,067)	10 (63)	2 (13)	1 (6)	0.16	1	0.5	1
	D28	16	3,958 (2,249–6,967)	13 (81)	7 (44)	4 (25)	**0.003**	0.22	0.21	0.5
	D56	13	4,402 (2,888–6,711)	12 (92)	6 (46)	4 (31)	**0.002**	0.22	1	1
	D84	14	3,638 (2,372–5,580)	13 (93)	7 (50)	3 (21)	**0.01**	0.13	1	1
	D180	15	2,963 (1,769–4,962)	13 (87)	6 (40)	3 (20)	0.3	0.22	0.48	1

ZEBOV antibodies are expressed in GMTs with 95% confidence intervals. Seropositivity is defined by a GMT > 500 AEU/ml. Seroresponse is expressed as a ≥2-fold or ≥4-fold increase in titre. P values < 0.05 are given in bold.

*Time point in day(s) since vaccination.

^†^Wilcoxon’s test for paired data. P < 0.05 indicates a statistically significant difference in antibody titre between day 0 and other days.

^‡^McNemar’s test. P < 0.05 indicates a statistically significant difference in seropositivity rate between day 0 and other days.

^Ω^Fisher’s test. P < 0.05 indicates a statistical association between seropositivity and seroresponse (≥2×) for each time point.

^β^Fisher’s test. P < 0.05 indicates a statistical association between seropositivity and seroresponse (≥4×) for each time point.

AEU, arbitrary enzyme-linked immunosorbent assay units; ELISA, enzyme-linked immunosorbent assay; GMT, geometric mean titre; PFU, plaque-forming units.

About 27% (31/115) of adults had ZEBOV antibody concentrations > 500 AEU/ml at baseline, with inconsistent frequencies (5% to 56%) across dose levels. In adults with pre-vaccination antibodies, a dose as low as 3 × 10^4^ PFU yielded a 2-fold increase in ZEBOV antibodies post-injection. Regardless of baseline status, the highest antibody titres were observed with the 2 × 10^7^ PFU dose (Table 6).

**Table 6:** Geometric mean titres of ZEBOV antibodies in adults by baseline antibody status measured by whole-virion ELISA.

Dose	Time*	With baseline ZEBOV antibodies			Without baseline ZEBOV antibodies		
		*N*	GMT (95% CI)	*P* value^†^	*N*	GMT (95% CI)	*P* value^†^
**3 × 10**^**3**^ **PFU**	D0	5	2,129 (1,276–3,552)	—	15	500 (—)	—
	D28	5	1,637 (780–3,435)	0.31	15	500 (—)	—
	D56	5	2,196 (1,064–4,532)	0.81	13	545 (466–638)	—
**3 × 10**^**4**^ **PFU**	D0	7	3,119 (2,106–4,621)	—	13	500 (—)	—
	D28	7	3,778 (2,642–5,403)	0.22	13	500 (—)	—
	D56	6	3,428 (1,618–7,260)	0.43	10	500 (—)	—
**3 × 10**^**5**^ **PFU**	D0	1	7,085 (—)	—	18	500 (—)	—
	D7	1	6,325 (—)	—	19	567 (479–672)	0.37
	D14	1	6,110 (—)	—	17	592 (495–708)	0.18
	D28	1	4,372 (—)	—	19	1,805 (1,084–3,007)	**0.003**
	D56	—	—	—	17	1,402 (831–2,364)	**0.02**
	D84	1	3,977 (—)	—	16	1,579 (1,027–2,427)	**0.006**
	D180	1	2,587 (—)	—	15	1,134 (758–1,695)	**0.02**
**3 × 10**^**6**^ **PFU**	D0	9	2,055 (1,458–2,896)	—	30	500 (—)	—
	D7	9	2,108 (1,184–3,753)	0.30	29	521 (481–565)	1
	D14	9	4,129 (1,986–8,585)	**0.04**	29	675 (534–854)	**0.02**
	D28	9	4,686 (2,905–7,559)	**0.01**	30	1,015 (714–1,445)	**0.002**
	D56	9	3,936 (2,009–7,711)	**0.02**	28	1,424 (1,021–1,987)	**<0.001**
	D84	9	3,371 (1,834–6,196)	**0.04**	27	1,234 (920–1,654)	**<0.001**
	D180	9	3,046 (1,758–5,276)	0.07	29	1,152 (882–1,504)	**<0.001**
**2 × 10**^**7**^ **PFU**	D0	9	4,065 (2,274–7,266)	—	7	500 (—)	—
	D7	9	2,440 (1,180–5,048)	**0.007**	7	500 (—)	—
	D14	9	4,422 (2,339–8,361)	0.42	7	854 (383–1,900)	0.37
	D28	9	5,633 (2,973–1,067)	**0.05**	7	2,515 (977–6,470)	0.06
	D56	8	4,365 (2,286–8,332)	0.07	5	4,463 (2,634–7,561)	0.06
	D84	8	4,179 (2,169–8,054)	0.31	6	3,024 (1,792–5,102)	**0.03**
	D180	9	3,691 (1,877–7,255)	0.50	6	2,131 (956–4,752)	0.06

Results are expressed in GMTs of AEU/millilitre with 95% confidence intervals. Seropositivity is defined by a GMT > 500 AEU/ml. Values below the threshold were given arbitrary units of 500 AEU/ml. P values < 0.05 are given in bold.

*Time point in day(s) since vaccination.

^†^Wilcoxon’s test for paired data used to compare antibody titres between time points; a P value < 0.05 indicates a statistically significant difference in antibody titre between day 0 and other days (D7, D14, D28, D56, D84, and D180). Samples from D84 for doses 3 × 10^3^ and 3 × 10^4^ PFU were not analysed.

AEU, arbitrary enzyme-linked immunosorbent assay units; ELISA, enzyme-linked immunosorbent assay; GMT, geometric mean titre; PFU, plaque-forming units; ZEBOV, Zaire Ebola virus.

#### Neutralising antibodies

Nabs for doses of 3 × 10^4^, 3 × 10^5^, 3 × 10^6^, and 2 × 10^7^ were detected in 52%, 55%, 82%, and 62% of recipients against VSV-based Ebola pseudovirions (pseudovirion neutralisation assay 50% [PsVNA50]), respectively, and in 70%, 84%, and 56% of recipients against ZEBOV virus particles. The highest Nab GMTs were observed in recipients of 2 × 10^7^ PFU. About 35%, 13%, and 25% of participants in the dose groups 3 × 10^5^, 3 × 10^6^, and 2 × 10^7^ PFU, respectively, had baseline Nabs against ZEBOV virus particles (defined as GMT > GMT + SD at D0). Higher Nab GMTs were observed at day 28 regardless of baseline antibody status (Tables 7, S8 and S9).

**Table 7:** Geometric mean titres, seropositivity rates, and proportions of seroresponders to rVSVΔG-ZEBOV-GP measured by ZEBOV PsVNA50 in adults.

Dose	Time point*	*N*	GMT (95% CI)	Seropositivity (>20 titre), *N* (percent)	Seroresponse (≥4×), *N* (percent)	*P* value		
						Change in GMT^†^	Change in concentration^‡^	Change in seropositivity^Ω^
**3 × 10**^**3**^ **PFU**	D0	19	19 (—)	0 (0)	0 (0)	—	—	**—**
	D28	19	24 (19–32)	3 (16)	2 (11)	0.1	0.2	**0.01**
**3 × 10**^**4**^ **PFU**	D0	19	19 (—)	0 (0)	0 (0)	—	—	**—**
	D28	19	70 (36–135)	10 (53)	8 (42)	**0.005**	**0.004**	**0.001**
**3 × 10**^**5**^ **PFU**	D0	19	19 (—)	0 (0)	0 (0)	—	—	**—**
	D28	20	66 (34–128)	11 (55)	7 (35)	**0.005**	**0.004**	**0.004**
	D180	16	21 (17–27)	1 (6)	1 (6)	1	1	0.06
**3 × 10**^**6**^ **PFU**	D0	39	19 (—)	0 (0)	0 (0)	—	—	**—**
	D28	39	81 (56–119)	32 (82)	18 (46)	**<0.001**	**<0.001**	**0.009**
	D180	37	20 (19–22)	6 (16)	0 (0)	**0.03**	**0.04**	1
**2 × 10**^**7**^ **PFU**	D0	16	19 (—)	0 (0)	0 (0)	—	—	**—**
	D28	16	126 (56–285)	10 (63)	10 (63)	**0.005**	**0.004**	**<0.001**
	D56	13	102 (52–202)	9 (69)	9 (69)	**0.009**	**0.007**	**0.001**
	D84	14	30 (23–41)	8 (57)	1 (7)	**0.01**	**0.01**	1
	D180	15	26 (21–34)	6 (40)	1 (7)	**0.03**	**0.04**	0.4

Results are expressed as geometric mean PsVNA50 neutralisation titres with 95 CIs. Seropositivity was defined as GMT > 20. Values below the threshold were given arbitrary titres of 19. Seroresponse was defined as a ≥4-fold increase. P values < 0.05 are given in bold.

*Time point in day(s) since vaccination.

^†^Wilcoxon’s test for paired data. P < 0.05 indicates a statistical difference in antibody titre between day 0 and other days.

^‡^McNemar’s test used to compare concentration between day 0 and other days. P value < 0.05 indicates a statistical difference in seropositivity rate between day 0 and other days.

^Ω^Fisher’s test used to compare seropositivity rate between day 0 and each time point. P value < 0.05 indicates a statistical difference between tested time points.

GMT, geometric mean titre; PFU, plaque-forming units; PsVNA50, pseudovirion neutralisation assay 50%; ZEBOV, Zaire Ebola virus.

### The vaccine dose of 2 × 10^7^ PFU in adult, adolescent, and child volunteers

#### Reactogenicity and tolerability

Twenty adolescents aged 13–17 years and 20 children aged 6–12 years were vaccinated with 1 intramuscular dose of 2 × 10^7^ PFU. Adolescents and children reported mostly headaches, fatigue, pain at injection site, gastrointestinal symptoms, and subjective fever. All reported symptoms were of mild (81% adolescents, 82% children) to moderate (19% adolescents, 18% children) intensity (Table 2).

As in adults, a general reduction in leukocyte counts was observed in adolescents and children within the first 2 days post-injection; leukocytes gradually restored to baseline values by day 28. An increase in monocyte and lymphocyte counts was observed between days 2 and 7, with lymphocytes rapidly restoring to baseline values by day 7 (S5 Table). No vaccine-related serious or severe adverse events occurred. One child was hospitalised for malaria.

#### rVSVΔG-ZEBOV-GP viraemia and shedding

Compared to adults vaccinated with 2 × 10^7^ PFU, rVSV**Δ**G-ZEBOV-GP RNA copy numbers in both adolescents and children were significantly higher at 1,592 (IQR 1,019–2,704) and 1,109 (IQR 663–1,963), respectively, versus 532 (IQR 373–898) in adults (*P =* 0.001) at day 2 post-injection (Table 8).

**Table 8:** Description of viraemia by dose and age.

Time point*	Adults										Children: 2 × 10^7^ PFU		Adolescents: 2 × 10^7^ PFU		*P* value^†^
	3 × 10^3^ PFU		3 × 10^4^ PFU		3 × 10^5^ PFU		3 × 10^6^ PFU		2 × 10^7^ PFU						
	*N*	Median copy number (IQR)	*N*	Median copy number (IQR)	*N*	Median copy number (IQR)	*N*	Median copy number (IQR)	*N*	Median copy number (IQR)	*N*	Median copy number (IQR)	*N*	Median copy number (IQR)	
D0	5	0 (0–0)	6	0 (0–0)	19	0 (0–0)	35	0 (0–0)	16	0 (0–0)	20	0 (0–0)	20	0 (0–0)	0.2
D1	6	0 (0–0)	8	0 (0–0)	18	3 (0–13)	33	228 (150–481)	16	334 (301–1,001)	4	731 (507–2,142)	19	655 (412–912)	0.5
D2	5	0 (0–0)	6	1 (0–12)	19	4 (0–30)	35	793 (401–1,286)	16	532 (373–898)	20	1,109 (663–1,963)	19	1,592 (1,019–2,704)	**0.001**
D7	5	0 (0–0)	6	4 (2–51)	12	1 (0–6)	32	7 (0–22)	16	4 (0–29)	19	0 (0–17)	17	0 (0–1)	0.1

All viraemia values expressed as median (IQR). P values < 0.05 are given in bold.

*Time point in day(s) since vaccination.

^†^Kruskal Wallis test. P < 0.05 indicates a significant statistical difference in viraemia values between the 3 groups (adults, children, and adolescents) at each time point at the 2 × 10^7^ PFU dose.

IQR, interquartile range; PFU, plaque-forming units.

**Fig 2:**
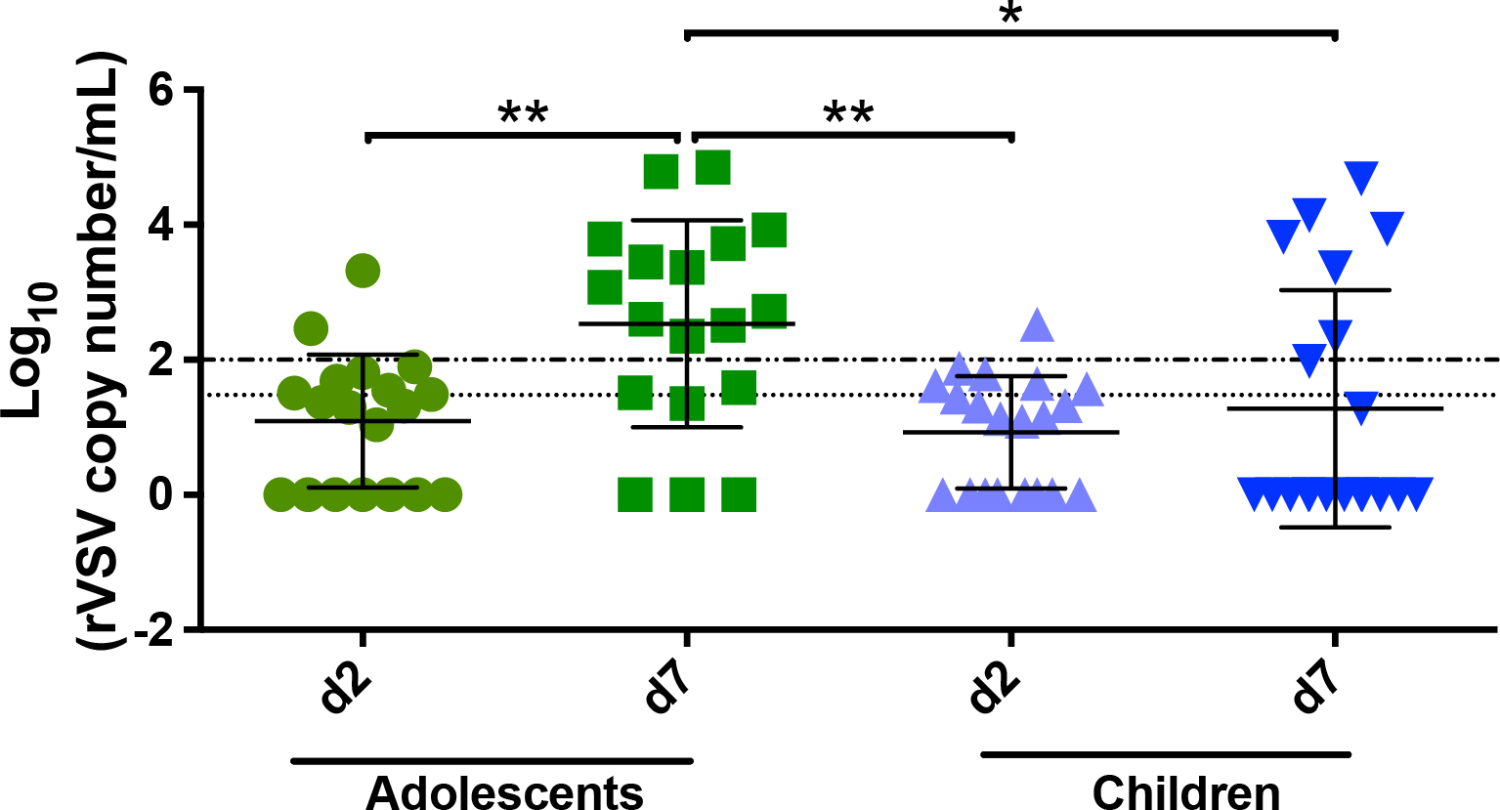
Viral load in saliva for children and adolescents. rVSV**Δ**G-ZEBOV-GP (rVSV) RNA copy numbers in saliva presented as log10 rVSV RNA copies/ml from day 2 and 7 (d2 and d7) post-injection in adolescents and children vaccinated with 2 × 10^7^ PFU. The broken line denotes the limit of quantitation, and the dotted line denotes the limit of detection. About 67% (12/18) and 30% (6/20) adolescents and children, respectively, had samples above the limit of quantification at day 7. *P < 0.05; **P < 0.01.PFU, plaque-forming units.

For viral shedding, there was a low percentage of positive samples at day 2, albeit below the level of quantification. At day 7, there was 1 child and 1 adolescent who had quantifiable RNA in urine. Saliva investigations showed that 42% and 30%, respectively, of adolescents and children had detectable RNA, corresponding with peak viraemia at day 2. At day 7, a considerably higher proportion of adolescents and children, 78% and 35% respectively, had RNA-positive saliva, with most samples being quantifiable (Figs 2 and S2; S13–S15 Tables).

### Immunogenicity

#### ZEBOV-GP-specific and ZEBOV antibodies

**Fig 3:**
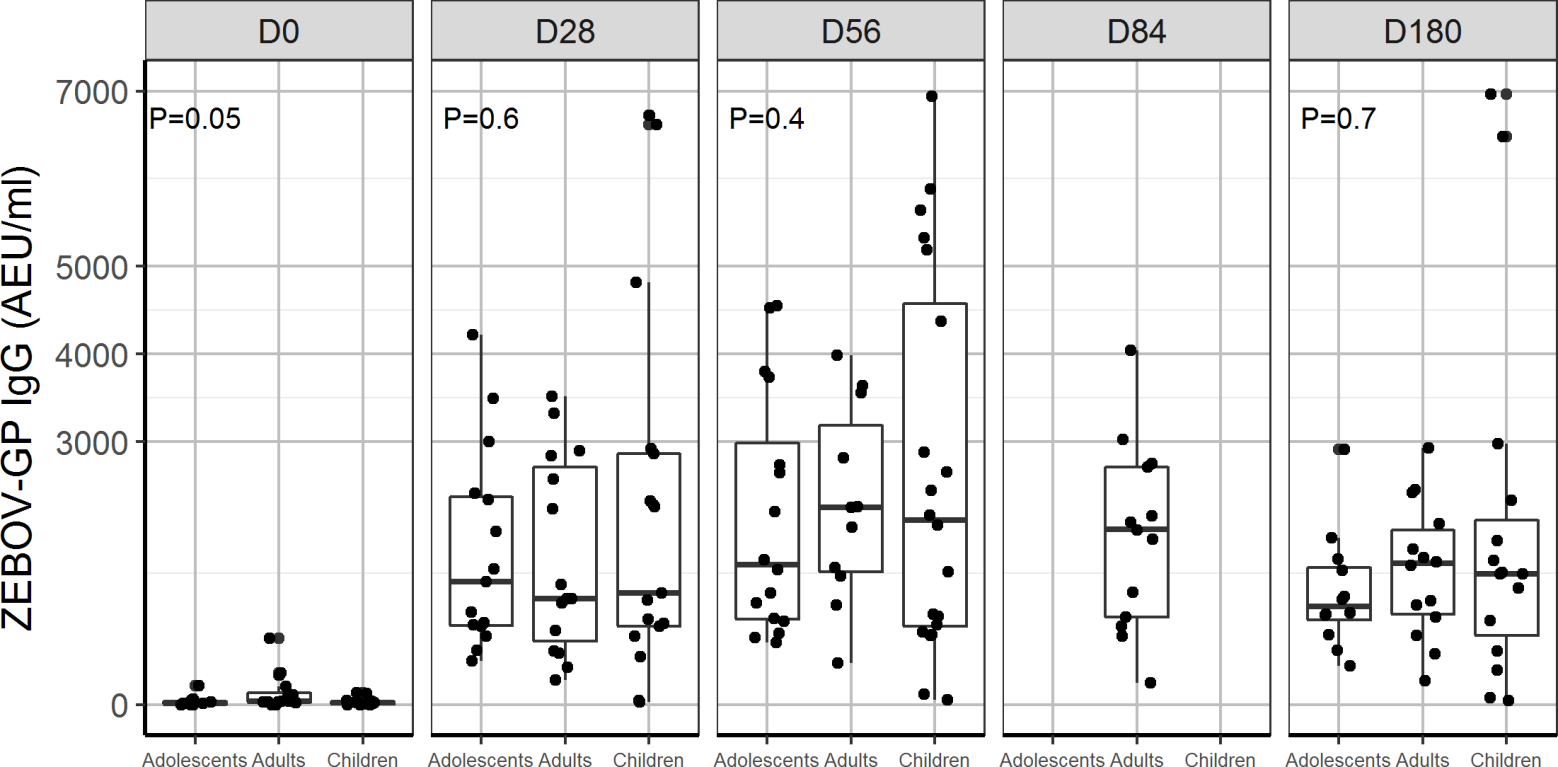
Glycoprotein antibody distribution by age group: Comparison of distribution of ZEBOV-GP IgG antibodies (AEU/ml) measured by USAMRIID ZEBOV-GP ELISA for dose 2 × 10^7^ PFU administered to children, adolescents, and adults from day 0, 28, 56, 84, and 180. Data were not available for children and adolescents at D84. P < 0.05 indicates a statistical difference in ZEBOV-GP IgG between children, adolescents, and adults. AEU, arbitrary enzyme-linked immunosorbent assay units; ELISA, enzyme-linked immunosorbent assay; GP, glycoprotein; PFU, plaque-forming units; USAMRIID, US Army Medical Research Institute of Infectious Diseases; ZEBOV, Zaire Ebola virus.

**Fig 4:**
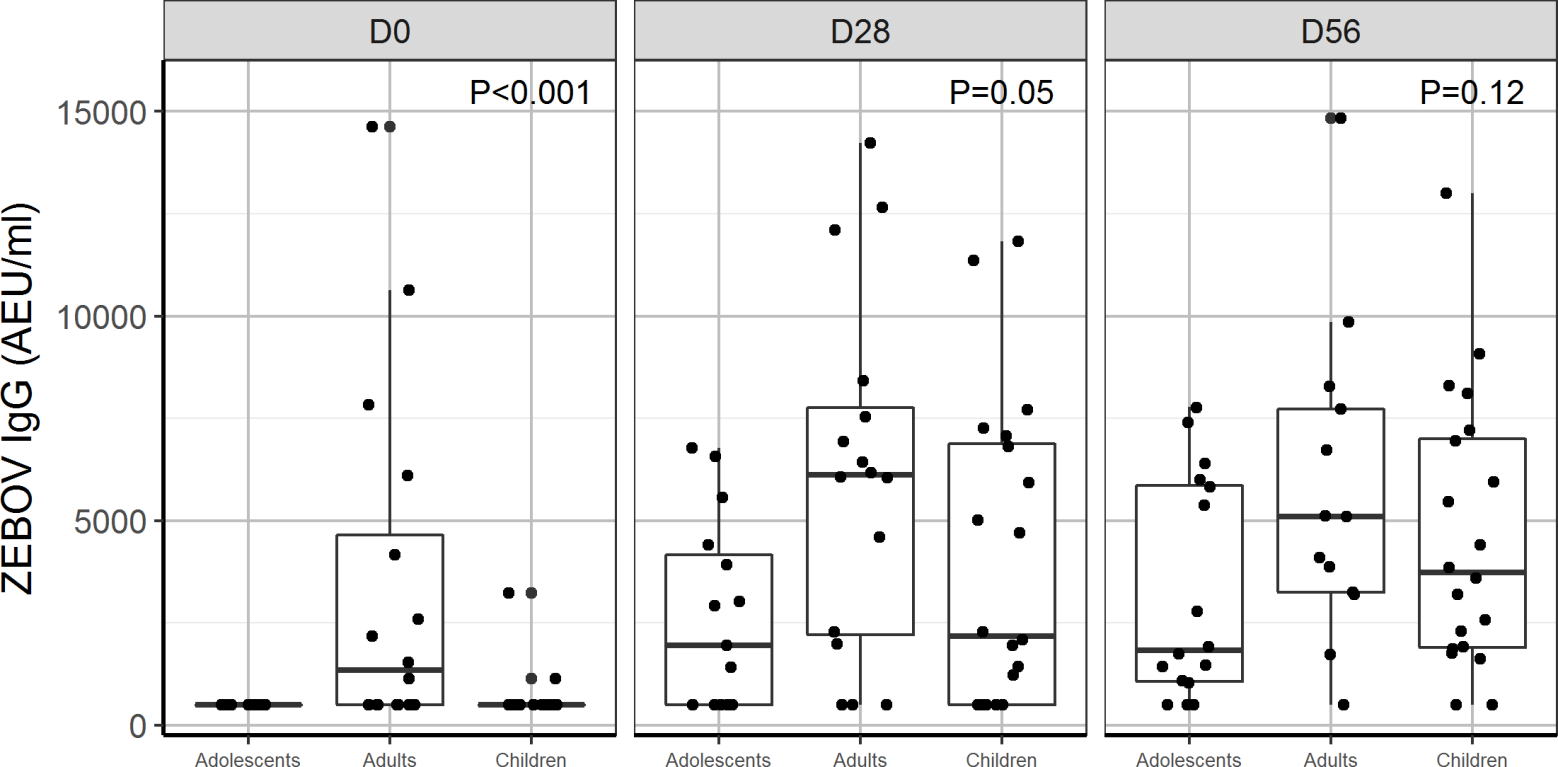
Antibody responses to whole-virion ELISA (AEU/ml) by age group: Comparison of geometric mean concentration of IgG antibodies for children, adolescents, and adults vaccinated with the 2 × 10^7^ PFU dose at day 0, 28, and 56. P < 0.05 indicates a statistical difference in antibody concentrations between age groups at the measured time points. AEU, arbitrary enzyme-linked immunosorbent assay units; ELISA, enzyme-linked immunosorbent assay; PFU, plaque-forming units; ZEBOV, Zaire Ebola virus.

In all, 90% and 100% of children and adolescents, respectively, receiving 2 × 10^7^ PFU had ZEBOV-GP-specific antibodies at day 28. Antibody titres were similar between adolescents, adults, and children using GP ELISA regardless of baseline antibody status (Figs 3, 4, S3 and S4).

By day 28, 70% and 60% of adolescents and children, respectively, were seropositive with whole-virion ELISA, compared to 81% of adults injected with 2 × 10^7^ PFU. Using a more sensitive GP ELISA, we obtained higher seropositivity rates, 100%, 100%, and 90% for adults, adolescents, and children, respectively, at day 28. We observed a ≥4-fold increase in ZEBOV-GP-specific antibody titres in about 90%–100% of adults, adolescents, and children consistently from day 28 to 180 post-injection. However, ZEBOV-GP-specific antibodies increased up to day 180 in children and adolescents (Table 9), while in adults, it peaked at day 56, and there was a decline until day 180 (Tables 3 and 5).

**Table 9:** Geometric mean titres, seropositivity rates, and proportions of seroresponders to rVSVΔG-ZEBOV-GP measured by ZEBOV-GP ELISA in children.

Cohort (2 × 10^7^ PFU)	Time point*	*N*	GMT (95% CI)	Seropositivity (>200 AEU/ml), *N* (percent)	Seroresponse (≥4×), *N* (percent)	*P* value		
						Change in GMT^†^	Change in seropositivity^‡^	Seropositivity and seroresponse^Ω^
**Children**	D0	15	15 (7–35)	0 (0)	0 (0)	—	—	**—**
	D28	20	1,620 (806–3,259)	18 (90)	19 (95)	**<0.001**	**<0.01**	**0.1**
	D56	20	1,599 (921–2,777)	18 (90)	20 (100)	**<0.001**	**<0.01**	1
	D180	20	2,069 (1,005–4,258)	18 (90)	18 (90)	**<0.001**	**<0.01**	**<0.01**
**Adolescents**	D0	15	12 (5–28)	1 (7)	0 (0)	—	—	**—**
	D28	15	1,427 (1,024–1,989)	15 (100)	15 (100)	**0.001**	**0.002**	1
	D56	16	1,744 (1,264–2,407)	16 (100)	16 (100)	**<0.001**	**<0.001**	1
	D180	17	2,541 (1,317–4,906)	17 (100)	17 (100)	**<0.001**	**<0.001**	1

Results are presented as GMTs with 95% confidence intervals. Seropositivity is defined by geometric mean concentration > 200 AEU/ml. Seroresponse is defined by a ≥4-fold rise in GMT. P values < 0.05 are given in bold.

*Time point in day(s) since vaccination.

^†^Wilcoxon’s test for paired data. P < 0.05 indicates a statistical difference in antibody titre between day 0 and other days.

^‡^McNemar’s test. P < 0.05 indicates a statistical difference in seropositivity rate between day 0 and other days.

^Ω^Fisher’s test. P < 0.05 indicates a statistical association between seropositivity and seroresponse for each time point.

AEU, arbitrary enzyme-linked immunosorbent assay units; ELISA, enzyme-linked immunosorbent assay; GMT, geometric mean titre; GP, glycoprotein; PFU, plaque-forming units; ZEBOV, Zaire Ebola virus.

Lower proportions of adults, adolescents, and children had a ≥4-fold increase in ZEBOV antibodies with whole-virion ELISA. Considering a ≥2-fold increase for this less sensitive ELISA, the yielded proportions were still much lower than those seen with GP ELISA. However, the proportion of adolescents and children with a ≥2- or ≥4-fold increase in ZEBOV antibodies increased from day 28 to 56, in contrast to the lack of difference between these time points in adults (Tables 5, 6 and S6). Thirteen percent of the children, but none of the adolescents, were seropositive for ZEBOV antibodies at baseline. None of the children and 7% of the adolescents were seropositive for ZEBOV-GP antibodies at baseline. As in adults, children with ZEBOV antibodies at baseline had higher GMTs at days 28 and 56 compared to those without baseline antibodies (Tables 6 and S7).

#### Neutralising antibodies

Against VSV pseudovirions, about 73% of children and adolescents elicited Nabs, with higher GMTs occurring at day 56 compared to day 28. In all, 95% and 80% of children and adolescents, respectively, had ZEBOV Nabs at day 28. Overall, children produced significantly higher GMTs of Nabs against ZEBOV particles (20 [95% CI: 13–32] compared to adolescents and adults, 10 [95% CI: 8–14] and 10 [95% CI: 6–14], respectively, *P =* 0.04) (S10–S12 Tables).

## Discussion

Although the 2014–2016 EVD emergency in western Africa has ended, the increasing mobility of people between remote and urban areas and the weak health systems in Ebolavirus endemic countries suggest that a future outbreak could reassert itself as a major international threat [17,18]. Risks include increased human-to-human secondary transmission as in the recent epidemic [19] as well as continuing transmission after recovery. Halting transmission by vaccination will be key in curbing future outbreaks [20]. The rVSV**Δ**G-ZEBOV-GP and ChAd3-ZEBOV vaccine candidates were selected by WHO in August 2014 for fast track clinical evaluation [6]. As part of these efforts, we examined a range of doses for rVSV**Δ**G-ZEBOV-GP in adults as well as safety and immunogenicity in children.

As reported earlier, rVSV**Δ**G-ZEBOV-GP doses of 3 × 10^5^ and 3 × 10^6^ PFU were well tolerated by 39 Lambaréné participants until day 28 and were safe up to 6 months [9]. Comparable to studies in Guinea [21] and US adults [7], transient cases of arthralgia were reported after vaccination [9,21,22], but no case of arthritis. In Kilifi, Kenya, there were 2 self-limiting, low-severity, and short-duration cases of arthritis [9,23]. This contrasts with a higher frequency of vaccine-induced arthritis (24%), dermatitis (9.8%), and vasculitis (2%) in Geneva [9,10,24] and more recently in Canada, the US, and Spain [25]. There may be similarities between rVSV**Δ**G-ZEBOV-GP vaccine and rubella vaccine, which also causes transient arthritides in some populations [26–28].

Ongoing studies are investigating the potential mechanisms by which rVSV**Δ**G-ZEBOV-GP vaccine might disseminate into peripheral tissues and induce arthritides in specific hosts. The magnitude of innate immune responses to rVSV**Δ**G-ZEBOV-GP vaccine correlated with the peak of rVSV RNA at day 1 in vaccinees of both Geneva and Lambaréné cohorts [29]. Importantly, high-dose vaccinees who experienced arthritis in Geneva had a significantly lower magnitude of early immune response compared to high-dose vaccinees who did not experience arthritis. These findings suggest that early and appropriate (in nature and magnitude) innate immune responses play a key role in limiting viral replication and dissemination to tissues and thus prevent the risk of arthritis. With lower vaccine dose (3 × 10^5^ PFU), the strength of early innate immune responses was similar in cases both with and without arthritis. Thus, rVSV-ZEBOV-induced arthritis may occur through mechanisms related to either vaccine dose or underlying factors that influence immune responses in vaccinees [29].

We observed higher and persistent viraemia in children and adolescents as well as shedding in saliva and urine, in contrast to the very low proportions or no shedding previously reported in the saliva of American and European adults vaccinated with 3 × 10^6^ to 5 × 10^7^ PFU [7,9,10]. The shedding in saliva did not correlate with oral symptoms. Although no alarming symptoms have been detected so far, our finding suggests that a vaccine dose of 2 × 10^7^ PFU exposed the paediatric population to prolonged or uncontrolled viraemia, with potential to disseminate to peripheral tissues. Specific studies are needed to elucidate the underlying mechanisms prolonging viraemia and causing shedding, such as differences in innate responses to vaccine between adults and younger participants. It is also necessary to assess any potential dissemination of rVSV-ZEBOV among household members of vaccinated children.

We observed dose-dependent antibody responses to the rVSV**Δ**G-ZEBOV-GP vaccine. A very low dose (≤3 × 10^3^ PFU) did not generate antibodies measured with either whole-virion or ZEBOV-GP-specific ELISA. In all individuals vaccinated with 3 × 10^4^, 3 × 10^5^, 3 × 10^6^, and 2 × 10^7^ PFU, the vaccine induced significant increases in ZEBOV-GP-specific antibodies measured by ZEBOV-GP ELISA alone for 3 × 10^4^ PFU and by both whole-virion and GP ELISAs for the other vaccine doses. The highest GMTs were observed with 2 × 10^7^ PFU irrespective of the ELISA method used.

As previously reported [9,11,12], our participants harboured naturally acquired antibodies against ZEBOV, or possibly related viruses. Western blot analysis of sub-samples showed that these antibodies were directed more often against nucleocapsid and matrix proteins of ZEBOV and not against GP. Nonetheless, 11% of our adults had ZEBOV-GP-specific antibodies before vaccination using the GP-specific ELISA. Individuals with baseline antibodies developed higher antibody titres with a dose as low as 3 × 10^4^ PFU compared to those without. The vaccine may have elicited higher titres of antibodies in the presence of natural GP-specific antibodies but also in the presence of antibodies directed against other viral components including nucleocapsid and matrix proteins (detected in baseline sera of some study participants) [9].

In adults, vaccine-induced antibodies peaked at day 56 and declined slowly by day 180. In children and adolescents, who showed high viraemia at day 2 and shed the vaccine until day 7, antibody titres increased until day 180. The kinetics of antibodies after vaccination may be affected by the specificity of pre-existing antibodies, and persistent vaccine replication may enhance immunogenicity. Also, the highest titres of Nabs against Ebola virus, which paralleled those against VSV pseudovirions, were generated at day 28 post-injection, regardless of baseline seropositivity. The relative roles of neutralising, GP, and non-GP antibodies in protection against EVD remain undefined, so it is difficult to draw conclusions on the clinical significance of correlations between GP-binding and neutralising antibodies produced after vaccinations.

The vaccine dose of 2 × 10^7^ PFU showed the optimal safety versus immunogenicity balance in our adult cohorts as well as in the Geneva and Hamburg cohorts [29,30]. These findings support the choice to use this dose in the context of outbreaks [8]. However, our data cannot explain the protection induced by the vaccine within 10 days observed in a phase III trial in Guinea [8] as the seroconversion rates and antibody titres were very weak before day 28 irrespective of the vaccine dose. Innate immune components induced immediately after vaccination may have played an important role in early protection. A recent study demonstrated the direct influence of innate immune responses on this vaccine’s safety and immunogenicity [29], a finding which supports the interest in assessing the efficacy of this vaccine beyond *Zaire ebolavirus* spp. as innate mechanisms can be cross-reactive.

Lower doses could be considered in vaccination strategies for children and individuals with impaired innate immune responses to control early rVSV replication. The dose of 3 × 10^5^ PFU generated significantly fewer rVSV RNA copies and shorter rVSV replication cycles but high antibody titres, so is of interest. As an incidental finding in our area, where Ebola virus transmission is endemic, a proportion of participants had antibodies directed against the whole-virus or GP-specific antigen before vaccination [11,12,31]. In those participants, a vaccine dose as low as 3 × 10^4^ PFU induced high antibody titres, suggesting lower vaccine doses should be considered in boosting strategies.

There are some limitations of our observations. For example, we cannot relate viral shedding in saliva with the oral symptoms reported by adolescents and children, suggesting that further studies are needed to evaluate this finding. We did not stratify participants based on antibody status at enrolment; future studies in Ebola virus endemic areas where such stratification is inherent in the design will provide insights into the relationships between naturally acquired antibodies and vaccine-induced immune responses and safety. We enrolled very few women across cohorts, leading to imbalances in the male/female ratio in our trial, which may reflect the general reluctance of women to enrol in phase I studies.

Our study confirms the acceptable safety and immunogenicity profile of the 2 × 10^7^ PFU dose in adults. However, considering the persistent replication of the rVSVΔGP-ZEBOV-GP vaccine in children and adolescents, further studies investigating lower doses in this population are warranted. In addition, lower vaccine doses should be considered when boosting individuals with pre-existing antibodies.

## Supporting information

S1 Fig Study vaccine reconstitution. (DOCX)

S2 Fig Viral load and viraemia in children, adolescents, and adults. (DOCX)

S3 Fig ZEBOV-GP-specific antibodies by age group in individuals without baseline antibodies. (DOCX)

S4 Fig ZEBOV antibodies measured by whole-virion ELISA by age group in individuals without baseline antibodies. (DOCX)

S1 Table Reactogenicity to rVSV-ZEBOV-GP vaccine until day 28. (DOCX)

S2 Table Reactogenicity to rVSV-ZEBOV-GP vaccine until day 28 in vaccinees with baseline ZEBOV-specific antibodies. (DOCX)

S3 Table Reactogenicity to rVSV-ZEBOV-GP vaccine until day 28 in vaccinees without baseline ZEBOV-specific antibodies. (DOCX)

S4 Table Frequency of symptoms after day 28 in adults. (DOCX)

S5 Table Haematology and biochemistry parameters. (DOCX)

S6 Table ZEBOV antibodies in geometric mean titres measured by whole-virion ELISA in children and adolescents. (DOCX)

S7 Table ZEBOV antibodies by baseline status in children measured by whole-virion ELISA. (DOCX)

S8 Table Neutralising antibodies to infectious ZEBOV isolate in adults. (DOCX)

S9 Table Neutralising antibodies to infectious ZEBOV isolate classified by baseline ZEBOV antibody status in adults. (DOCX)

S10 Table Neutralising antibodies to ZEBOV in children and adolescents. (DOCX)

S11 Table Neutralising antibodies to VSV peudovirions measured by PsVNA50 in children and adolescents. (DOCX)

S12 Table Comparison of antibodies in adults, children, and adolescents. (DOCX)

S13 Table rVSV RNA shedding and proportion of adolescents and children with detectable and quantifiable viral RNA. (DOCX)

S14 Table Viraemia in participants with baseline ZEBOV antibodies. (DOCX)

S15 Table Viraemia in participants without baseline ZEBOV antibodies. (DOCX)

S1 Text Trial protocol. (PDF)

S2 Text CONSORT checklist. (DOC)

S3 Text Dose escalation and randomisation. (DOCX)

S4 Text Data collection, management, and safety assessment. (DOCX)

## Abbreviations

AEU: arbitrary enzyme-linked immunosorbent assay units
CERMEL: Centre de Recherches Médicales de Lambaréné
ELISA: enzyme-linked immunosorbent assay
EVD: Ebola virus disease
GMT: geometric mean titre
GP: glycoprotein
IQR: interquartile range
Nab: neutralising antibody
PFU: plaque-forming units
PsVNA50: pseudovirion neutralisation assay 50%
rVSV: recombinant vesicular stomatitis virus
USAMRIID: US Army Medical Research Institute of Infectious Diseases
VSV: vesicular stomatitis virus
WHO: World Health Organization
ZEBOV: Zaire Ebola virus
