# Supplementary material for: Safety and immunogenicity of rVSVΔG-ZEBOV-GP Ebola vaccine in adults and children in Lambaréné, Gabon: A phase I randomised trial
Source: PLoS Med. 2017 Oct 6;14(10):e1002402. doi: 10.1371/journal.pmed.1002402 (PMC5630143; doi:10.1371/journal.pmed.1002402)
Supplement: S1 Fig — (DOCX) [file pmed.1002402.s001.docx]

**Figure S1: Study vaccine reconstitution**

This is an extract from the study pharmacy manual BioProtection Systems, Inc. (BPS).

The rVSV-ZEBOV-GP vaccine was supplied in vials containing 1ml of 1x10^8^ PFU/ml and was stored between -80°C+/-10 °C temperature until withdrawn for reconstitution. Vaccine reconstitution was performed by serial dilution. The 1 ml BPSC1001 vial was diluted with 9 ml of 0∙9% sodium chloride diluent to yield a concentration of 1x10^7^PFU. From the 1x10^7^ PFU constituted vial, 3∙5 ml was withdrawn and added to another vial containing 7∙5 ml of sodium chloride diluent to yield 11 ml of the 3x10^6^ PFU dose. From the 3x10^6^ PFU concentration, 1 ml was withdrawn and added to another vial containing 9 ml of sodium chloride to yield 10 ml of a concentration of 3x10^5^ PFU. From the 3x10^5^ PFU vial, 1 ml was obtained and added to 9 ml of sodium chloride in another vial to obtain 10 ml dose of 3x10^4^ PFU. From the concentration of 3x10^4^ PFU, 1 ml was obtained and added to 9 ml of sodium chloride to yield 10 ml of 3x10^3^ PFU.

To obtain the 2x10^7^ PFU dose, 4 ml of 0∙9% sodium chloride was added to 1 ml of the undiluted 1x10^8^ BPSC1001 vial to obtain 5 ml of constituted vaccine. From this diluted vaccine vial, 1 ml was drawn into a syringe and administered to a single participant.
