# Supplementary material for: Safety and immunogenicity of rVSVΔG-ZEBOV-GP Ebola vaccine in adults and children in Lambaréné, Gabon: A phase I randomised trial
Source: PLoS Med. 2017 Oct 6;14(10):e1002402. doi: 10.1371/journal.pmed.1002402 (PMC5630143; doi:10.1371/journal.pmed.1002402)

**Figure S2: Urine viral load and viraemia in children, adolescents and adults receiving the 2x10^7^ PFU dose**

B) **Viraemia in children**
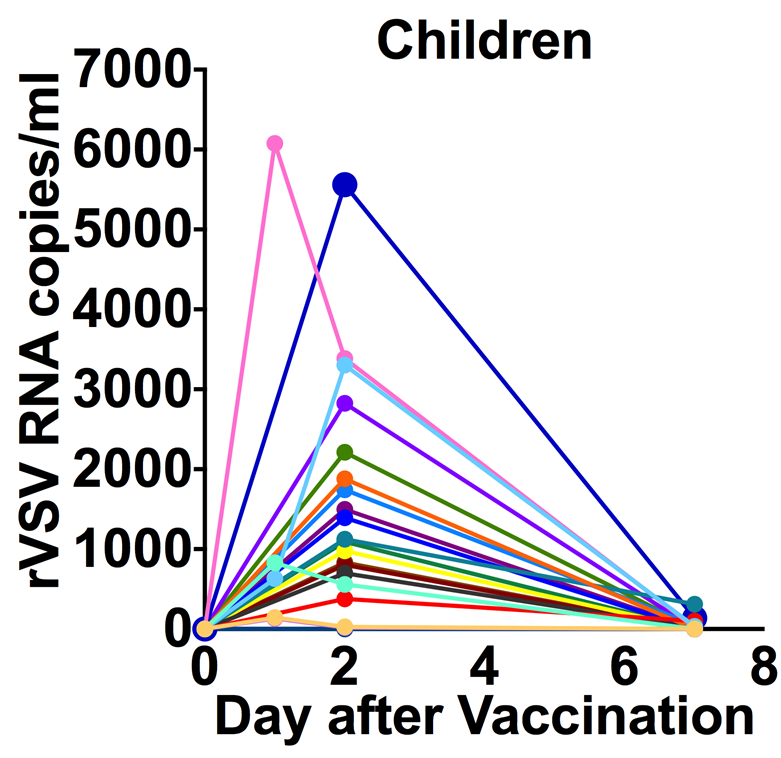


**A)** **Viral load in Urine for adolescents and Children**
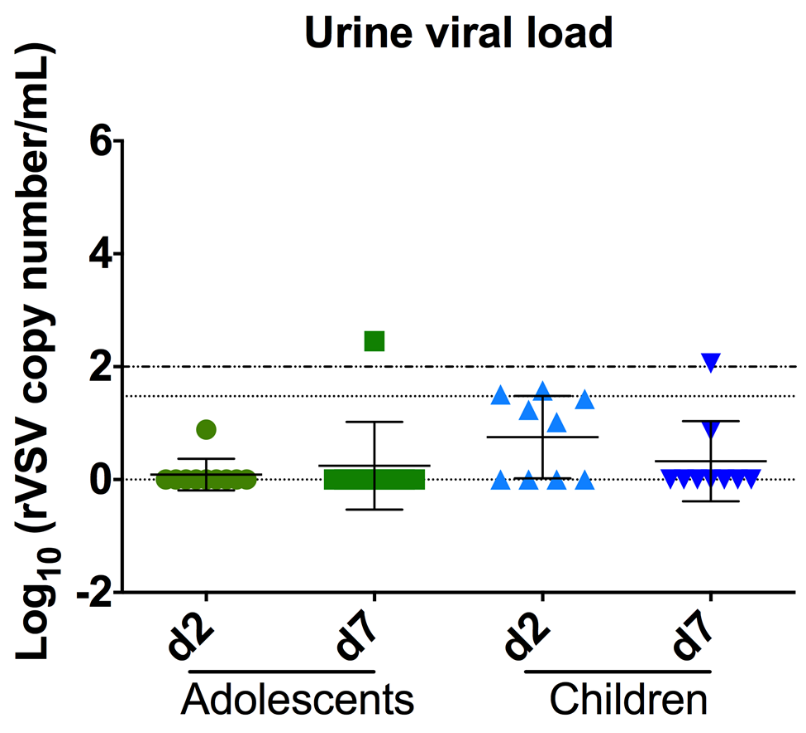


**Figure S2: Urine viral load and viraemia in children, adolescents and adults receiving the 2x10^7^ PFU dose.**

1. **Viral load in urine for children and adolescents. rVSV RNA copy numbers in saliva presented as log_10_ rVSV RNA copies/ml from day 2 and 7 post injection in adolescents and children vaccinated with 2x10^7^ PFU. The broken line denotes the limit of quantitation and the dotted line denotes the limit of detection.**
2. **Shows viral RNA copies/ml in plasma plotted for each child as an independent line over 7 days of assessment. Peak viraemia observed at day 2 post injection.**
3. **Shows viral RNA copies/ml in plasma plotted for each adolescent as an independent line over 7 days of assessment. Peak viraemia observed at day 2.**
4. **Shows viral RNA copies/ml in plasma plotted for each adult vaccinated with 2x10^7^ PFU as an independent line over 7 days of assessment**

**C) Viraemia in Adolescents**
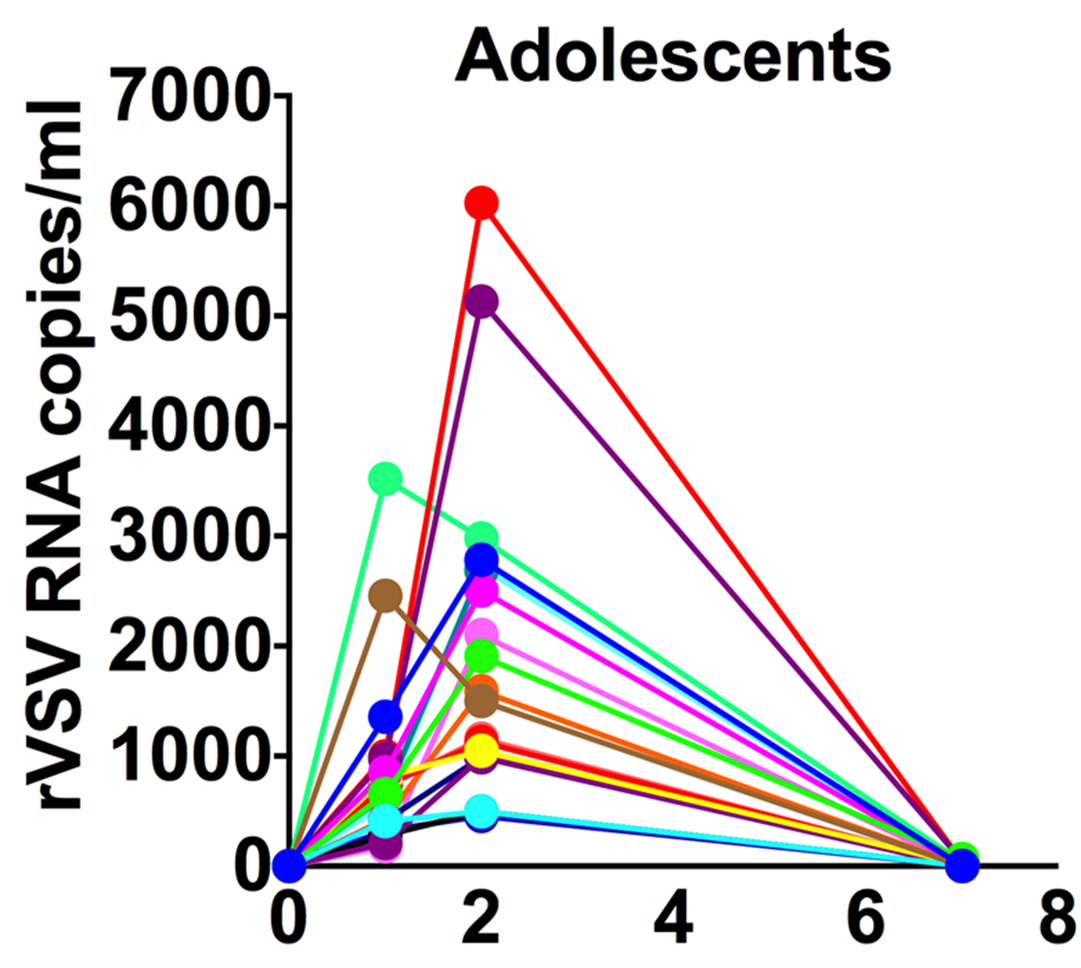


**D) Viraemia in Adults**


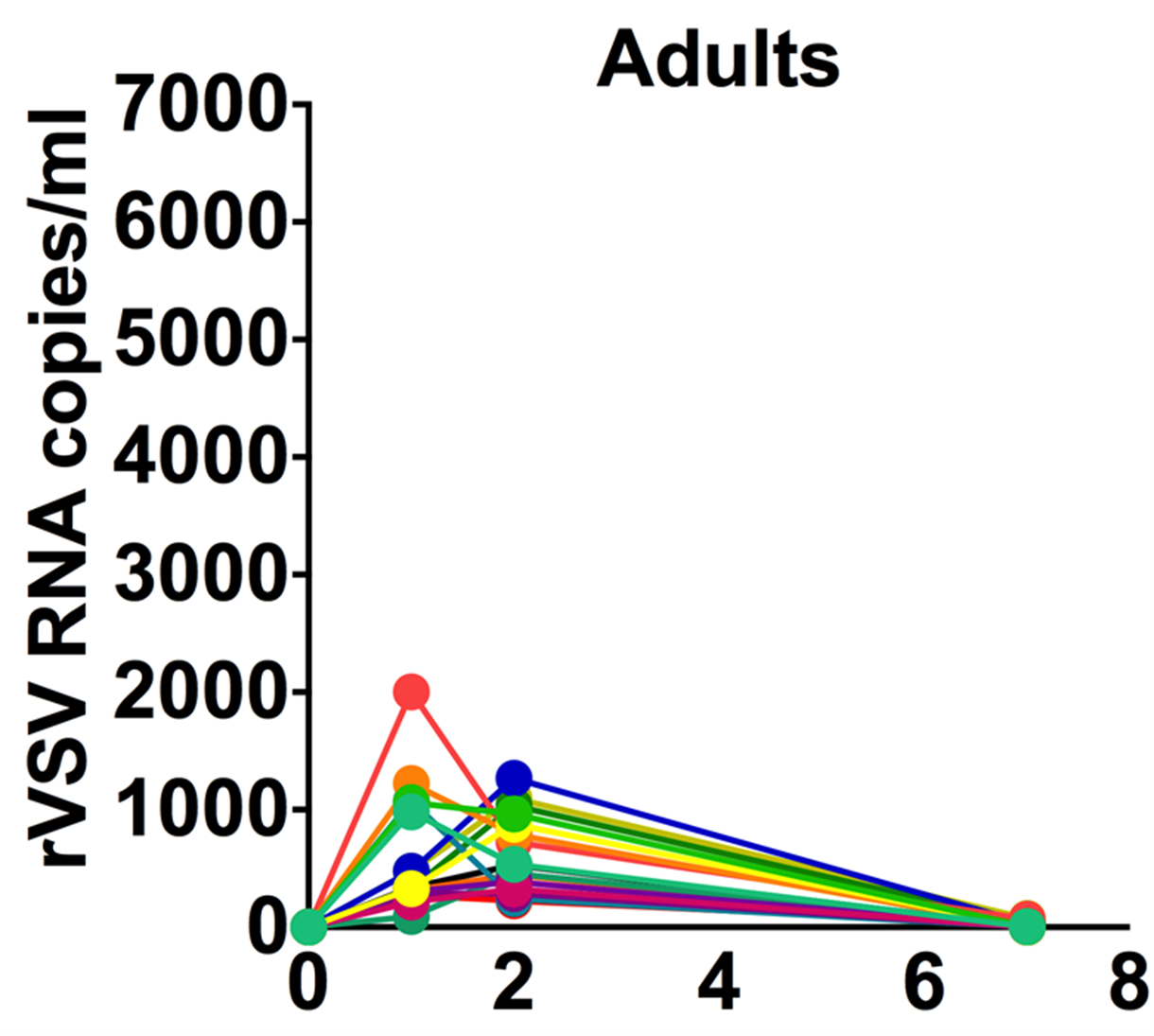

Supplement: S2 Fig — (DOCX) [file pmed.1002402.s002.docx]
