# Supplementary material for: Safety and immunogenicity of rVSVΔG-ZEBOV-GP Ebola vaccine in adults and children in Lambaréné, Gabon: A phase I randomised trial
Source: PLoS Med. 2017 Oct 6;14(10):e1002402. doi: 10.1371/journal.pmed.1002402 (PMC5630143; doi:10.1371/journal.pmed.1002402)
Supplement: S3 Fig — (DOCX) [file pmed.1002402.s003.docx]

# **S3 Fig. ZEBOV-GP-specific antibodies by age group in individuals without baseline antibodies**


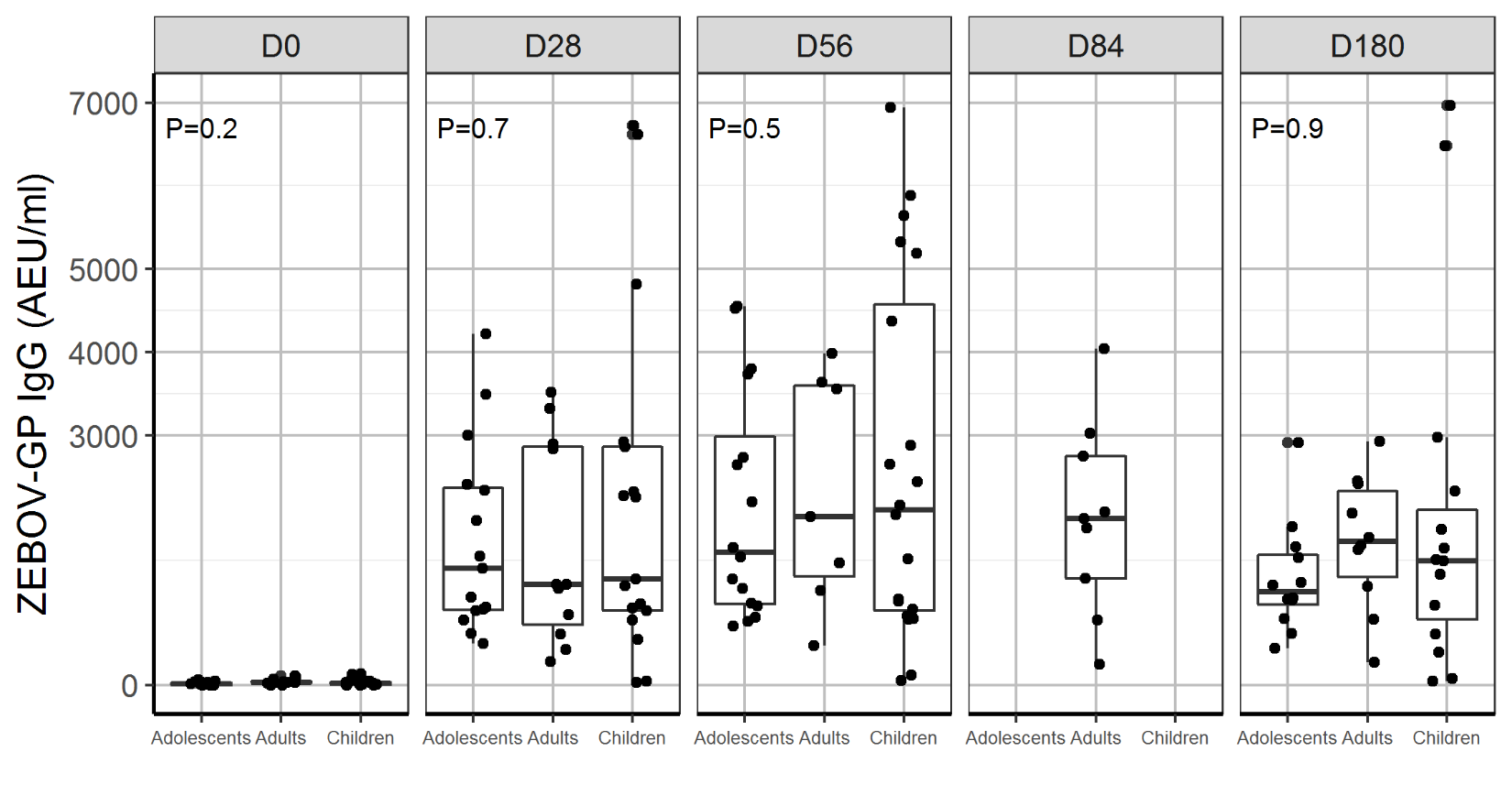


**S3 Fig: ZEBOV-GP-specific antibodies by age group in individuals without baseline antibodies: Comparison of distribution of ZEBOV-GP IgG antibodies (AEU/ml) measured by USAMRIID ZEBOV-GP ELISA for dose (2x10^7^ PFU) in children, adolescents and adults without baseline antibodies from day 0, 28, 56 and 180. Data were not available for children and adolescents at D84. P<0.05 indicates a statistical difference of ZEBOV-GP IgG between children, adolescents and adults.**
