# Supplementary material for: Safety and immunogenicity of rVSVΔG-ZEBOV-GP Ebola vaccine in adults and children in Lambaréné, Gabon: A phase I randomised trial
Source: PLoS Med. 2017 Oct 6;14(10):e1002402. doi: 10.1371/journal.pmed.1002402 (PMC5630143; doi:10.1371/journal.pmed.1002402)
Supplement: S4 Fig — (DOCX) [file pmed.1002402.s004.docx]

# **S4 Fig. ZEBOV antibodies measured by whole-virion ELISA by age group in individuals without baseline antibodies**


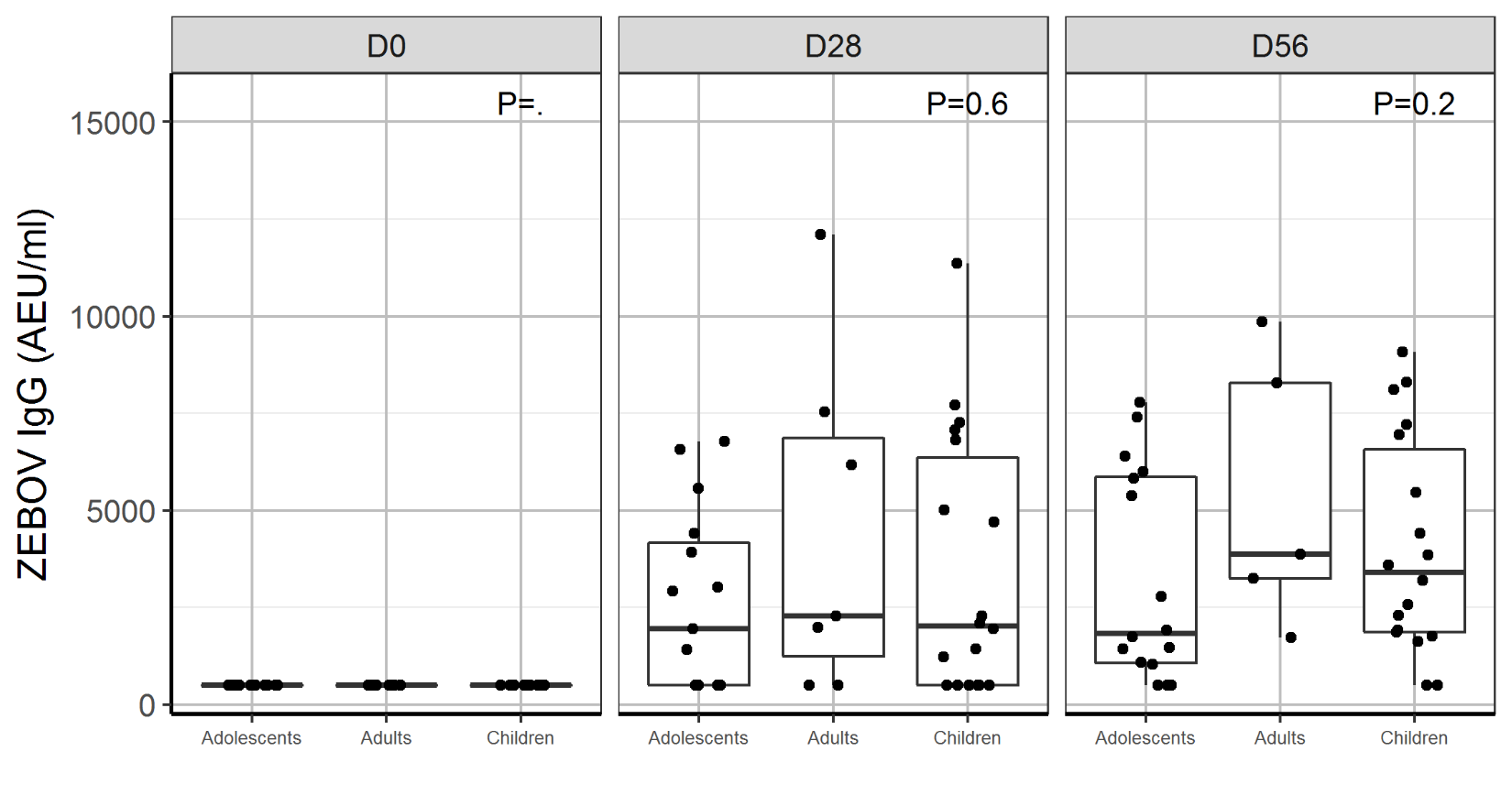


**S4 Fig. ZEBOV antibody distribution measured by whole-virion ELISA (AEU/ml) and presented per age group in individuals without baseline antibodies. Comparison of IgG antibodies in children, adolescents and adults without baseline antibodies vaccinated with 2x10^7^ PFU dose at day 0, 28 and 56. P<0.05 indicates a statistical difference in antibody concentrations between age groups at the measured time point.**
