# Supplementary material for: Safety and immunogenicity of rVSVΔG-ZEBOV-GP Ebola vaccine in adults and children in Lambaréné, Gabon: A phase I randomised trial
Source: PLoS Med. 2017 Oct 6;14(10):e1002402. doi: 10.1371/journal.pmed.1002402 (PMC5630143; doi:10.1371/journal.pmed.1002402)
Supplement: S1 Table — (DOCX) [file pmed.1002402.s005.docx]

# S1 Table. Reactogenicity to rVSV-ZEBOV vaccine until day 28.

|  | | **Adults** | | | | | | | | | | | | | | | | | | | | | | | | | | | | **Children** | | **Adolescent** | |
| --- | --- | --- | --- | --- | --- | --- | --- | --- | --- | --- | --- | --- | --- | --- | --- | --- | --- | --- | --- | --- | --- | --- | --- | --- | --- | --- | --- | --- | --- | --- | --- | --- | --- |
| **Adverse** | | **All adults** | | | | | **3x10^3^ PFU, n=20** | | | | | | | **3x10^4^ PFU, n=20** | | | | **3x10^5^ PFU*, n=20** | | | | | **3x10^6^ PFU*, n=39** | | | | **2x10^7^ PFU, n=16** | | | **2x10^7^ PFU, n=20** | | **2x10^7^ PFU,**  **n=20** | |
| **Events** | | **N** | **E (%)** | | | | **N** | | | | | | **E (%)** | **N** | | | **E (%)** | **N** | | | **E (%)** | | **N** | | **E (%)** | | **N** | | **E (%)** | **N** | **E (%)** | **N** | **E (%)** |
| **Any event** | |  |  | | | |  | | | | | |  |  | | |  |  | | |  | |  | |  | |  | |  |  |  |  |  |
| Mild | | 76 | 234 (68) | | | | 12 | | | | | | 33 (64) | 10 | | | 26 (67) | 19 | | | 54 (65) | | 28 | | 97 (70) | | 7 | | 24 (75) | 18 | 87 (82) | 20 | 79 (81) |
| Moderate | | 40 | 110 (32) | | | | 7 | | | | | | 19 (36) | 6 | | | 13 (33) | 11 | | | 29 (35) | | 13 | | 41 (30) | | 3 | | 8 (25) | 7 | 19 (18) | 6 | 19 (19) |
| **Solicited injection site reactions** | | | | | | | | | | | | | | | | | | | | | | | | | | | | | |  |  |  |  |
| **Pain** |  | |  | | | |  | | | |  | | |  | | |  |  | |  | | | |  |  |  | |  | |  |  |  |  |
| Mild | 29 | | 32 (91) | | | | 3 | | | | 4 (100) | | | 3 | | | 3 (75) | 3 | | 3 (100) | | | | 16 | 16 (100) | 4 | | 6 (75) | | 9 | 9 (75) | 8 | 8 (80) |
| Moderate | 3 | | 3 (9) | | | | 0 | | | | 0 (0) | | | 1 | | | 1 (25) | 0 | | 0 (0) | | | | 0 | 0 (0) | 2 | | 2 (25) | | 2 | 3 (25) | 2 | 2 (20) |
| **Swelling** |  | |  | | | |  | | | |  | | |  | | |  |  | |  | | | |  |  |  | |  | |  |  |  |  |
| Mild | 0 | | 0 (0) | | | | 0 | | | | 0 (0) | | | 0 | | | 0 (0) | 0 | | 0 (0) | | | | 0 | 0 (0) | 0 | | 0 (0) | | 0 | 0 (0) | 1 | 100 (0) |
| Moderate | 0 | | 0 (0) | | | | 0 | | | | 0 (0) | | | 0 | | | 0 (0) | 0 | | 0 (0) | | | | 0 | 0 (0) | 0 | | 0 (0) | |  | 0 (0) | 0 | 0 (0) |
| **Solicited systemic reactions** | | | | | | | | | | | | | | | | | | | | | | | | | | | | | | 0 |  |  |  |
| **Fatigue** |  | | |  | | | |  | | | |  | | |  | |  | |  | | |  | |  |  |  | |  | |  |  |  |  |
| Mild | 24 | | | 26 (72) | | | | 3 | | | | 3 (75) | | | 3 | | 3 (100) | | 7 | | | 7 (70) | | 9 | 11 (69) | 2 | | 2 (67) | | 9 | 10 (83) | 3 | 3 (43) |
| Moderate | 10 | | | 10 (28) | | | | 1 | | | | 1 (25) | | | 0 | | 0 (0) | | 3 | | | 3 (30) | | 5 | 5 (31) | 1 | | 1 (33) | | 2 | 2 (17) | 4 | 4 (57) |
| **Headache** |  | | |  | | | |  | | | |  | | |  | |  | |  | | |  | |  |  |  | |  | |  |  |  |  |
| Mild | 26 | | | 32 (68) | | | | 5 | | | | 6 (60) | | | 3 | | 5 (100) | | 6 | | | 7 (64) | | 9 | 10 (63) | 3 | | 4 (80) | | 8 | 9 (90) | 11 | 13 (81) |
| Moderate | 13 | | | 15 (32) | | | | 3 | | | | 4 (40) | | | 0 | | 0 (0) | | 3 | | | 4 (36) | | 6 | 6 (37) | 1 | | 1 (20) | | 1 | 1 (10) | 3 | 3 (19) |
| **Myalgia** |  | | |  | | | |  | | | |  | | |  | |  | |  | | |  | |  |  |  | |  | |  |  |  |  |
| Mild | 9 | | | 9 (64) | | | | 2 | | | | 2 (67) | | | 0 | | 0 (0) | | 1 | | | 1 (50) | | 6 | 6 (67) | 0 | | 0 (0) | | 3 | 3 (75) | 5 | 5 (83) |
| Moderate | 5 | | | 5 (36) | | | | 1 | | | | 1 (33) | | | 0 | | 0 (0) | | 1 | | | 1 (50) | | 3 | 3 (33) | 0 | | 0 (0) | | 1 | 1 (25) | 1 | 1 (17) |
| **Subjective fever** | | | | | | | | | | | | | | | | | | | | | | | | | | | | | |  |  |  |  |
| Mild | 16 | | | 18 (78) | | | | 1 | | | | 1 (50) | | | 2 | | 2 (50) | | 1 | | | 2 (100) | | 9 | 10 (83) | 3 | | 3 (100) | | 7 | 9 (90) | 7 | 9 (90) |
| Moderate | 5 | | | 5 (22) | | | | 1 | | | | 1 (50) | | | 2 | | 2 (50) | | 0 | | | 0 (0) | | 2 | 2 (17) | 0 | | 0 (0) | | 1 | 1 (10) | 1 | 1 (10) |
| **Objective fever** | | | | | | | | | | | | | | | | | | | | | | | | | | | |  | |  |  |  |  |
| Mild | 10 | | | | 11 (85) | | | | 3 | | | | 4 (100) | | | 1 | 1 (100) | | 1 | | | 1 (100) | | 3 | 3 (60) | 2 | | 2 (100) | | 7 | 7 (100) | 5 | 5 (100) |
| Moderate | 1 | | | | 2 (15) | | | | 0 | | | | 0 (0) | | | 0 | 0 (0) | | 0 | | | 0 (0) | | 2 | 2 (40) | 0 | | 0 (0) | | 0 | 0 (0) | 0 | 0 (0) |
| **Chill**s |  | | | |  | | | |  | | | |  | | |  |  | |  | | |  | |  |  |  | |  | |  |  |  |  |
| Mild | 3 | | | | 3 (100) | | | | 0 | | | | 0 (0) | | | 0 | 0 (0) | | 0 | | | 0 (0) | | 3 | 3 (100) | 0 | | 0 (0) | | 5 | 5 (100) | 3 | 3 (100) |
| Moderate | 0 | | | | 0 (0) | | | | 0 | | | | 0 (0) | | | 0 | 0 (0) | | 0 | | | 0 (0) | | 0 | 0 (0) | 0 | | 0 (0) | | 0 | 0 (0) | 0 | 0 (0) |
| **Arthralgia** |  | | | |  | | | |  | | | |  | | |  |  | |  | | |  | |  |  |  | |  | |  |  |  |  |
| Mild | 7 | | | | 8 (38) | | | | 1 | | | | 1 (20) | | | 0 | 0 (0) | | 0 | | | 0 (0) | | 5 | 6 (60) | 1 | | 1 (33) | | 3 | 3 (75) | 3 | 3 (75) |
| Moderate | 12 | | | | 13 (61∙9) | | | | 3 | | | | 4 (80) | | | 1 | 1 (100) | | 2 | | | 2 (100) | | 4 | 4 (40) | 2 | | 2 (66∙7) | | 1 | 1 (25) | 1 | 1 (25) |
| **Mouth ulcer** |  | | | |  | | | |  | | | |  | | |  |  | |  | | |  | |  |  |  | |  | |  |  |  |  |
| Mild | 4 | | | | 4 (80) | | | | 0 | | | | 0 (0) | | | 0 | 0 (0) | | 1 | | | 1 (100) | | 2 | 2 (100) | 1 | | 1 (50) | | 1 | 1 (50) | 0 | 0 (0) |
| Moderate | 1 | | | | 1 (20) | | | | 0 | | | | 0 (0) | | | 0 | 0 (0) | | 0 | | | 0 (0) | | 0 | 0 (0) | 1 | | 1 (50) | | 1 | 1 (50) | 0 | 0 (0) |
| **Skin lesion** |  | | | |  | | | |  | | | |  | | |  |  | |  | | |  | |  |  |  | |  | |  |  |  |  |
| Mild | 6 | | | | 6 (75) | | | | 0 | | | | 0 (0) | | | 1 | 1 (100) | | 1 | | | 1 (100) | | 0 | 0 (0) | 4 | | 4 (80) | | 1 | 1 (100) | 2 | 2 (100) |
| Moderate | 2 | | | | 2 (25) | | | | 0 | | | | 0 (0) | | | 0 | 0 (0) | | 0 | | | 0 (0) | | 1 | 1 (100) | 1 | | 1 (20) | | 0 | 0 (0) | 0 | 0 (0) |
| **Blister** |  | | | |  | | | |  | | | |  | | |  |  | |  | | |  | |  |  |  | |  | |  |  |  |  |
| Mild | 1 | | | | 1 (100) | | | | 0 | | | | 0 (0) | | | 0 | 0 (0) | | 0 | | | 0 (0) | | 0 | 0 (0) | 1 | | 1 (100) | | 0 | 0 (0) | 0 | 0 (0) |
| Moderate | 0 | | | | 0 (0) | | | | 0 | | | | 0 (0) | | | 0 | 0 (0) | | 0 | | | 0 (0) | | 0 | 0 (0) | 0 | | 0 (0) | | 0 | 0 (0) | 0 | 0 (0) |
| **Gastrointestinal symptoms** | | | | | | | | | | | | | | | | | | | | | | | | | | | | | |  |  |  |  |
| Mild | 24 | | | | 27 (77) | | | | 3 | | | | 3 (100) | | | 3 | 3 (50) | | 7 | | | 8 (73) | | 8 | 10 (83) | 3 | | 3 (100) | | 9 | 12 (80) | 6 | 10 (77) |
| Moderate | 5 | | | | 8 (23) | | | | 0 | | | | 0 (0) | | | 1 | 3 (50) | | 3 | | | 3 (27) | | 1 | 2 (17) | 0 | | 0 (0) | | 2 | 3 (20) | 3 | 3 (23) |
| **Unsolicited adverse events** | | | | | | | | | | | | | | | | | | | | | | | | | | | | | |  |  |  |  |
| **Malaria** |  | | | | | | | | | | | | | | | | | | | | | | | | | | | | |  |  |  |  |
| Mild | 6 | | | | | 6 (29) | | | | 1 | | | 1 (100) | | | 0 | 0 (0) | | 0 | | | 0 (0) | | 0 | 0 (0) | 5 | | 5 (56) | | 0 | 0 (0) | 2 | 2 (67) |
| Moderate | 12 | | | | | 15 (71) | | | | 0 | | | 0 (0) | | | 0 | 0 (0) | | 5 | | | 6 (100) | | 3 | 5 (100) | 4 | | 4 (44) | | 2 | 2 (100) | 1 | 1 (33) |
| **Rhinitis** |  | | | | |  | | | |  | | |  | | |  |  | |  | | |  | |  |  |  | |  | |  |  |  |  |
| Mild | 10 | | | | | 10 (100) | | | | 2 | | | 2 (100) | | | 1 | 1 (100) | | 2 | | | 2 (100) | | 1 | 1 (100) | 4 | | 4 (100) | | 1 | 1 (100) | 3 | 3 (100) |
| Moderate | 0 | | | | | 0 (0) | | | | 0 | | | 0 (0) | | | 0 | 0 (0) | | 0 | | | 0 (0) | | 0 | 0 (0) | 0 | | 0 (0) | | 0 | 0 (0) | 0 | 0 (0) |
| **Cough** |  | | | | |  | | | |  | | |  | | |  |  | |  | | |  | |  |  |  | |  | |  |  |  |  |
| Mild | 12 | | | | | 14 (78) | | | | 4 | | | 4 (57) | | | 2 | 3 (75) | | 1 | | | 1 (100) | | 1 | 1 (100) | 4 | | 4 (100) | | 2 | 2 (100) | 0 | 0 (0) |
| Moderate | 4 | | | | | 4 (22) | | | | 3 | | | 3 (43) | | | 1 | 1 (25) | | 0 | | | 0 (0) | | 0 | 0 (0) | 0 | | 0 (0) | | 0 | 0 (0) | 0 | 0 (0) |
| **Other** |  | | | | |  | | | |  | | |  | | |  |  | |  | | |  | |  |  |  | |  | |  |  |  |  |
| Mild | 60 | | | | | 87 (64) | | | | 3 | | | 5 (42) | | | 6 | 11 (65) | | 16 | | | 22 (69) | | 18 | 25 (61) | 17 | | 24 (71) | | 8 | 11 (85) | 6 | 8 (100) |
| Moderate | 33 | | | | | 49 (36) | | | | 4 | | | 7 (58) | | | 5 | 6 (35) | | 7 | | | 10 (31) | | 9 | 16 (39) | 8 | | 10 (29) | | 2 | 2 (15) | 0 | 0 (0) |
| **N: Number of participants reporting at least one event per dose irrespective of grade.**  **E: Indicates all events reported per cohort**  **%: Percentage of events reported**  ***Already partially reported in the New England Journal of Medicine, DOI: 10∙1056/NEJMoa1502924 (3x10^5^ PFU n = 20 and 3x10^6^ PFU n = 19)∙** | | | | | | | | | | | | | | | | | | | | | | | | | | | | | | | | | |
