# Supplementary material for: Safety and immunogenicity of rVSVΔG-ZEBOV-GP Ebola vaccine in adults and children in Lambaréné, Gabon: A phase I randomised trial
Source: PLoS Med. 2017 Oct 6;14(10):e1002402. doi: 10.1371/journal.pmed.1002402 (PMC5630143; doi:10.1371/journal.pmed.1002402)
Supplement: S2 Table — (DOCX) [file pmed.1002402.s006.docx]

# S2 Table. Reactogenicity to rVSV-ZEBOV-GP vaccine until day 28 in vaccinees with baseline ZEBOV-specific antibodies∙

|  | Adults | | | | | | | | | | | | | | | | | | | | Children | | Adolescent | |
| --- | --- | --- | --- | --- | --- | --- | --- | --- | --- | --- | --- | --- | --- | --- | --- | --- | --- | --- | --- | --- | --- | --- | --- | --- |
| Adverse | **All adults** | | **3x10^3^ PFU, N=7** | | | | **3x10^4^ PFU, N=9** | | **3x10^5^ PFU, N=1** | | | | | **3x10^6^ PFU, N=9** | | | | **2x10^7^ PFU, n=9** | | | **2x10^7^PFU, N=2** | | **2x10^7^ PFU, N=0** | |
| Events | **N** | **E (%)** | **N** | | | **E (%)** | **N** | **E (%)** | **N** | | | **E (%)** | | **N** | | **E (%)** | | **N** | | **E (%)** | **N** | **E (%)** | **N** | **E (%)** |
| *All events* |  |  |  | | |  |  |  |  | | |  | |  | |  | |  | |  |  |  |  |  |
| Mild | 25 | 82 (73∙9) | 3 | | | 4 (66∙7) | 4 | 21 (75) | 1 | | | 4 (100) | | 9 | | 32 (71∙1) | | 8 | | 21 (75) | 2 | 5 (100) | 0 | 0 (0) |
| Moderate | 14 | 29 (26∙1) | 2 | | | 2 (33∙3) | 2 | 7 (25) | 0 | | | 0 (0) | | 6 | | 13 (28∙9) | | 4 | | 7 (25) | 0 | 0 (0) | 0 | 0 (0) |
| *Solicited injection site reactions* | | | | | | | | | | | | | | | | | | | | |  |  |  |  |
| Pain |  |  |  |  | | |  |  |  | |  | | | |  |  |  | |  | |  |  |  |  |
| Mild | 9 | 9 (90) | 0 | 0 (0) | | | 1 | 1 (100) | 0 | | 0 (0) | | | | 6 | 6 (100) | 2 | | 2 (66∙7) | | 0 | 0 (0) | 0 | 0 (0) |
| Moderate | 1 | 1 (10) | 0 | 0 (0) | | | 0 | 0 (0) | 0 | | 0 (0) | | | | 0 | 0 (0) | 1 | | 1 (33∙3) | | 0 | 0 (0) | 0 | 0 (0) |
| Swelling |  |  |  |  | | |  |  |  | |  | | | |  |  |  | |  | |  |  |  |  |
| Mild | 1 | 1 (100) | 0 | 0 (0) | | | 0 | 0 (0) | 0 | | 0 (0) | | | | 0 | 0 (0) | 1 | | 1 (100) | | 0 | 0 (0) | 0 | 0 (0) |
| Moderate | 0 | 0 (0) | 0 | 0 (0) | | | 0 | 0 (0) | 0 | | 0 (0) | | | | 0 | 0 (0) | 0 | | 0 (0) | | 0 | 0 (0) | 0 | 0 (0) |
| *Solicited systemic reactions* | | | | | | | | | | | | | | | | | | | | |  |  |  |  |
| Fatigue |  |  |  | |  | |  |  | |  | | |  | |  |  |  | |  | |  |  |  |  |
| Mild | 7 | 7 (63∙3) | 0 | | 0 (0) | | 2 | 2 (66∙7) | | 1 | | | 1 (100) | | 3 | 3 (50) | 1 | | 1 (100) | | 1 | 1 (100) | 0 | 0 (0) |
| Moderate | 4 | 4 (36∙4) | 0 | | 0 (0) | | 1 | 1 (33∙3) | | 0 | | | 0 (0) | | 3 | 3 (50) | 0 | | 0 (0) | | 0 | 0 (0) | 0 | 0 (0) |
| Headache |  |  |  | |  | |  |  | |  | | |  | |  |  |  | |  | |  |  |  |  |
| Mild | 12 | 13 (72∙2) | 1 | | 1 (100) | | 2 | 2 (50) | | 0 | | | 0 (0) | | 6 | 7 (77∙8) | 3 | | 3 (75) | | 1 | 1 (100) | 0 | 0 (0) |
| Moderate | 4 | 5 (27∙8) | 0 | | 0 (0) | | 1 | 2 (50) | | 0 | | | 0 (0) | | 2 | 2 (22∙2) | 1 | | 1 (25) | | 0 | 0 (0) | 0 | 0 (0) |
| Myalgia |  |  |  | |  | |  |  | |  | | |  | |  |  |  | |  | |  |  |  |  |
| Mild | 4 | 4 (66∙7) | 0 | | 0 (0) | | 1 | 1 (50) | | 0 | | | 0 (0) | | 1 | 1 (50) | 2 | | 2 (100) | | 0 | 0 (0) | 0 | 0 (0) |
| Moderate | 2 | 2 (33∙3) | 0 | | 0 (0) | | 1 | 1 (50) | | 0 | | | 0 (0) | | 1 | 1 (50) | 0 | | 0 (0) | | 0 | 0 (0) | 0 | 0 (0) |
| Subjective fever | | | | | | | | | | | | | | | | | | | | |  |  |  |  |
| Mild | 6 | 6 (100) | 0 | | 0 (0) | | 2 | 2 (100) | | 0 | | | 0 (0) | | 0 | 0 (0) | 4 | | 4 (100) | | 1 | 1 (100) | 0 | 0 (0) |
| Moderate | 0 | 0 (0) | 0 | | 0 (0) | | 0 | 0 (0) | | 0 | | | 0 (0) | | 0 | 0 (0) | 0 | | 0 (0) | | 0 | 0 (0) | 0 | 0 (0) |
| Objective fever | | | | | | | | | | | | | | | | | | |  | |  |  |  |  |
| Mild | 2 | 2 (66∙7) | 0 | | | 0 (0) | 0 | 0 (0) | | 0 | | | 0 (0) | | 0 | 0 (0) | 2 | | 2 (100) | | 0 | 0 (0) | 0 | 0 (0) |
| Moderate | 1 | 1 (33∙3) | 0 | | | 0 (0) | 0 | 0 (0) | | 0 | | | 0 (0) | | 1 | 1 (100) | 0 | | 0 (0) | | 0 | 0 (0) | 0 | 0 (0) |
| Chills |  |  |  | | |  |  |  | |  | | |  | |  |  |  | |  | |  |  |  |  |
| Mild | 1 | 1 (100) | 0 | | | 0 (0) | 0 | 0 (0) | | 0 | | | 0 (0) | | 1 | 1 (100) | 0 | | 0 (0) | | 0 | 0 (0) | 0 | 0 (0) |
| Moderate | 0 | 0 (0) | 0 | | | 0 (0) | 0 | 0 (0) | | 0 | | | 0 (0) | | 0 | 0 (0) | 0 | | 0 (0) | | 0 | 0 (0) | 0 | 0 (0) |
| Arthralgia |  |  |  | | |  |  |  | |  | | |  | |  |  |  | |  | |  |  |  |  |
| Mild | 5 | 6 (60) | 0 | | | 0 (0) | 2 | 3 (60) | | 0 | | | 0 (0) | | 3 | 3 (75) | 0 | | 0 (0) | | 0 | 0 (0) | 0 | 0 (0) |
| Moderate | 4 | 4 (40) | 0 | | | 0 (0) | 2 | 2 (40) | | 0 | | | 0 (0) | | 1 | 1 (25) | 1 | | 1 (100) | | 0 | 0 (0) | 0 | 0 (0) |
| Mouth ulcer |  |  |  | | |  |  |  | |  | | |  | |  |  |  | |  | |  |  |  |  |
| Mild | 0 | 0 (0) | 0 | | | 0 (0) | 0 | 0 (0) | | 0 | | | 0 (0) | | 0 | 0 (0) | 0 | | 0 (0) | | 0 | 0 (0) | 0 | 0 (0) |
| Moderate | 0 | 0 (0) | 0 | | | 0 (0) | 0 | 0 (0) | | 0 | | | 0 (0) | | 0 | 0 (0) | 0 | | 0 (0) | | 0 | 0 (0) | 0 | 0 (0) |
| Skin lesion |  |  |  | | |  |  |  | |  | | |  | |  |  |  | |  | |  |  |  |  |
| Mild | 2 | 2 (100) | 0 | | | 0 (0) | 1 | 1 (100) | | 1 | | | 1 (100) | | 0 | 0 (0) | 0 | | 0 (0) | | 0 | 0 (0) | 0 | 0 (0) |
| Moderate | 0 | 0 (0) | 0 | | | 0 (0) | 0 | 0 (0) | | 0 | | | 0 (0) | | 0 | 0 (0) | 0 | | 0 (0) | | 0 | 0 (0) | 0 | 0 (0) |
| Blister |  |  |  | | |  |  |  | |  | | |  | |  |  |  | |  | |  |  |  |  |
| Mild | 0 | 0 (0) | 0 | | | 0 (0) | 0 | 0 (0) | | 0 | | | 0 (0) | | 0 | 0 (0) | 0 | | 0 (0) | | 0 | 0 (0) | 0 | 0 (0) |
| Moderate | 0 | 0 (0) | 0 | | | 0 (0) | 0 | 0 (0) | | 0 | | | 0 (0) | | 0 | 0 (0) | 0 | | 0 (0) | | 0 | 0 (0) | 0 | 0 (0) |
| Gastrointestinal symptoms | | | | | | | | | | | | | | | | | | | | |  |  |  |  |
| Mild | 10 | 10 (100) | 0 | | | 0 (0) | 3 | 3 (100) | | 1 | | | 1 (100) | | 3 | 3 (100) | 3 | | 3 (100) | | 1 | 1 (100) | 0 | 0 (0) |
| Moderate | 0 | 0 (0) | 0 | | | 0 (0) | 0 | 0 (0) | | 0 | | | 0 (0) | | 0 | 0 (0) | 0 | | 0 (0) | | 0 | 0 (0) | 0 | 0 (0) |
| *Unsolicited adverse events* | | | | | | | | | | | | | | | | | | | | |  |  |  |  |
| Malaria |  | | | | | | | | | | | | | | | | | | | |  |  |  |  |
| Mild | 1 | 1 (100) | 0 | | | 0 (0) | 0 | 0 (0) | | 0 | | | 0 (0) | | 0 | 0 (0) | 1 | | 1 (100) | | 0 | 0 (0) | 0 | 0 (0) |
| Moderate | 0 | 0 (0) | 0 | | | 0 (0) | 0 | 0 (0) | | 0 | | | 0 (0) | | 0 | 0 (0) | 0 | | 0 (0) | | 0 | 0 (0) | 0 | 0 (0) |
| Rhinitis |  |  |  | | |  |  |  | |  | | |  | |  |  |  | |  | |  |  |  |  |
| Mild | 1 | 1 (100) | 0 | | | 0 (0) | 0 | 0 (0) | | 0 | | | 0 (0) | | 1 | 1 (100) | 0 | | 0 (0) | | 0 | 0 (0) | 0 | 0 (0) |
| Moderate | 0 | 0 (0) | 0 | | | 0 (0) | 0 | 0 (0) | | 0 | | | 0 (0) | | 0 | 0 (0) | 0 | | 0 (0) | | 0 | 0 (0) | 0 | 0 (0) |
| Cough |  |  |  | | |  |  |  | |  | | |  | |  |  |  | |  | |  |  |  |  |
| Mild | 2 | 2 (100) | 1 | | | 1 (100) | 1 | 1 (100) | | 0 | | | 0 (0) | | 0 | 0 (0) | 0 | | 0 (0) | | 1 | 1 (100) | 0 | 0 (0) |
| Moderate | 0 | 0 (0) | 0 | | | 0 (0) | 0 | 0 (0) | | 0 | | | 0 (0) | | 0 | 0 (0) | 0 | | 0 (0) | | 0 | 0 (0) | 0 | 0 (0) |
| Other |  |  |  | | |  |  |  | |  | | |  | |  |  |  | |  | |  |  |  |  |
| Mild | 9 | 13 (54∙2) | 1 | | | 1 (50) | 2 | 4 (80) | | 1 | | | 1 (100) | | 4 | 6 (50) | 1 | | 1 (25) | | 0 | 0 (0) | 0 | 0 (0) |
| Moderate | 8 | 11 (45∙8) | 1 | | | 1 (50) | 1 | 1 (20) | | 0 | | | 0 (0) | | 4 | 6 (50) | 2 | | 3 (75) | | 0 | 0 (0) | 0 | 0 (0) |
| N: Number of subjects who reported at least one event. All events are reported per vaccine dose.  E: Number of all events reported  %: Percentage of all events reported | | | | | | | | | | | | | | | | | | | | | | | | |
