# Supplementary material for: Safety and immunogenicity of rVSVΔG-ZEBOV-GP Ebola vaccine in adults and children in Lambaréné, Gabon: A phase I randomised trial
Source: PLoS Med. 2017 Oct 6;14(10):e1002402. doi: 10.1371/journal.pmed.1002402 (PMC5630143; doi:10.1371/journal.pmed.1002402)
Supplement: S3 Table — (DOCX) [file pmed.1002402.s007.docx]

# S3 Table. Reactogenicity to rVSV-ZEBOV-GP vaccine until day 28 in vaccinees without baseline ZEBOV-specific antibodies∙

|  | Adults | | | | | | | | | | | | | | | | | | | | | | | | | | | | | | | | Children | | | Adolescent | | | |
| --- | --- | --- | --- | --- | --- | --- | --- | --- | --- | --- | --- | --- | --- | --- | --- | --- | --- | --- | --- | --- | --- | --- | --- | --- | --- | --- | --- | --- | --- | --- | --- | --- | --- | --- | --- | --- | --- | --- | --- |
| Adverse | **All adults** | | | | | | | | | **3x10^3^ PFU, n=13** | | | | | | | **3x10^4^ PFU, n=11** | | | | **3x10^5^ PFU, n=19** | | | | | **3x10^6^ PFU, n=28** | | | | **2x10^7^ PFU, n=7** | | | **2x10^7^ PFU, n=18** | | | **2x10^7^ PFU, n=20** | | | |
| Events | **N** | | | | | **E (%)** | | | | **N** | | | | | | **E (%)** | **N** | | | **E (%)** | **N** | | | **E (%)** | | **N** | | **E (%)** | | **N** | | **E (%)** | **N** | **E (%)** | | **N** | | **E (%)** | |
| ***Any event*** |  | | | | |  | | | |  | | | | | |  |  | | |  |  | | |  | |  | |  | |  | |  |  |  | |  | |  | |
| Mild | 76 | | | | | 234 (68) | | | | 12 | | | | | | 33 (63∙5) | 10 | | | 26 (66∙7) | 19 | | | 54 (65∙1) | | 28 | | 97 (70∙3) | | 7 | | 24 (75) | 18 | 87 (82∙1) | | 20 | | 79 (80∙6) | |
| Moderate | 40 | | | | | 110 (32) | | | | 7 | | | | | | 19 (36∙5) | 6 | | | 13 (33∙3) | 11 | | | 29 (34∙9) | | 13 | | 41 (29∙7) | | 3 | | 8 (25) | 7 | 19 (17∙9) | | 6 | | 19 (19∙4) | |
| ***Solicited injection site reactions*** | | | | | | | | | | | | | | | | | | | | | | | | | | | | | | | | |  |  |  | |  | |  |
| **Pain** | |  | | | |  | | | |  | | | |  | | |  | | |  |  | |  | | | |  |  |  | |  | |  |  | |  | |  | |
| Mild | | 29 | | | | 32 (91∙4) | | | | 3 | | | | 4 (100) | | | 3 | | | 3 (75) | 3 | | 3 (100) | | | | 16 | 16 (100) | 4 | | 6 (75) | | 9 | 9 (75) | | 8 | | 8 (80) | |
| Moderate | | 3 | | | | 3 (8∙6) | | | | 0 | | | | 0 (0) | | | 1 | | | 1 (25) | 0 | | 0 (0) | | | | 0 | 0 (0) | 2 | | 2 (25) | | 2 | 3 (25) | | 2 | | 2 (20) | |
| **Swelling** | |  | | | |  | | | |  | | | |  | | |  | | |  |  | |  | | | |  |  |  | |  | |  |  | |  | |  | |
| Mild | | 0 | | | | 0 (0) | | | | 0 | | | | 0 (0) | | | 0 | | | 0 (0) | 0 | | 0 (0) | | | | 0 | 0 (0) | 0 | | 0 (0) | | 0 | 0 (0) | | 1 | | 100 (0) | |
| Moderate | | 0 | | | | 0 (0) | | | | 0 | | | | 0 (0) | | | 0 | | | 0 (0) | 0 | | 0 (0) | | | | 0 | 0 (0) | 0 | | 0 (0) | | 0 | 0 (0) | | 0 | | 0 (0) | |
| ***Solicited systemic reactions*** | | | | | | | | | | | | | | | | | | | | | | | | | | | | | | | | |  |  | |  | |  | |
| **Fatigue** | | |  | | | |  | | | |  | | | |  | | |  | |  | |  | | |  | |  |  |  | |  | |  |  | |  | |  | |
| Mild | | | 24 | | | | 26 (72∙2) | | | | 3 | | | | 3 (75) | | | 3 | | 3 (100) | | 7 | | | 7 (70) | | 9 | 11 (68∙8) | 2 | | 2 (66∙7) | | 9 | 10 (83∙3) | | 3 | | 3 (42∙9) | |
| Moderate | | | 10 | | | | 10 (27∙8) | | | | 1 | | | | 1 (25) | | | 0 | | 0 (0) | | 3 | | | 3 (30) | | 5 | 5 (31∙2) | 1 | | 1 (33∙3) | | 2 | 2 (16∙7) | | 4 | | 4 (57∙1) | |
| **Headache** | | |  | | | |  | | | |  | | | |  | | |  | |  | |  | | |  | |  |  |  | |  | |  |  | |  | |  | |
| Mild | | | 26 | | | | 32 (68∙1) | | | | 5 | | | | 6 (60) | | | 3 | | 5 (100) | | 6 | | | 7 (63∙6) | | 9 | 10 (62∙5) | 3 | | 4 (80) | | 8 | 9 (90) | | 11 | | 13 (81∙2) | |
| Moderate | | | 13 | | | | 15 (31∙9) | | | | 3 | | | | 4 (40) | | | 0 | | 0 (0) | | 3 | | | 4 (36∙4) | | 6 | 6 (37∙5) | 1 | | 1 (20) | | 1 | 1 (10) | | 3 | | 3 (18∙8) | |
| **Myalgia** | | |  | | | |  | | | |  | | | |  | | |  | |  | |  | | |  | |  |  |  | |  | |  |  | |  | |  | |
| Mild | | | 9 | | | | 9 (64∙3) | | | | 2 | | | | 2 (66∙7) | | | 0 | | 0 (0) | | 1 | | | 1 (50) | | 6 | 6 (66∙7) | 0 | | 0 (0) | | 3 | 3 (75) | | 5 | | 5 (83∙3) | |
| Moderate | | | 5 | | | | 5 (35∙7) | | | | 1 | | | | 1 (33∙3) | | | 0 | | 0 (0) | | 1 | | | 1 (50) | | 3 | 3 (33∙3) | 0 | | 0 (0) | | 1 | 1 (25) | | 1 | | 1 (16∙7) | |
| **Subjective fever** | | | | | | | | | | | | | | | | | | | | | | | | | | | | | | | | |  |  | |  | |  | |
| Mild | | | 16 | | | | 18 (78∙3) | | | | 1 | | | | 1 (50) | | | 2 | | 2 (50) | | 1 | | | 2 (100) | | 9 | 10 (83∙3) | 3 | | 3 (100) | | 7 | 9 (90) | | 7 | | 9 (90) | |
| Moderate | | | 5 | | | | 5 (21∙7) | | | | 1 | | | | 1 (50) | | | 2 | | 2 (50) | | 0 | | | 0 (0) | | 2 | 2 (16∙7) | 0 | | 0 (0) | | 1 | 1 (10) | | 1 | | 1 (10) | |
| **Objective fever** | | | | | | | | | | | | | | | | | | | | | | | | | | | | | | |  | |  |  | |  | |  | |
| Mild | | | | 10 | | | | 11 (84∙6) | | | | 3 | | | | 4 (100) | | | 1 | 1 (100) | | 1 | | | 1 (100) | | 3 | 3 (60) | 2 | | 2 (100) | | 7 | 7 (100) | | 5 | | 5 (100) | |
| Moderate | | | | 1 | | | | 2 (15∙4) | | | | 0 | | | | 0 (0) | | | 0 | 0 (0) | | 0 | | | 0 (0) | | 2 | 2 (40) | 0 | | 0 (0) | | 0 | 0 (0) | | 0 | | 0 (0) | |
| **Chills** | | | |  | | | |  | | | |  | | | |  | | |  |  | |  | | |  | |  |  |  | |  | |  |  | |  | |  | |
| Mild | | | | 3 | | | | 3 (100) | | | | 0 | | | | 0 (0) | | | 0 | 0 (0) | | 0 | | | 0 (0) | | 3 | 3 (100) | 0 | | 0 (0) | | 5 | 5 (100) | | 3 | | 3 (100) | |
| Moderate | | | | 0 | | | | 0 (0) | | | | 0 | | | | 0 (0) | | | 0 | 0 (0) | | 0 | | | 0 (0) | | 0 | 0 (0) | 0 | | 0 (0) | | 0 | 0 (0) | | 0 | | 0 (0) | |
| **Arthralgia** | | | |  | | | |  | | | |  | | | |  | | |  |  | |  | | |  | |  |  |  | |  | |  |  | |  | |  | |
| Mild | | | | 7 | | | | 8 (38∙1) | | | | 1 | | | | 1 (20) | | | 0 | 0 (0) | | 0 | | | 0 (0) | | 5 | 6 (60) | 1 | | 1 (33∙3) | | 3 | 3 (75) | | 3 | | 3 (75) | |
| Moderate | | | | 12 | | | | 13 (61∙9) | | | | 3 | | | | 4 (80) | | | 1 | 1 (100) | | 2 | | | 2 (100) | | 4 | 4 (40) | 2 | | 2 (66∙7) | | 1 | 1 (25) | | 1 | | 1 (25) | |
| **Mouth ulcer** | | | |  | | | |  | | | |  | | | |  | | |  |  | |  | | |  | |  |  |  | |  | |  |  | |  | |  | |
| Mild | | | | 4 | | | | 4 (80) | | | | 0 | | | | 0 (0) | | | 0 | 0 (0) | | 1 | | | 1 (100) | | 2 | 2 (100) | 1 | | 1 (50) | | 1 | 1 (50) | | 0 | | 0 (0) | |
| Moderate | | | | 1 | | | | 1 (20) | | | | 0 | | | | 0 (0) | | | 0 | 0 (0) | | 0 | | | 0 (0) | | 0 | 0 (0) | 1 | | 1 (50) | | 1 | 1 (50) | | 0 | | 0 (0) | |
| **Skin lesion** | | | |  | | | |  | | | |  | | | |  | | |  |  | |  | | |  | |  |  |  | |  | |  |  | |  | |  | |
| Mild | | | | 4 | | | | 4 (80) | | | | 0 | | | | 0 (0) | | | 0 | 0 (0) | | 0 | | | 0 (0) | | 0 | 0 (0) | 4 | | 4 (80) | | 1 | 1 (100) | | 2 | | 2 (100) | |
| Moderate | | | | 1 | | | | 1 (20) | | | | 0 | | | | 0 (0) | | | 0 | 0 (0) | | 0 | | | 0 (0) | | 0 | 0 (0) | 1 | | 1 (20) | | 0 | 0 (0) | | 0 | | 0 (0) | |
| Blister | | | |  | | | |  | | | |  | | | |  | | |  |  | |  | | |  | |  |  |  | |  | |  |  | |  | |  | |
| Mild | | | | 1 | | | | 1 (100) | | | | 0 | | | | 0 (0) | | | 0 | 0 (0) | | 0 | | | 0 (0) | | 0 | 0 (0) | 1 | | 1 (100) | | 0 | 0 (0) | | 0 | | 0 (0) | |
| Moderate | | | | 0 | | | | 0 (0) | | | | 0 | | | | 0 (0) | | | 0 | 0 (0) | | 0 | | | 0 (0) | | 0 | 0 (0) | 0 | | 0 (0) | | 0 | 0 (0) | | 0 | | 0 (0) | |
| **Gastrointestinal symptoms** | | | | | | | | | | | | | | | | | | | | | | | | | | | | | | | | |  |  | |  | |  | |
| Mild | | | | 24 | | | | 27 (77∙1) | | | | 3 | | | | 3 (100) | | | 3 | 3 (50) | | 7 | | | 8 (72∙7) | | 8 | 10 (83∙3) | 3 | | 3 (100) | | 9 | 12 (80) | | 6 | | 10 (76∙9) | |
| Moderate | | | | 5 | | | | 8 (22∙9) | | | | 0 | | | | 0 (0) | | | 1 | 3 (50) | | 3 | | | 3 (27∙3) | | 1 | 2 (16∙7) | 0 | | 0 (0) | | 2 | 3 (20) | | 3 | | 3 (23∙1) | |
| ***Unsolicited adverse events*** | | | | | | | | | | | | | | | | | | | | | | | | | | | | | | | | |  |  | |  | |  | |
| **Malaria** | | | | |  | | | | | | | | | | | | | | | | | | | | | | | | | | | |  |  | |  | |  | |
| Mild | | | | | 4 | | | | 4 (23∙5) | | | | 1 | | | 1 (100) | | | 0 | 0 (0) | | 0 | | | 0 (0) | | 0 | 0 (0) | 3 | | 3 (42∙9) | | 0 | 0 (0) | | 2 | | 2 (66∙7) | |
| Moderate | | | | | 11 | | | | 13 (76∙5) | | | | 0 | | | 0 (0) | | | 0 | 0 (0) | | 5 | | | 6 (100) | | 2 | 3 (100) | 4 | | 4 (57∙1) | | 2 | 2 (100) | | 1 | | 1 (33∙3) | |
| **Rhinitis** | | | | |  | | | |  | | | |  | | |  | | |  |  | |  | | |  | |  |  |  | |  | |  |  | |  | |  | |
| Mild | | | | | 9 | | | | 9 (100) | | | | 2 | | | 2 (100) | | | 1 | 1 (100) | | 2 | | | 2 (100) | | 0 | 0 (0) | 4 | | 4 (100) | | 1 | 1 (100) | | 3 | | 3 (100) | |
| Moderate | | | | | 0 | | | | 0 (0) | | | | 0 | | | 0 (0) | | | 0 | 0 (0) | | 0 | | | 0 (0) | | 0 | 0 (0) | 0 | | 0 (0) | | 0 | 0 (0) | | 0 | | 0 (0) | |
| **Cough** | | | | |  | | | |  | | | |  | | |  | | |  |  | |  | | |  | |  |  |  | |  | |  |  | |  | |  | |
| Mild | | | | | 7 | | | | 8 (66∙7) | | | | 2 | | | 2 (40) | | | 2 | 2 (66∙7) | | 0 | | | 0 (0) | | 0 | 0 (0) | 3 | | 4 (100) | | 1 | 1 (100) | | 0 | | 0 (0) | |
| Moderate | | | | | 4 | | | | 4 (33∙3) | | | | 3 | | | 3 (60) | | | 1 | 1 (33∙3) | | 0 | | | 0 (0) | | 0 | 0 (0) | 0 | | 0 (0) | | 0 | 0 (0) | | 0 | | 0 (0) | |
| **Other** | | | | |  | | | |  | | | |  | | |  | | |  |  | |  | | |  | |  |  |  | |  | |  |  | |  | |  | |
| Mild | | | | | 51 | | | | 74 (67∙3) | | | | 3 | | | 5 (45∙5) | | | 4 | 7 (58∙3) | | 15 | | | 22 (68∙8) | | 13 | 18 (64∙3) | 16 | | 22 (81∙5) | | 8 | 11 (84∙6) | | 6 | | 8 (100) | |
| Moderate | | | | | 24 | | | | 36 (32∙7) | | | | 3 | | | 6 (54∙5) | | | 4 | 5 (41∙7) | | 7 | | | 10 (31∙2) | | 5 | 10 (35∙7) | 5 | | 5 (18∙5) | | 2 | 2 (15∙4) | | 0 | | 0 (0) | |
| N: Number of participants reporting at least one event∙ All events are reported for each dose  E: Number of all events reported  %: Percentage of all events reported | | | | | | | | | | | | | | | | | | | | | | | | | | | | | | | | | | | | | | | |
