# Supplementary material for: Safety and immunogenicity of rVSVΔG-ZEBOV-GP Ebola vaccine in adults and children in Lambaréné, Gabon: A phase I randomised trial
Source: PLoS Med. 2017 Oct 6;14(10):e1002402. doi: 10.1371/journal.pmed.1002402 (PMC5630143; doi:10.1371/journal.pmed.1002402)
Supplement: S4 Table — (DOCX) [file pmed.1002402.s008.docx]

| S4 Table. Frequency of symptoms post day 28 in all adult cohorts | | | | | |
| --- | --- | --- | --- | --- | --- |
| Symptoms | **D56**  **(two months)** | **D84**  **(three months)** | **D180**  **(six months)** | **Unscheduled visits after D28** |  |
| Abdominal pain | 3 | 3 | 2 | 9 |  |
| Allergic Rhinitis | 1 | 0 | 0 | 0 |  |
| Anal Abscess | 0 | 0 | 1 | 0 |  |
| Anorexia | 3 | 0 | 3 | 3 |  |
| Aphtha | 0 | 1 | 0 | 1 |  |
| Arthralgia | 1 | 1 | 4 | 7 |  |
| Asthenia | 0 | 0 | 0 | 3 |  |
| Back pain | 2 | 3 | 1 | 2 |  |
| Blurred vision | 0 | 1 | 0 | 1 |  |
| Buttock pain | 0 | 1 | 0 | 0 |  |
| Chest pain | 1 | 0 | 0 | 6 |  |
| Chills | 0 | 0 | 0 | 2 |  |
| Common cold | 1 | 0 | 1 | 0 |  |
| Conjunctival pallor | 0 | 0 | 0 | 1 |  |
| Conjunctivitis | 1 | 1 | 0 | 0 |  |
| Constipation | 2 | 0 | 0 | 1 |  |
| Contact dermatitis | 0 | 1 | 0 | 0 |  |
| Cough | 6 | 5 | 1 | 4 |  |
| Dental caries | 5 | 1 | 1 | 3 |  |
| Diarrhoea | 3 | 2 | 0 | 3 |  |
| Dysentery | 0 | 0 | 0 | 1 |  |
| Dysgeusia | 0 | 0 | 0 | 1 |  |
| Dysuria | 2 | 0 | 1 | 1 |  |
| Enteritis | 1 | 0 | 0 | 0 |  |
| Eye Pruritus | 0 | 0 | 0 | 1 |  |
| Face injury | 0 | 1 | 0 | 0 |  |
| Fatigue | 1 | 1 | 1 | 5 |  |
| Fever | 4 | 1 | 2 | 6 |  |
| Fractured Nose | 0 | 0 | 0 | 1 |  |
| Genital pain | 1 | 0 | 0 | 0 |  |
| Gingivitis | 0 | 2 | 0 | 0 |  |
| Haematuria | 0 | 0 | 0 | 1 |  |
| Headache | 8 | 8 | 2 | 12 |  |
| Hypertensive crisis | 1 | 0 | 0 | 0 |  |
| Injection site tingling | 0 | 1 | 0 | 0 |  |
| Injury to finger | 0 | 0 | 1 | 0 |  |
| Insomnia | 0 | 1 | 1 | 1 |  |
| Knee injury | 1 | 0 | 0 | 0 |  |
| Libido decreased | 1 | 0 | 0 | 0 |  |
| Malaise | 0 | 0 | 0 | 1 |  |
| Malaria | 0 | 0 | 1 | 0 |  |
| Menometrorrhagia | 1 | 0 | 0 | 0 |  |
| Metrorrhagia | 0 | 0 | 0 | 1 |  |
| Myalgia | 2 | 0 | 0 | 1 |  |
| Mycosis | 0 | 2 | 0 | 0 |  |
| Nasal Obstruction | 3 | 0 | 0 | 1 |  |
| Nausea | 0 | 0 | 1 | 1 |  |
| Neck pain | 0 | 0 | 0 | 1 |  |
| Palpitation | 0 | 0 | 1 | 0 |  |
| Peripheral swelling | 1 | 0 | 0 | 0 |  |
| Pruritus | 1 | 4 | 0 | 1 |  |
| Rash | 0 | 1 | 0 | 1 |  |
| Rhinitis | 5 | 2 | 2 | 3 |  |
| Rhinorrhoea | 0 | 1 | 0 | 1 |  |
| Schistosomiasis | 1 | 0 | 0 | 0 |  |
| Sinusitis | 0 | 1 | 0 | 0 |  |
| Skin tumor | 0 | 0 | 1 | 0 |  |
| Subcutaneous abscess | 2 | 0 | 0 | 0 |  |
| Throat pain | 1 | 1 | 0 | 3 |  |
| Tinea capitis | 0 | 0 | 0 | 1 |  |
| Toe injury | 0 | 0 | 0 | 1 |  |
| Tongue ulceration | 0 | 1 | 0 | 0 |  |
| Tooth abscess | 0 | 1 | 0 | 0 |  |
| Tooth ache | 1 | 2 | 2 | 0 |  |
| Upper respiratory tract infection (u∙r∙t∙i) | 1 | 0 | 0 | 0 |  |
| Vertigo | 2 | 0 | 0 | 1 |  |
| Vomiting | 0 | 0 | 0 | 2 |  |
| Weight loss | 1 | 0 | 0 | 0 |  |
| Wound | 1 | 3 | 2 | 2 |  |
| Tables shows the frequency of adverse events reported from day 56 to day 180 regardless of the dose administered to adults. All classified as mild to moderate severity and resolved without sequelae. | | | | |  |
