# Supplementary material for: Safety and immunogenicity of rVSVΔG-ZEBOV-GP Ebola vaccine in adults and children in Lambaréné, Gabon: A phase I randomised trial
Source: PLoS Med. 2017 Oct 6;14(10):e1002402. doi: 10.1371/journal.pmed.1002402 (PMC5630143; doi:10.1371/journal.pmed.1002402)
Supplement: S5 Table — (DOCX) [file pmed.1002402.s009.docx]

# S5 Table. Haematology and biochemistry parameters

| Adults | | | | | | Children | Adolescents |
| --- | --- | --- | --- | --- | --- | --- | --- |
|  | 3x10^3^ PFU | 3x10^4^ PFU | 3x10^5^ PFU | 3x10^6^ PFU | 2x10^7^ PFU | 2x10^7^ PFU | 2x10^7^ PFU |
| Haemoglobin, G/L |  |  |  |  |  |  |  |
| Screening | 134∙3 (108-167) | 136∙6 (113-167) | 135∙2 (96-168) | 140 (113-162) | 140∙8 (101-169) | 113 (97-138) | 121∙3 (94-147) |
| D1 | 132∙2 (103-157) | 131∙2 (99-160) | 130∙9 (97-167) | 136∙5 (105-162) | 136∙6 (102-159) | 126∙5 (126-127) | 123∙1 (100-138) |
| D2/3 | 132∙7 (100-150) | 131∙8 (100-159) | 133∙8 (98-170) | 136∙9 (104-159) | 136∙8 (94-164) | 108∙7 (87-120) | 126∙1 (97-144) |
| D7 | 132∙8 (99-151) | 133∙2 (101-162) | 134∙2 (98-176) | 140∙6 (114-173) | 139∙1 (102-166) | 112∙2 (96-127) | 126∙8 (111-140) |
| D28 | 127∙5 (104-149) | 132∙7 (107-150) | 134∙2 (99-177) | 137∙1 (106-162) | 135∙6 (98-164) | 112∙3 (92-132) | - |
| D180 | 133∙4 (106-154) | 133∙1 (110-152) | 135∙3 (112-171) | 134∙6 (93-163) | 136∙6 (98-155) | - | 118∙9 (94-138) |
| Platelets, G/L |  |  |  |  |  |  |  |
| Screening | 193∙2 (119-260) | 243∙5 (166-356) | 202∙7 (112-352) | 215∙1 (111-380) | 240∙2 (129-416) | 268∙4 (125-395) | 260∙2 (160-410) |
| D1 | 206∙5 (131-262) | 241∙3 (162-391) | 192∙7 (91-322) | 185∙8 (72-304) | 205∙1 (116-361) | 235∙5 (220-251) | 205∙2 (130-310) |
| D2/3 | 199∙8 (135-263) | 228∙4 (139-356) | 186∙2 (71-328) | 190∙1 (97-350) | 188∙9 (106-304) | 203∙6 (86-332) | 220∙7 (131-331) |
| D7 | 197∙8 (133-280) | 215∙3 (133-349) | 194∙9 (105-351) | 185∙2 (74-278) | 204∙4 (118-320) | 255∙8 (112-400) | 222∙8 (97-291) |
| D28 | 198∙9 (118-368) | 230∙7 (123-365) | 195∙9 (105-296) | 215∙3 (107-325) | 223∙9 (113-413) | 266∙7 (110-388) | - |
| D180 | 207∙2 (123-328) | 212∙9 (136-286) | 199∙1 (83-323) | 202∙2 (95-298) | 208∙5 (66-345) | - | 236∙6 (113-393) |
| Leucocyte, G/L |  |  |  |  |  |  |  |
| Screening | 6∙3 (3∙9-10) | 6∙6 (3∙7-9∙2) | 6∙3 (4∙5-10∙1) | 6∙7 (3∙8-10∙5) | 6∙6 (3∙6-11∙6) | 8∙5 (5∙7-16∙1) | 6∙5 (3∙4-10∙6) |
| D1 | 5∙8 (3-9∙3) | 5∙8 (3∙9-8∙5) | 5∙7 (3∙9-11∙1) | 5∙9 (2∙7-10∙1) | 5∙6 (3∙5-8∙3) | 6∙5 (6∙4-6∙6) | 5∙1 (3∙1-9) |
| D2/3 | 5∙7 (3∙3-8∙4) | 5∙6 (3∙2-8∙3) | 5∙2 (3∙6-8∙9) | 5∙2 (2∙5-8∙5) | 5∙3 (3∙2-8∙1) | 6∙5 (3∙1-14∙3) | 4∙9 (2∙8-8∙4) |
| D7 | 5∙3 (2∙4-9∙1) | 4∙9 (3∙2-7∙1) | 5∙6 (3∙7-9∙7) | 5∙3 (2∙2-8∙9) | 4∙7 (2∙8-8∙4) | 6∙4 (3∙6-13∙4) | 5∙8 (3∙8-8∙8) |
| D28 | 5∙4 (2∙8-12∙4) | 5∙6 (3∙3-9∙2) | 5∙5 (3∙9-9∙2) | 6 (3∙2-9∙6) | 5∙6 (3∙8-11∙3) | 7∙2 (4∙9-19∙8) | - |
| D180 | 4∙5 (3∙6-5∙9) | 6∙4 (3∙9-15∙3) | 5∙6 (4-8∙7) | 5∙8 (2∙7-9∙7) | 6∙2 (3∙1-10∙3) | - | 6∙5 (3∙6-9∙1) |
| Absolute Lymphocyte Count, G/L | | |  |  |  |  |  |
| Screening | 2∙4 (1∙5-3∙4) | 2∙3 (1∙5-3∙3) | 2∙3 (1∙5-4∙1) | 2∙3 (1∙2-3∙4) | 2∙8 (1∙6-5∙1) | 3∙8 (2∙3-5∙2) | 2∙6 (1∙7-4∙8) |
| D1 | 2∙3 (0∙8-3∙5) | 2∙2 (1∙4-3∙2) | 1∙8 (1∙1-2∙3) | 1∙2 (0∙7-2∙7) | 1∙3 (0∙8-2∙3) | 1∙7 (1∙1-2∙3) | 1∙7 (1∙2-3∙1) |
| D2/3 | 2∙3 (1∙1-3∙4) | 1∙9 (0∙9-3∙2) | 1∙9 (1∙2-2∙5) | 1∙8 (1∙1-3∙6) | 2 (1-4∙2) | 2∙8 (0∙2-5) | 2 (1∙3-3∙4) |
| D7 | 2 (0∙9-3∙7) | 2 (1∙3-3∙1) | 2∙1 (1∙5-2∙8) | 2 (1-3∙3) | 2 (1∙4-3∙7) | 2∙7 (1∙6-4∙7) | 2∙1 (1∙5-3) |
| D28 | 2∙2 (1∙3-3∙4) | 2∙2 (1∙5-2∙9) | 2 (1∙2-2∙9) | 2∙1 (0∙9-3∙2) | 2∙2 (1∙2-4∙9) | 2∙9 (1∙8-5∙4) | - |
| D180 | 1∙8 (1∙4-2∙4) | 2∙2 (1∙4-3∙1) | 2∙2 (1∙2-3∙6) | 2∙3 (1∙3-3∙1) | 2∙4 (1∙4-3∙2) | - | 2∙4 (1∙6-3∙8) |
| Absolute Neutrophil count, G/L | |  |  |  |  |  |  |
| Screening | 2∙6 (1∙5-4∙4) | 2∙8 (1∙3-3∙9) | 2∙7 (1∙3-6∙1) | 2∙6 (1∙6-4∙2) | 2∙2 (1∙1-3∙5) | 3∙7 (1∙7-7∙7) | 2∙6 (1∙2-3∙8) |
| D1 | 2∙3 (1∙1-4∙8) | 2∙2 (1∙1-3∙9) | 2∙5 (0∙8-7∙3) | 3∙1 (1∙2-5∙3) | 2∙9 (1∙7-4∙7) | 3∙6 (3∙5-3∙7) | 2∙1 (1-3∙7) |
| D2/3 | 2∙2 (0∙8-4∙1) | 2∙5 (1∙2-5∙1) | 2 (1∙1-3∙8) | 1∙8 (0∙8-3∙2) | 1∙9 (0∙6-3∙6) | 2∙4 (1∙1-5∙4) | 2∙1 (1∙2-4∙7) |
| D7 | 2∙3 (0∙7-4∙7) | 1∙8 (0∙9-3∙4) | 1∙9 (0∙4-4) | 1∙7 (0∙4-4∙6) | 1∙6 (0∙7-4∙7) | 2∙5 (1∙2-5∙6) | 2∙9 (1∙4-4∙6) |
| D28 | 1∙9 (0∙8-4) | 2∙2 (1-3∙4) | 2∙2 (1∙1-3∙7) | 2∙4 (1-5) | 2∙2 (1∙5-3∙6) | 2∙5 (1∙3-4∙4) | - |
| D180 | 1∙9 (1-2∙8) | 2∙2 (0-3∙5) | 2∙8 (1∙5-5∙4) | 2∙5 (0-6∙1) | 2∙9 (1∙5-4∙8) | - | 3 (1∙4-5∙5) |
| Absolute Monocyte Count, G/L | |  |  |  |  |  |  |
| Screening | 0∙6 (0∙3-1) | 0∙6 (0∙4-0∙8) | 0∙5 (0-0∙9) | 0∙5 (0∙2-0∙8) | 0∙5 (0∙3-1∙1) | 0∙1 (0-0∙8) | 0∙5 (0-0∙9) |
| D1 | 0∙5 (0∙2-0∙9) | 0∙5 (0∙3-0∙7) | 0∙5 (0∙2-1) | 0∙7 (0∙4-1∙2) | 0∙7 (0∙4-1∙1) | 0∙6 (0-1∙1) | 0∙8 (0∙5-1∙2) |
| D2/3 | 0∙4 (0-0∙7) | 0∙5 (0-1) | 0∙6 (0-1) | 0∙7 (0∙4-1∙2) | 0∙8 (0∙4-1∙2) | 0∙4 (0-1∙6) | 0∙3 (0-0∙8) |
| D7 | 0∙3 (0-0∙8) | 0∙4 (0-0∙8) | 0∙5 (0∙2-1∙1) | 0∙4 (0∙2-0∙8) | 0∙4 (0∙2-0∙7) | 0∙4 (0-1) | 0∙4 (0-0∙7) |
| D28 | 0∙4 (0-0∙8) | 0∙4 (0-0∙9) | 0∙4 (0∙3-1) | 0∙5 (0∙2-1) | 0∙5 (0∙3-1) | 0∙6 (0-1∙8) | - |
| D180 | 0∙4 (0-0∙8) | 0∙6 (0-1∙5) | 0∙3 (0-0∙8) | 0∙3 (0-1∙1) | 0∙3 (0-1∙2) | - | 0∙3 (0-0∙8) |
| Creatinine, mg/dL | |  |  |  |  |  |  |
| Screening | 0∙8 (0∙6-1∙1) | 0∙8 (0∙5-0∙9) | 0∙8 (0∙5-1) | 0∙8 (0∙5-1∙1) | 0∙8 (0∙5-1∙1) | 0∙4 (0∙3-0∙7) | 0∙6 (0∙3-1∙1) |
| D1 | 0∙8 (0∙5-1∙1) | 0∙8 (0∙6-1∙5) | 0∙7 (0∙5-1) | 0∙8 (0∙5-1∙2) | 0∙8 (0∙6-1) | 0∙4 (0∙4-0∙4) | 0∙6 (0∙3-1∙1) |
| D2/3 | 0∙8 (0∙5-1∙1) | 0∙8 (0∙5-1∙1) | 0∙7 (0∙5-0∙9) | 0∙8 (0∙5-1∙2) | 0∙8 (0∙6-1∙1) | 0∙5 (0∙4-0∙7) | 0∙6 (0∙4-0∙8) |
| D7 | 0∙8 (0∙5-1∙1) | 0∙8 (0∙5-1∙3) | 0∙8 (0∙5-1) | 0∙8 (0∙5-1∙1) | 0∙7 (0∙5-1) | 0∙5 (0∙4-0∙6) | 0∙7 (0∙5-0∙9) |
| D28 | 0∙8 (0∙6-1∙2) | 0∙8 (0∙5-1∙2) | 0∙8 (0∙5-1∙1) | 0∙8 (0∙5-1∙1) | 0∙8 (0∙6-1∙1) | - | - |
| D180 | 0∙8 (0∙5-1∙1) | 0∙9 (0∙7-1∙1) | 0∙8 (0∙6-1∙2) | 0∙8 (0∙4-1∙3) | 0∙9 (0∙1-1∙4) | - | - |
| ALT∙ U/L |  |  |  |  |  |  |  |
| Screening | 15∙5 (7∙8-32∙7) | 13∙8 (7∙6-23∙2) | 15∙1 (8∙4-29∙2) | 16 (6∙8-34∙2) | 16∙5 (7∙2-33∙5) | 15∙8 (7-62) | 11∙5 (0-19∙7) |
| D1 | 15∙4 (7∙4-28∙4) | 15∙9 (5∙9-27∙7) | 14 (8∙1-24∙4) | 17∙5 (8∙2-40∙8) | 31∙2 (6∙6-266) | 12 (12-12) | 12∙7 (6-20) |
| D2/3 | 17∙3 (8-30) | 15∙7 (6∙7-28∙1) | 13∙6 (8∙1-25) | 17∙2 (5-41∙3) | 23∙5 (6∙5-149) | 18∙4 (6-118) | 13 (6-19) |
| D7 | 17∙3 (8∙3-29∙3) | 15∙6 (7∙6-25∙2) | 14∙9 (8∙1-35∙6) | 18∙8 (8∙3-45∙2) | 17∙2 (5∙9-47∙6) | 13∙9 (6-28) | 11∙5 (5-19) |
| D28 | 22∙2 (9∙1-99) | 19∙4 (8-73) | 14∙8 (8-27∙2) | 18 (7∙7-33∙2) | 16∙3 (6∙8-37∙1) | - | - |
| D180 | 18∙7 (10-36) | 29∙9 (9-206) | 18∙8 (6-53) | 18∙8 (0-48) | 14∙9 (8-23) | - | - |
| AST, U/L |  |  |  |  |  |  |  |
| Screening | 18∙5 (11∙9-26∙7) | 18∙5 (13∙3-26) | 22∙8 (14∙9-46∙8) | 22∙3 (13∙9-51∙4) | 20∙2 (11∙7-32∙5) | 23∙5 (13-37) | 21∙8 (16-33) |
| D1 | 17∙9 (11∙4-26) | 19∙5 (12∙8-33∙2) | 20∙8 (14∙1-33) | 23∙3 (12∙1-45∙2) | 81 (10∙5-999∙9) | 19 (19-19) | 24∙9 (17-30) |
| D2/3 | 19∙5 (11∙7-31∙8) | 20 (12∙3-42∙2) | 22∙2 (10∙7-44) | 24∙1 (14∙8-52) | 31∙4 (9∙6-193) | 23∙4 (18-35) | 21∙4 (16-26) |
| D7 | 21∙7 (12∙2-42∙8) | 19∙2 (8∙5-30∙4) | 23∙1 (15-36) | 23∙3 (13∙1-44) | 17∙7 (9∙9-29∙2) | 25∙1 (18-40) | 22∙9 (16-32) |
| D28 | 22 (14∙5-33∙1) | 32∙3 (12∙3-164) | 23∙5 (16-32∙9) | 22∙7 (11∙3-38∙6) | 19 (11∙4-29∙6) | - | - |
| D180 | 22∙3 (15-36) | 22 (14-36) | 25∙9 (15-46) | 26∙2 (14-59) | 23∙5 (14-33) | - | - |
| All values are expressed in mean (IQR)  D: Time point in day(s) since vaccination | | | | | | | |
