# Supplementary material for: Safety and immunogenicity of rVSVΔG-ZEBOV-GP Ebola vaccine in adults and children in Lambaréné, Gabon: A phase I randomised trial
Source: PLoS Med. 2017 Oct 6;14(10):e1002402. doi: 10.1371/journal.pmed.1002402 (PMC5630143; doi:10.1371/journal.pmed.1002402)
Supplement: S6 Table — (DOCX) [file pmed.1002402.s010.docx]

# S6 Table. GMT, seropositivity rates and proportion of seroresponders to rVSV-ZEBOV-GP vaccine measured by whole-virion ELISA in children and adolescents

| Cohorts  2x10^7^ PFU | Time | N | GMT (95%CI) | Seropositivity (>500AEU/ml),  n (%) | Seroresponse  (>2x), n (%) | Seroresponse  (>4x), n (%) | P^†^ value  GMT | P^‡^ value  Seropositivity | P^Ω^ value | P^β^ value |
| --- | --- | --- | --- | --- | --- | --- | --- | --- | --- | --- |
|  | D0 | 15 | 597∙8 (477∙8-748∙0) | 2 (13∙3) | - | - | - | - | - | - |
| children | D28 | 20 | 2267∙1 (1343∙3-3826∙1) | 14 (70∙0) | 10 (66∙7) | 8 (53.3) | **0∙006** | **0∙01** | **<0∙001** | **<0∙001** |
|  | D56 | 20 | 3380∙9 (2282∙7-5007∙3) | 18 (90∙0) | 14 (93∙3) | 11 (73.3) | **0∙001** | **0∙001** | 0∙06 | 0.26 |
| Adolescents | D0 | 15 | 500∙0 (500∙0-500∙0) | 0 (0) | - | - | - | - | - | - |
|  | D28 | 15 | 1636∙2 (1005∙9-2661∙3) | 9 (60∙0) | 6 (54∙5) | 4 (36.4) | **0∙03** | **0∙04** | **0∙002** | **0.06** |
|  | D56 | 16 | 2137∙3 (1361∙9-3354∙3) | 13 (81∙2) | 9 (69∙2) | 4 (30.8) | **0∙006** | **0∙004** | **0∙01** | 0.49 |
| Results are expressed in geometric mean titers (GMT) of arbitrary ELISA units (AEU)/ml with 95% confidence intervals. Seropositivity is defined by GMT>500 AEU/ml. Seroresponse expressed as a >4-fold increase in titers.  D: Time point in day(s) since vaccination  †: Wilcoxon’s test for paired data. P< 0.05 indicates a statistical difference in antibody titers between day 0 and others days  ‡: McNemar test. P< 0.05 indicates a statistical difference in seropositivity rates between day 0 and others days  Ω: Fisher’s test. P< 0.05 indicates a statistical association between seropositivity and seroresponse (>2x) for each timepoint  β: Fisher’s test. P< 0.05 indicates a statistical association between seropositivity and seroresponse (>4x) for each timepoint | | | | | | | | | | |
