# Supplementary material for: Safety and immunogenicity of rVSVΔG-ZEBOV-GP Ebola vaccine in adults and children in Lambaréné, Gabon: A phase I randomised trial
Source: PLoS Med. 2017 Oct 6;14(10):e1002402. doi: 10.1371/journal.pmed.1002402 (PMC5630143; doi:10.1371/journal.pmed.1002402)
Supplement: S7 Table — (DOCX) [file pmed.1002402.s011.docx]

# S7 Table. ZEBOV antibodies per baseline status in children measured by whole-virion ELISA

|  | | | | | | | |
| --- | --- | --- | --- | --- | --- | --- | --- |
|  |  |  | **With baseline ZEBOV antibodies** | |  | **Without baseline ZEBOV antibodies** | |
| Cohorts | **Time** |  | **N** | **GMT (95%CI)** |  | **N** | **GMT (95%CI)** |
| 2x10^7^ PFU | D0 |  | 2 | 1909∙5 (683∙6-5333∙9) |  | 13 | 500∙0 (-) |
|  | D28 |  | 2 | 8373∙3 (4258∙8-16463∙0) |  | 18 | 1960∙7 (1144∙8-3358∙1) |
|  | D56 |  | 2 | 8794∙2 (4089∙4-18911∙8) |  | 18 | 3040∙2 (2032∙2-4548∙1) |
| Results are expressed in geometric mean titers (GMT) with 95% confidence intervals (95% CI). Participants expressing GMT>500 AEU/ml were considered seropositive at baseline. Values below the threshold were given arbitrary units of 500 AEU/ml.  D: Time point in day(s) since vaccination | | | | | | | |
