# Supplementary material for: Safety and immunogenicity of rVSVΔG-ZEBOV-GP Ebola vaccine in adults and children in Lambaréné, Gabon: A phase I randomised trial
Source: PLoS Med. 2017 Oct 6;14(10):e1002402. doi: 10.1371/journal.pmed.1002402 (PMC5630143; doi:10.1371/journal.pmed.1002402)
Supplement: S8 Table — (DOCX) [file pmed.1002402.s012.docx]

# S8 Table. Neutralizing antibodies to infectious ZEBOV isolate expressed in GMT, seropositivity rates and proportion of seroresponders in adults

|  | |  | | | | | | | | |  |
| --- | --- | --- | --- | --- | --- | --- | --- | --- | --- | --- | --- |
| Cohorts | **Time** | | **N** | **GMT (95%CI)** | **Seropositivity^α^**  **n (%)** | **Seroresponse**  **(>2x), n (%)** | **Seroresponse**  **(>4x), n (%)** | **P^†^ value**  **GMT** | **P^‡^ value**  **Seropositivity** | **P^Ω^ value** | **P^β^ value** |
| 3x10^5^ | D0 | | 20 | 7∙5 (5∙9-9∙5) | 7 (35) | - | - | - | - | - | - |
|  | D28 | | 20 | 19∙5 (11∙9-32∙1) | 14 (70) | 9 (45) | 9 (45) | **0∙002** | **0∙02** | **0∙01** | **0∙05** |
| 3x10^6^ | D0 | | 39 | 4∙9 (4∙6-5∙2) | 5 (12∙8) | - | - | - | - | - | - |
|  | D28 | | 39 | 13∙0 (10∙1-16∙8) | 33 (84∙6) | 25 (64.1) | 12 (30.8) | **<0∙0001** | **<0∙0001** | **<0∙0001** | 0.1 |
| 2x10^7^ | D0 | | 16 | 5∙1 (4∙6-5∙7) | 4 (25) | - | - | - | - | - | - |
|  | D28 | | 16 | 9∙5 (6∙4-14∙1) | 9 (56∙2) | 6 (37.5) | 3 (18.8) | **0∙002** | **0∙07** | **0.02** | **0.03** |
| α: A cut-off has been defined for each cohort such as GMT+SD. For 3x10^5^ (titers> 9∙5), 3x10^6^ (titers> 5∙9) and 2x10^7^(titers> 6∙1)  †: Wilcoxon’s test for paired data. P< 0.05 indicates a statistical difference in antibody titers between days 0 and others days  ‡: McNemar test. P< 0.05 indicates a statistical difference in seropositivity rates between days 0 and others days  Ω: Fisher’s test. P< 0.05 indicates a statistical association between seropositivity and seroresponse (>2x) for each timepoint  β: Fisher’s test. P< 0.05 indicates a statistical association between seropositivity and seroresponse (>4x) for each timepoint | | | | | | | | | | | |
