# Supplementary material for: Safety and immunogenicity of rVSVΔG-ZEBOV-GP Ebola vaccine in adults and children in Lambaréné, Gabon: A phase I randomised trial
Source: PLoS Med. 2017 Oct 6;14(10):e1002402. doi: 10.1371/journal.pmed.1002402 (PMC5630143; doi:10.1371/journal.pmed.1002402)
Supplement: S9 Table — (DOCX) [file pmed.1002402.s013.docx]

# S9 Table. Neutralizing antibodies to infectious ZEBOV isolate classified per baseline ZEBOV antibody status in adults

|  | | | | | | | | | |
| --- | --- | --- | --- | --- | --- | --- | --- | --- | --- |
|  |  |  | **With baseline ZEBOV antibodies** | | |  | **Without ZEBOV antibodies** | | |
| Cohorts | **Time** |  | **N** | **GMT (95%CI)** | **P^†^ value** |  | **N** | **GMT (95%CI)** | **P^†^ value** |
| 3x10^5^ PFU | D0 |  | 7 | 13∙7 (10∙3-18∙1) | - |  | 13 | 5∙4 (4∙8-6∙2) | - |
|  | D28 |  | 7 | 40 (15∙7-101∙7) | 0∙07 |  | 13 | 13∙3 (8∙2-21∙5) | **0∙008** |
| 3x10^6^ PFU | D0 |  | 5 | 7 (6∙4-7∙8) | - |  | 34 | 4∙6 (4∙4-4∙8) | - |
|  | D28 |  | 5 | 8 (5∙1-12∙5) | 0∙58 |  | 34 | 14 (10∙6-18∙5) | **<0∙0001** |
| 2x10^7^ PFU | D0 |  | 4 | 6∙7 (6∙7-6∙7) | - |  | 12 | 4∙7 (4∙3-5∙1) | - |
|  | D28 |  | 4 | 18∙2 (8∙9-37∙1) | 0∙09 |  | 12 | 7∙7 (5∙1-11∙6) | **<0∙0001** |
| Results are presented as geometric mean titers (GMT) with 95% confidence intervals. Seropositivity is defined using a cut-off for each cohort such as GMT+SD. For 3x10^5^ (titers> 9∙5), 3x10^6^ (titers> 5∙9) and 2x10^7^(titers> 6∙1). Seroresponse is defined by ≥ 4-fold rise in GMTs  D: Time point in day(s) since vaccination  †: Wilcoxon’s test for paired data∙P< 0.05 indicates a statistical difference in antibody titers between days 0 and others days | | | | | | | | | |
