# Supplementary material for: Safety and immunogenicity of rVSVΔG-ZEBOV-GP Ebola vaccine in adults and children in Lambaréné, Gabon: A phase I randomised trial
Source: PLoS Med. 2017 Oct 6;14(10):e1002402. doi: 10.1371/journal.pmed.1002402 (PMC5630143; doi:10.1371/journal.pmed.1002402)
Supplement: S10 Table — (DOCX) [file pmed.1002402.s014.docx]

# S10 Table. Neutralizing antibodies to infectious ZEBOV isolate expressed in GMT, seropositivity rates and proportion of seroresponders in children and adolescents

|  | |  | | | | | | | | |  |
| --- | --- | --- | --- | --- | --- | --- | --- | --- | --- | --- | --- |
| Cohorts  2x10^7^ PFU | **Time** | | **N** | **GMT (95%CI)** | **Seropositivity**  **(titers), n (%)** | **Seroresponse**  **(>2x), n (%)** | **Seroresponse**  **(>4x), n (%)** | **P^†^ value**  **GMT** | **P^‡^ value**  **Seropositivity** | **P^Ω^ value** | **P^β^value** |
| Children | D0 | | 15 | 4∙6 (4∙2-5) | 0 (0) | - | - | - | - | - | - |
|  | D28 | | 20 | 20∙4 (12∙9-32∙3) | 19 (95) | 12 (60) | 8 (40) | **0∙001** | **0∙004** | **0∙2** | **1** |
|  |  | |  |  |  |  |  |  |  |  |  |

| Adolescents | D0 | 11 | 4∙1 (4-4∙3) | 0 (0) | - | - | - | - | - | - |
| --- | --- | --- | --- | --- | --- | --- | --- | --- | --- | --- |
|  | D28 | 15 | 10∙4 (8-13∙6) | 12 (80) | 8 (38.1) | 4 (19) | **0∙01** | **0∙01** | **0∙006** | 1 |

| Results are presented as geometric mean titers (GMT) with 95% confidence intervals (95%CI). Seropositivity is defined using a cut-off for each cohort such as GMT+SD. for 2x10^7^(titers> 6∙1). Seroresponse is defined by a ≥ 4-fold rise in GMTs  D: Time point in day(s) since vaccination  †: Wilcoxon’s test for paired data. P< 0.05 indicates a statistical difference in antibody titers between days 0 and day28  ‡: McNemar’s test. P< 0.05 indicates a statistical difference of in seropositivity rates between day 0 and day 28  Ω: Fisher’s test. P< 0.05 indicates a statistical association between seropositivity and seroresponse (>2x) for each timepoint  β: Fisher’s test. P< 0.05 indicates a statistical association between seropositivity and seroresponse (>4x) for each timepoint |
| --- |
