# Supplementary material for: Safety and immunogenicity of rVSVΔG-ZEBOV-GP Ebola vaccine in adults and children in Lambaréné, Gabon: A phase I randomised trial
Source: PLoS Med. 2017 Oct 6;14(10):e1002402. doi: 10.1371/journal.pmed.1002402 (PMC5630143; doi:10.1371/journal.pmed.1002402)
Supplement: S11 Table — (DOCX) [file pmed.1002402.s015.docx]

# S11 Table. Neutralizing antibodies to VSV pseudovirions expressed in GMT, seropositivity rates and proportion of seroresponders to rVSV-ZEBOV vaccine measured by ZEBOV Pseudovirus neutralization 50 in children and adolescents

| Cohorts  2x10^7^ PFU | Time | N | GMT (95%CI) | Seropositivity  (>20 AEU/ml),  n (%) | Seroresponse  (>4 x), n (%) | P^†^ value  GMT | P^‡^ value  Seropositivity | P^Ω^ value |
| --- | --- | --- | --- | --- | --- | --- | --- | --- |
| Children | D0 | 15 | 19 (-) | 0 (0) | 0 (0) | - | - | - |
|  | D28 | 20 | 118.2 (56.2-248.6) | 14 (70) | 12 (60) | **0.005** | **0.004** | <0.001 |
|  | D56 | - | - | - | - | **-** | **-** | - |
| Adolescents | D0 | 15 | 19 (-) | 0 (0) | 0 (0) | - | - | - |
|  | D28 | 15 | 76.5 (36.9-158.5) | 10 (66.7) | 8 (53.3) | **0.02** | **0.02** | 0.007 |
|  | D56 | 15 | 95.7 (50.5-181.6) | 11 (73.3) | 9 (60) | **0.01** | **0.01** | 0.01 |
| Results are presented as geometric mean titers (GMT) with 95% confidence intervals. Seropositivity is defined by GMT>20. Seroresponse is defined by a ≥ 4-fold rise in GMTs  D: Time point in day(s) since vaccination  †: Wilcoxon’s test for paired data. P< 0.05 indicates a statistical difference in antibody titers between days 0 and others days  ‡: McNemar’s test. P< 0.05 indicates a statistical difference in seropositivity rates between days 0 and others days  Ω: Fisher’s test. P< 0.05 indicates a statistical association between seropositivity and seroresponse for each timepoint | | | | | | | | |
