# Supplementary material for: Safety and immunogenicity of rVSVΔG-ZEBOV-GP Ebola vaccine in adults and children in Lambaréné, Gabon: A phase I randomised trial
Source: PLoS Med. 2017 Oct 6;14(10):e1002402. doi: 10.1371/journal.pmed.1002402 (PMC5630143; doi:10.1371/journal.pmed.1002402)
Supplement: S12 Table — (DOCX) [file pmed.1002402.s016.docx]

# S12 Table. Comparison of ZEBOV and neutralizing antibodies expressed in GMT measured by whole-virion ELISA and virus neutralisation in adults, children and adolescent vaccinated with 2x10^7^PFU

|  |  |  | Adults | | | |  | | Children | | | | Adolescents | |  |
| --- | --- | --- | --- | --- | --- | --- | --- | --- | --- | --- | --- | --- | --- | --- | --- |
| Cohorts | **Time** |  | **N** | | **GMT (95%CI)** | |  | | **N** | | **GMT (95%CI)** | | **N** | **GMT (95%CI)** | **P*** |
| whole-virion ELISA | | | | | | | | | | | | | | |  |
| 2x10^7^ PFU | D0 |  | 16 | | 1625∙2 (878∙8-3005∙5) | |  | | 15 | | 597∙8 (477∙8-748∙0) | | 15 | 500∙0 (-) | **<0.001** |
|  | D28 |  | 16 | | 3958∙0 (2248∙5-6967∙4) | |  | | 20 | | 2267∙1 (1343∙3-3826∙1) | | 15 | 1636∙2 (1005∙9-2661∙3) | **0.05** |
|  | D56 |  | 13 | | 4402∙0 (2887∙6-6710∙6) | |  | | 20 | | 3380∙9 (2282∙7-5007∙3) | | 16 | 2137∙3 (1361∙9-3354∙3) | 0.1 |
| Neutralisation | | | | | | | | | | | | | | | |
| 2x10^7^ PFU | D0 |  | | 16 | | 5∙1 (4∙6-5∙7) | |  | | 15 | | 4∙6 (4∙2-5) | 11 | 4∙1 (4-4∙3) | **0.01** |
|  | D28 |  | | 16 | | 9∙5 (6∙4-14∙1) | |  | | 20 | | 20∙4 (12∙9-32∙3) | 15 | 10∙4 (8-13∙6) | **0.04** |
| Results are presented as geometric mean titers (GMT) with 95% confidence intervals.  D: Time point in day(s) since vaccination  *: Kruskal-Wallis test. P<0.05 indicates a significant statistical difference in antibody titers between groups (adults, children and adolescent) at each time point. | | | | | | | | | | | | | | | |
