# Supplementary material for: Safety and immunogenicity of rVSVΔG-ZEBOV-GP Ebola vaccine in adults and children in Lambaréné, Gabon: A phase I randomised trial
Source: PLoS Med. 2017 Oct 6;14(10):e1002402. doi: 10.1371/journal.pmed.1002402 (PMC5630143; doi:10.1371/journal.pmed.1002402)
Supplement: S13 Table — (DOCX) [file pmed.1002402.s017.docx]

# S13 Table. rVSV RNA shedding, proportion of adolescents and children with detectable and quantifiable viral RNA

| **Specimen/**  **Timepoint** | ***Adolescent*** | | | **Children** | | |
| --- | --- | --- | --- | --- | --- | --- |
|  | **Detectable, % (n/N)** | **Quantifiable, % (n/N)** | **Viral load**  **median copies/ml, (IQR)** | **Detectable, % (n/N)** | **Quantifiable, % (n/N)** | **Viral load**  **median Copies/ml, (IQR)** |
| **Saliva** |  |  |  |  |  |  |
| D2 | 41 (8/19) | 11 (2/19) | 20∙64 (00-47∙94) | 30 (6/20) | 10 (1/20) | 13∙71 (0-40∙61) |
| D7 | 78 (14/18) | 67 (12/18) | 460∙7 (30∙16-5502) | 35 (7/20) | 30 (6/20) | 0 (0-1776) |
| **Urine** |  |  |  |  |  |  |
| D2 | 0 (0/10) | 0 (0/10) | 0 (0-0) | 22 (2/9) | 0 (0/9) | 10∙4 (0-29∙54) |
| D7 | 10 (1/10) | 10 (1/10)) | 0 (0-0) | 11 (1/9) | 11 (1/9) | 0 (0-3∙62) |

**S13 Table. Viral load in urine and saliva for children and adolescents. rVSV RNA copy numbers in urine and saliva presented in copies/ml from day 2 and 7 post injection in adolescents and children vaccinated with 2x10^7^ PFU. Positive samples defined as detectable RNA copies above 30 copies/ml. Samples between 30-100 copies/ml RNA were detectable but not quantifiable. Quantifiable samples defined as RNA copies >100 copies/ml.**
