# Supplementary material for: Safety and immunogenicity of rVSVΔG-ZEBOV-GP Ebola vaccine in adults and children in Lambaréné, Gabon: A phase I randomised trial
Source: PLoS Med. 2017 Oct 6;14(10):e1002402. doi: 10.1371/journal.pmed.1002402 (PMC5630143; doi:10.1371/journal.pmed.1002402)
Supplement: S14 Table — (DOCX) [file pmed.1002402.s018.docx]

# S14 Table. Viraemia in participants with baseline ZEBOV antibodies

|  | Adults | | | | | | | | | | Children | | Adolescents | |  |
| --- | --- | --- | --- | --- | --- | --- | --- | --- | --- | --- | --- | --- | --- | --- | --- |
|  | **3x10^3^ PFU** | | **3x10^4^ PFU** | | **3x10^5^ PFU** | | **3x10^6^ PFU** | | **2x10^7^ PFU** | | **2x10^7^ PFU** | | **2x10^7^ PFU** | | **P*** |
| Time | **N** |  | **N** |  | **N** |  | **N** |  | **N** |  | **N** |  | **N** |  |  |
|  |  |  |  |  |  |  |  |  |  |  |  |  |  |  |  |
| D0 | 5 | 0(-) | 1 | 0 (0-0) | 1 | 0 (0-0) | 9 | 0 (0-0) | 8 | 0 (0-0) | 2 | 0 (0-0) |  | - | - |
| D1 | 6 | 0 (-) | 2 | 0 (0-0) | 1 | 61∙5 (61∙5-61∙5) | 8 | 177∙8 (111∙4-242∙4) | 8 | 328∙4 (311∙3-372∙8) | 0 | - |  | - | - |
| D2 | 5 | 0 (-) | 1 | 1∙4 (1∙4-1∙4) | 0 | - | 9 | 893∙6 (759∙3-1314∙1) | 8 | 627∙3 (453∙4-914∙8) | 2 | 2968∙5 (1672∙8-4264∙2) |  | - | 1 |
| D7 | 5 | 0 (-) | 1 | 10∙5 (10∙5-10∙5) | 1 | 0 (0-0) | 8 | 0 (0-8∙7) | 8 | 10∙2 (0∙5-31∙7) | 1 | 137∙6 (137∙6-137∙6) |  | - | 0∙1 |
| Viraemia expressed as median (IQR) in adults, adolescents and children.  D: Time point in day (s) since vaccination  *: Kruskal-Wallis test. P<0.05 indicates a statistical significant difference in median viraemia between adults, children and adolescent receiving 2x10^7^ PFU at each time point. | | | | | | | | | | | | | | | |
