# Supplementary material for: Safety and immunogenicity of rVSVΔG-ZEBOV-GP Ebola vaccine in adults and children in Lambaréné, Gabon: A phase I randomised trial
Source: PLoS Med. 2017 Oct 6;14(10):e1002402. doi: 10.1371/journal.pmed.1002402 (PMC5630143; doi:10.1371/journal.pmed.1002402)
Supplement: S15 Table — (DOCX) [file pmed.1002402.s019.docx]

# S15 Table. Viraemia in participants without baseline ZEBOV antibodies

|  | Adults | | | | | | | | | | Children | | Adolescents | |  |
| --- | --- | --- | --- | --- | --- | --- | --- | --- | --- | --- | --- | --- | --- | --- | --- |
|  | **3x10^3^ PFU** | | **3x10^4^ PFU** | | **3x10^5^ PFU** | | **3x10^6^ PFU** | | **2x10^7^ PFU** | | **2x10^7^ PFU** | | **2x10^7^ PFU** | | **P*** |
| Time | **N** |  | **N** |  | **N** |  | **N** |  | **N** |  | **N** |  | **N** |  |  |
| D0 | 5 | 0 (0-0) | 5 | 0 (0-0) | 18 | 0 (0-0) | 26 | 0 (0-0) | 7 | 0 (0-0) | 18 | 0 (0-0) | 20 | 0 (0-0) | 0∙06 |
| D1 | 6 | 0 (0-0) | 6 | 0 (0-0) | 17 | 0 (0-12) | 25 | 286∙5 (151∙1-703∙8) | 7 | 478∙1 (278∙7-1065∙7) | 4 | 731 (507∙1-2141∙8) | 19 | 655 (411∙7-912∙2) | 0∙9 |
| D2 | 5 | 0 (0-0) | 5 | 0 (0-15∙7) | 19 | 4 (0-29∙7) | 26 | 750 (377∙1-1253∙2) | 7 | 326∙2 (265∙3-877∙5) | 18 | 1109∙2 (724∙2-1845∙4) | 19 | 1591∙7 (1018∙8-2703∙5) | **0∙008** |
| D7 | 5 | 0 (0-0) | 5 | 3∙7 (1∙7-3∙9) | 11 | 2∙1 (0-6∙5) | 24 | 7∙9 (1∙5-40∙4) | 7 | 1∙7 (0-16∙5) | 18 | 0 (0-13∙6) | 17 | 0 (0-0∙7) | 0∙5 |
| Viraemia expressed as median (IQR)  D: Time point in day(s) since vaccination.  *: Kruskal-Wallis test. P<0.05 indicates a statistical significant difference in median viraemia between adults, children and adolescent at each time point. | | | | | | | | | | | | | | | |
