# Supplementary material for: Safety and immunogenicity of rVSVΔG-ZEBOV-GP Ebola vaccine in adults and children in Lambaréné, Gabon: A phase I randomised trial
Source: PLoS Med. 2017 Oct 6;14(10):e1002402. doi: 10.1371/journal.pmed.1002402 (PMC5630143; doi:10.1371/journal.pmed.1002402)
Supplement: S1 Text — (PDF) [file pmed.1002402.s020.pdf]

**Study Reference:** LA- BPSC1001-01
**Clinical Trial Protocol Version:** amendment 3
**Date:** 05 Feb 2015

# A Phase 1, Randomized, Open-Label, Dose-Escalation Study to Evaluate the Safety and Immunogenicity of the BPSC1001 (VSVΔG-ZEBOV) Ebola Virus Vaccine Candidate in Healthy Adult and Children Volunteers in Lambaréné, Gabon.

**Trial Sponsor:** Universitätsklinikum Tübingen (UKT)

.


**Principal Investigator:** Selidji AGNANDJI

**Coordinating Investigator:** Peter G. KREMSNER
**Scientific Adviser**: Sanjeev KRISHNA

**Modification history**

| Version | Date | Changes | Authors |
|---|---|---|---|
| Draft 1 | 9Sep 2014 | | Selidji T Agnandji /Sanjeev Krishna |
| Draft 2 | 19 Sep 2014 | | Selidji T Agnandji /Sanjeev Krishna/Martin P Grobusch /Peter G Kremsner |
| Final | 13 Oct 2014 | | Selidji T Agnandji /Sanjeev Krishna / Martin P Grobusch /Peter G Kremsner |
| Amendment 1 | 27 Oct 2014 | Changed holding rules<br>Added detail in various sections as requested by ethics committees | Selidji T Agnandji /Sanjeev Krishna / Martin P Grobusch /Peter G Kremsner |
| Amendment 2 | 30 Dec 2014 | Added evaluation of low doses<br>Added antibody isolation<br>Added information about community awareness<br>Clarified sample storage specifics<br>Added children<br>Added financing institutions | Selidji T Agnandji /Sanjeev Krishna / Martin P Grobusch /Peter G Kremsner |
| Amendment 3 | 05 Feb 2015 | Specification of dose for adolescents and children<br>Continuation of vaccination into cohort 3 | Selidji T Agnandji /Sanjeev Krishna / Martin P Grobusch /Peter G Kremsner |

**A Phase 1, Randomized, Open-Label, Dose-Escalation Study to Evaluate the Safety and Immunogenicity of the BPSC1001 (VSVΔG-ZEBOV) Ebola Virus Vaccine Candidate in Healthy Adult and Children Volunteers in Lambaréné, Gabon.**

Study code: LA- BPSC1001-01

| | |
|---|---|
| **Principal Investigator** | Dr. Selidji T AGNANDJI<br>Centre de Recherches Médicales de Lambaréné<br>Hôpital Albert Schweitzer<br>BP 118 Lambaréné, Gabon<br><br>Tel: +241 07353114<br>Email: agnandjis@lambarene.org |
| **Coordinating Investigator and sponsor`s delegated person**<br><br><br><br><br><br><br><br>**Scientific Adviser** | Prof. Dr. Peter Gottfried KREMSNER<br>Eberhard Karls Universität Tübingen<br>Institut für Tropenmedizin<br>Wilhelmstraße 27<br>D-72074 Tübingen, Germany<br>Tel: +49 7071 2987179<br>Email: peter.kremsner@uni-tuebingen.de<br><br>Prof Sanjeev KRISHNA, FRCP, ScD, FMedSci<br>Institute of Infection and Immunity<br>St. George's University of London<br>Cranmer Terrace<br>UK-London SW17 ORE<br>Tel: 020 8725 5836 (direct)<br>Mobile: +447931901724<br>Email: s.krishna@sgul.ac.uk |
| **Investigators** | Jose F Fernandes, Lead clinician, CERMEL<br>Anita Kabwende, Investigator, CERMEL<br>Aurore Hounkpatin. Bouyoukou, Investigator, study pharmacist, CERMEL<br>Martin. P. Grobusch, Investigator, Medical University Amsterdam, CERMEL<br>Michael Ramharter, Investigator, Medical University Vienna, CERMEL<br>Dieudonne Nkoghe, Investigator, CIRMF<br>Fabrice Kassa Kassa, Investigator, CIRMF |
| **Study coordination and management** | Bache Emmanuel Bache, CERMEL<br>Minsoko Pamela, CERMEL |
| **Sponsor** | University Hospital Tuebingen (UKT), |

| | |
|---|---|
| | represented by<br>Medical Director, Prof. Dr. med. M. Bamberg,<br>Director of Administration, G. Sonntag,<br>Geissweg 3<br>72076 Tuebingen |
| **Trial site** | Centre de Recherches Médicales de Lambaréné<br>(CERMEL), BP 118, Lambaréné, Gabon |
| **Local Safety Monitor** | Centre de Recherches Médicales de Lambaréné (CERMEL), BP 118, Lambaréné, Gabon<br>Tel: 07644040<br>Email: aadegnika@yahoo.fr |
| **Statistician** | Benjamin Gottlieb Mordmüller<br>Eberhard Karls Universität Tübingen<br>Institut für Tropenmedizin<br>Wilhelmstraße 27<br>D-72074 Tübingen, Germany<br>Tel: +49 7071 2982187<br>Email: benjamin.mordmueller@uni-tuebingen.de |
| **Pharmacovigilance** | Ayola Akim Adegnika<br>Centre de Recherches Médicales de Lambaréné<br>BP 118 Lambaréné, Gabon<br>Tel: +241 07989191<br>email: aadegnika@yahoo.fr |
| **Central Laboratory** | Marguerite Massinga Loembe<br>Centre de Recherches Médicales de Lambaréné<br>BP 118 Lambaréné, Gabon<br>Tel: +241 07989191<br>Email: mmassingaloembe@lambarene.org |
| **Data Safety Monitoring Board** | Represented by :<br>Prof. Markus Mueller (chair)<br>Medical University Vienna, Austria<br>Email : markus.mueller@meduniwien.ac.at |

## Signature Page

The present trial protocol was subject to critical review and has been approved in the present version by the persons undersigned.

**Sponsor**: The University Hospital Tubingen is sponsor for the purpose of § 4 (24) German Drug Law with complementary regulations. The internal responsibility to comply with the obligations of the sponsor in terms of these regulations stays with **Peter Gottfried Kremsner**

Date: __________ Signature of the delegated Sponsor:

_______________________________

Name (block letters):
Function: Delegated sponsor and person in charge to meet the obligations of the sponsor

Date: __________ Signature:

_______________________________

Name (block letters):
Function: Principal Investigator

Date: __________ Signature:

_______________________________

Name (block letters):
Function: Biometrician

Date: __________ Signature:

_______________________________

Name (block letters):
Function: Scientific Adviser

Date: __________ Signature:

_______________________________

Name (block letters):
Function: Co-Author/Project Manager

**Investigator Agreement**

"I have read this protocol and agree to abide by all provisions set forth therein. I agree to comply with the principles of the International Conference on Harmonisation Tripartite Guideline on Good Clinical Practice."

| Signature | Date (dd.mm.yyyy) |
|---|---|
| | |
| | |

**Confidentiality Statement**

This document contains confidential information that must not be disclosed to anyone other than the trial Sponsor, the Investigator Team, and members of the Institutional Review Board. This information cannot be used for any purpose other than the evaluation or conduct of the clinical investigation without the prior written consent of the principal investigator.

# Table of contents


**Signature Page** .......... **5**

**1. Synopsis** .......... **9**

**2. Abbreviations** .......... **17**

**3. Background and Rationale** .......... **18**

*3.1. Ebola Virus outbreaks* .......... *18*

*3.2. Pathogenesis of Ebola Virus, Host immune responses and Ebola Virus Disease severity* .......... *19*

*3.3. Vaccine against Ebola Virus* .......... *20*

*3.4. Rationale of current project* .......... *21*

**4. Trial Design** .......... **21**

*4.1. Overview* .......... *21*

*4.2. Objectives* .......... *22*

*4.3. Endpoints* .......... *23*

*4.4. Investigational Products* .......... *25*

*4.5. Participation and study Duration* .......... *26*

*4.6. Dose-adjustment Criteria* .......... *26*

**5. Selection and withdrawal of trial volunteers** .......... **27**

*5.1. Volunteer recruitment* .......... *27*

*5.2. Informed Consent* .......... *28*

*5.3. Inclusion Criteria* .......... *29*

*5.4. Exclusion Criteria* .......... *29*

*5.5. Withdrawal of Volunteers* .......... *30*

*5.6. Pregnancy* .......... *31*

*5.7. Potential risks for volunteers* .......... *31*

*5.8. Potential benefits for volunteers* .......... *32*

**6. Study procedures** .......... **32**

*6.1. Time points* .......... *32*

*6.2. Screening Visit* .......... *32*

*6.3. Study Enrollment/vaccination (Visit 2, Day 0)* .......... *33*

*6.4. Follow-Up Visits: visit 3 to visit 10* .......... *34*

*6.5. Safety assessment* .......... *34*

Unanticipated Problems Involving Risks to Subjects Or Others .......... 37

*6.6. Immunogenicity Assessment* .......... *40*

**7. Statistics** .......... **44**

**8. Quality control and quality assurance procedures** .......... **44**

*8.1. Investigator procedures* .......... *44*

*8.2. Monitoring* .......... *44*

*8.3. Modification to protocol* .......... *44*

*8.4. Protocol deviation* .......... *45*

*8.5. Audit and inspection* .......... *45*

*8.6. Trial Progress* .......... *45*

**9. Ethics** .......... **45**

*9.1. Declaration of Helsinki* .......... *45*

9.2. *ICH Guidelines for Good Clinical Practice* ... 45
9.3. *Informed Consent* ... 45
9.4. *Research Ethics Committee* ... 45
9.5. *Volunteer Confidentiality* ... 46
9.6. *Volunteer consent withdrawal* ... 46

**10. Data handling and record keeping ... 46**
10.1. *Data Handling* ... 46
10.2. *Record Keeping* ... 46
10.3. *Sample storage* ... 46
10.4. *Source Data and Case Report Forms (CRFs)* ... 46
10.5. *Data Protection* ... 47
10.6. *Sample Shipments* ... 47

**11. Financing and insurance ... 47**
11.1. *Financing* ... 47
11.2. *Insurance* ... 47
11.3. *Compensation of volunteers* ... 48
11.4. *Treatment costs* ... 48

**12. Community awareness and engagement ... 48**

**13. References ... 50**

**14. Annexes ... 51**

# 1. Synopsis

| | |
|---|---|
| **Title** | **A Phase 1, Randomized, Open-Label, Dose-Escalation Study to Evaluate the Safety and Immunogenicity of the BPSC1001 (VSV∆G-ZEBOV) Ebola Virus Vaccine Candidate in Healthy Adult Volunteers in Lambaréné, Gabon.** |
| **Study ID** | **LA-BPSC1001-01** |
| **Trial Centre** | Centre de Recherches Médicales de Lambaréné, BP 118, Lambaréné, Gabon |
| **Study Design** | Single center, open-label, randomized trial |
| **Population** | Healthy adults aged 18 to 50 years, adolescents aged 13 to 17 years and children aged 6 to 12 years |
| **Rationale for Study Design** | **Rationale**<br>Ebola Virus Diseases (EVD) are historically sporadic outbreaks that occur in remote areas of tropical forest mainly in Central Africa. Although EVD are associated with a very high case fatality rate (>50%), Ebola virus transmissability has so far been low and outbreaks have often been rapidly contained in their area of occurrence. Since January 2014, an outbreak has occurred in West Africa, which in contrast with previous outbreaks, has become established in Guinea, Liberia and Sierra Leone, so it may be considered an epidemic. A synchronous Ebola outbreak has recently emerged in the Democratic Republic of Congo. The current outbreak is a major global health and security concern as it has spread in an unprecedented way to capital cities and across countries with Nigeria and Senegal reporting imported cases that in Nigeria have led to autochthonous transmission. The intolerable burden on weak health systems in the 3 most affected countries is devastating. The epidemic is out of control. In response, WHO is coordinating a global Response Road Map with the goal to stop Ebola transmission globally within 6-9 months. Among the major means to reach this objective, prompt clinical evaluation of safety and immunogenicity of existing candidate vaccines is a top priority. To address this global emergency, and this overwhelming priority, this study aims to provide first clinical data in Africa on a VSV∆G-ZEBOV candidate vaccine by end of 2014 and in early 2015.<br>**Study design**<br>The study is designed to establish safety, tolerability and immunogenicity of VSV∆G-ZEBOV, an Ebola Virus Vaccine candidate investigated in five different dosages in sub-Saharan African adult |

| | |
|---|---|
| | populations living in Lambaréné, Gabon. One hundred and sixty one adult volunteers will be randomized into five dosage groups, and a further 40 school aged and adolescent volunteers will be vaccinated with a $2x10^7$ pfu dose.. There will be seven cohorts. Vaccination of subjects in each cohort will proceed in a staggered manner (1 to 10 participants per day will be vaccinated with a waiting time of 1 hour for each participant after vaccination). This study is part of the VEBCON consortium that is aiming to generate harmonized data for this vaccine candidate to allow optimized rapid decisions on dosage, safety, and immunogenicity as well as regulatory approval. The VEBCON consortium includes partners from Africa, EU, WHO. Partner from USA may join VEBCON or work closely. There will be in parallel to this study, similar Phase I trials in USA, Switzerland, Germany and Kenya.<br><br>**VSVΔG-ZEBOV dose selection**<br>So far, VSVΔG-ZEBOV vaccine was given to one German laboratory worker at 5 x $10^7$ pfu dosage in Ebola virus post exposure conditions. S/he experienced transient fever and very low-level VSV viremia for 2 days. Doses selected to be tested in this trial are: $3x10^3$ pfu, $3x10^4$ pfu, $3x10^5$ pfu, $3x10^6$, pfu, $2x10^7$ pfu in Gabon. The reference dosage of $5x10^7$ has been administered in post-exposure conditions. Lower doses therefore may be immunogenic enough, better tolerated and safer for pre-exposure purposes. Thus within the VEBCON consortium and in US, a large range of doses: $10^3$ to $10^8$ will be tested to support as best as possible discussion on appropriate dosage for pre-exposure and potentially post exposure purposes.. |
| **Groups** | **Groups**<br>1) Volunteers aged between 18 and 50 years inclusive, who will receive $3x10^5$ pfu of VSVΔG-ZEBOV vaccine IM<br>2) Volunteers aged between 18 and 50 years inclusive, who will receive $3x10^6$ pfu of VSVΔG-ZEBOV vaccine IM<br>3) Volunteers aged between 18 and 50 years inclusive, who will receive 2 $x10^7$ pfu of VSVΔG-ZEBOV vaccine IM – discontinued<br>4) Volunteers aged between 18 and 50 years inclusive, who will receive $3x10^3$ pfu of VSVΔG-ZEBOV vaccine IM<br>5) Volunteers aged between 18 and 50 years inclusive, who will receive $3x10^4$ pfu of VSVΔG-ZEBOV vaccine IM<br>6) Volunteers aged between 13 and 17 years inclusive, who will receive VSVΔG-ZEBOV vaccine IM<br>7) Volunteers aged between 6 and 12 years inclusive, who will receive VSVΔG-ZEBOV vaccine IM. |

| | |
|---|---|
| | **Cohorts of participants**<br>The study volunteers will be vaccinated through seven cohorts (1, 2, 3 4, 5, 6 and 7) in a staggered manner. On Day 1, the 1st volunteer of cohort 1 will be vaccinated, followed by 2nd and 3rd volunteers on Day 3. A group of 5 participants on day 5. The same plan will apply for cohorts 2 and 3, and the same protocol followed for cohorts 4-7. Following the first 10 participants, volunteers will be vaccinated in groups of 5-10 per day irrespective of the cohort. |
| **Breakpoints** | The study is terminated:<br>1) When the last participant attends his/her last visit<br>2) Upon agreement of PI, sponsor, data safety monitoring board (DSMB) and VEBCON consortium after occurrence/re-occurrence of stopping rules or any other reasons which lead to an agreement between the VEBCON partners to stop terminate the trial. |
| **Stopping rules** | The study will be placed on safety hold in case of occurrence of a vaccine related SAE |
| **Sample Size** | – A total of 201 volunteers will be enrolled in this trial<br>• 20 Volunteers aged between 18 and 50 years inclusive, who will receive $3x10^3$ pfu of VSVΔG-ZEBOV vaccine IM<br>• 20 Volunteers aged between 18 and 50 years inclusive, who will receive $3x10^4$ pfu of VSVΔG-ZEBOV vaccine IM<br>• 20 Volunteers aged between 18 and 50 years inclusive, who will receive $3x10^5$ pfu of VSVΔG-ZEBOV vaccine IM<br>• 39 Volunteers aged between 18 and 50 years inclusive, who will receive $3x10^6$ pfu of VSVΔG-ZEBOV vaccine IM<br>• 62 Volunteer aged between 18 and 50 years inclusive, who will receive 2 $x10^7$ pfu of VSVΔG-ZEBOV vaccine IM<br>• 20 Volunteers aged between 13 and 17 years inclusive, who will receive 2 $x10^7$ pfu of VSVΔG-ZEBOV vaccine IM<br>• 20 Volunteers aged between 6 and 12 years inclusive, who will receive 2 $x10^7$ pfu of VSVΔG-ZEBOV vaccine IM. |
| **Follow-up duration** | **Daily visits for tolerability**:<br>Day: 1, 2 and 7 are at clinic and the remainder at home<br>**Safety and immunogenicity key visits**:<br>Day 14, Day 28, Day 56, Day 84, and Day 180 post vaccination |
| **Planned Trial** | November 2014 to December 2015 for recruitment and interim follow |

| | |
|---|---|
| **conduct Period** | up, to generate acute safety data. Longer-term follow up will be for 180 days. |
| **Primary Objectives** | To evaluate the safety and tolerability of five different doses of VSVΔG-ZEBOV vaccine in adults living in Lambaréné, Gabon and to evaluate the safety and tolerability of $2x10^{7}$ pfu dose of VSVΔG-ZEBOV vaccine in children and adolescents living in Lambaréné, Gabon. |
| **Secondary Objectives** | • To evaluate the ZEBOV-specific antibody responses induced by VSVΔG-ZEBOV vaccine<br>• To evaluate vaccine viremia and excretion after administration of VSVΔG-ZEBOV vaccine<br>• To relate status of pre-vaccination asymptomatic exposure to filoviruses of volunteers to ZEBOV-specific antibody responses |
| **Exploratory Objectives** | • To evaluate immune response in the context of HLA allele expression<br>• To assay Ebola protein reactivity by Western blot any filovirus seropositives before vaccination<br>• To evaluate B-cell repertoire after vaccination<br>• To evaluate T-cell repertoire after vaccination<br>• To perform T-cell phenotyping after vaccination<br>• Obtain and preserve specimens to support assays to dissect the mechanism of the post-exposure protection observed in the NHP model<br>• To evaluate memory B- and T-cell responses up to 6 months after vaccination |
| **VSVΔG-ZEBOV vaccine** | VSVΔG-ZEBOV, a recombinant vesicular stomatitis virus (VSV) expressing the envelope glycoprotein of Ebola Virus Zaire |
| **Form**<br><br>**Route of Administration**<br>**Dose per administration** | The vaccine is supplied individually packaged in sterile 1.0 mL vials. Each vial contains vaccine virus frozen in a mixture of USP water for injection, human serum albumin (25%, USP) and 10 mM Tris, ph 7.2. After thawing, the content of the vials is a clear liquid.<br><br>Intramuscular injection into the deltoid region of the upper extremity, using a 3mL syringe with a 1 to 1.5 inch #25-gauge sterile needle.<br>The vaccine concentrations in the vials are formulated as: $1 \times 10^{8}$ pfu/ml (stock).<br>Cohort 1: $3x10^{5}$pfu each dose (IM)<br>Cohort 2: $3x10^{6}$ pfu each dose (IM) |

| | |
|---|---|
| | Cohort 3: $2x10^7$ pfu each dose (IM)<br>Cohort 4: $3x10^3$pfu each dose (IM)<br>Cohort 5: $3x10^4$pfu each dose (IM)<br>Cohort 6: $2x10^7$ pfu each dose (IM)<br>Cohort 7: $2x10^7$ pfu each dose (IM) |
| **Endpoints** | Key endpoints:<br>Safety and tolerability:<br>The frequency, and severity of adverse events (AEs) and serious adverse events (SAEs) associated with administration of BPSC1001 at 5 different doses.<br>• Solicited AEs occurring from the time of each injection through 14 days following the procedure, facilitated with the use of a memory aid<br>• Unsolicited AEs from the time of injection through 28 days following injection<br>• SAEs from the time of the first injection through the final study visit for subject (day 180)<br><br>Concentration of rVSV in blood, urine, or saliva as detected by RT-PCR at day 1, 2 and 7.<br>**Immunogenicity:**<br>Concentration of ZEBOV-specific antibody responses. The ZEBOV-specific antibody responses will be assayed in an approved laboratory so that results between trial sites can be compared reliably at days 7, 28, 84 and 180 post-vaccination).<br>Number of ZEBOV-specific memory B-cells and CD4/CD8-positive, ZEBOV-specific T-cells at days 7, 28, 84 and 180 |
| **Statistical Methods** | Every effort will be made to ensure that data collection will be harmonized between sites so that there is comparability and the ability to combine datasets as part of VEBCON network. Demographic variables and dose will be key associated variables. Fisher's exact test will be used to compare seroconversion rates and proportion of subjects who report AEs in each experimental group. Repeated measures analysis will be used to compare antibody titre between groups, with allowance for anticipated baseline seropositivity in some volunteers. A detailed statistical analysis plan will be developed during the course of this trial |
| **Inclusion Criteria** | The volunteer must satisfy all the following criteria to be eligible for the study: |

| | |
|---|---|
| | • Healthy adult male or non-pregnant, non-lactating female, ages 18 to 50 (inclusive) at the time of screening, for cohorts 1-5<br>• Healthy male or non-pregnant female child or adolescent, ages 6-17 (inclusive) at the time of screening, for cohorts 6 and 7<br>• Have provided written informed consent prior to screening procedures (or had their guardian provide written informed consent prior to screening procedures, for cohorts 6 and 7, and provided written assent if aged 11 and above)<br>• Free of clinically significant health problems, as determined by pertinent medical history and clinical examination prior to entry into the study<br>• Available, able, and willing to participate for all study visits and procedures<br>• Negative pregnancy-test for female volunteers<br>• Females, of childbearing potential, who are willing to use of the study effective methods of contraception during 30 days after vaccination.<br>  o A woman is considered of childbearing potential unless post-menopausal (≥ one year without menses) or surgically sterilized (tubal ligation, bilateral oophorectomy, or hysterectomy)<br>  o Effective contraception is defined as a contraceptive method with failure rate of less than 1% per year when used consistently and correctly and when applicable, in accordance with the product label for example:<br>    ▪ Oral contraceptives, either combined or progestogen alone,<br>    ▪ injectable progestogen,<br>    ▪ implants of etenogestrel or levonorgestrel,<br>    ▪ oestrogenic vaginal ring<br>    ▪ percutaneous contraceptive patches,<br>    ▪ intrauterine device or intrauterine system,<br>    ▪ male partner sterilisation at least 6months prior to the female subject's entry into the study, and the relationship is monogamous,<br>    ▪ male condom combined with a vaginal spermicide (foam, gel, film, cream or suppository).<br>    ▪ male condom combined with a female diaphragm, either with or without a vaginal spermicide (foam, gel, film, cream, or suppository)<br>• Be willing to minimize blood and body fluid exposure of others for 5 days after vaccination |

| | |
|---|---|
| | o Be advised to use of effective barrier prophylaxis, such as latex condoms, during penetrative sexual intercourse 30 days after vaccination<br>o Avoiding the sharing of needles, razors, or toothbrushes<br>o Avoiding open-mouth kissing |
| **Exclusion Criteria** | The volunteer will not enter the study if any of the following apply:<br>• History of severe local or systemic reactions to any vaccination or a history of severe allergic reactions<br>• Known allergy to the components of the BPSC1001 vaccine product<br>• Ongoing participation in another clinical trial<br>• Receipt of licensed vaccines within 14 days of planned study immunization (30 days for live vaccines)<br>• Acute or chronic, clinically significant psychiatric, hematologic, pulmonary, cardiovascular, or hepatic or renal functional abnormality as determined by the investigator based on medical history, physical exam, and/or laboratory screening test (including sickle cell anemia)<br>• Viral Serologies (HBsAg, anti-HCV Ab, anti-HIV)<br>• Any confirmed or suspected immunosuppressive or immunodeficient condition, cytotoxic therapy in the previous 5 years, and/or diabetes requiring medication<br>• Any chronic or active neurologic disorder, including migraines, seizures, and epilepsy, excluding a single febrile seizure as a child<br>• Have a known history of Guillain-Barré Syndrome<br>• Have an active malignancy or history of metastatic or hematologic malignancy<br>• Suspected or known alcohol and/or illicit drug abuse within the past 5 years<br>• Moderate or severe illness and/or fever $>38°C$ within 1 week prior to vaccination<br>• Pregnant or lactating female, or female who intends to become pregnant 30 days after vaccination<br>• Administration of immunoglobulins and/or any blood products within the 120 days preceding study entry or planned administration during the study period<br>• History of blood donation within 30 days of enrollment or plans to donate within the study period<br>• Administration of chronic (defined as more than 14 days) immunosuppressants or other immune-modifying drugs within 6 months of study entry |

| | |
|---|---|
| | o For corticosteroids, this will mean prednisone, or equivalent, greater than or equal to 0.5 mg/kg/day<br>o Intranasal and topical steroids are allowed<br>• Have an immunocompromised household member<br>• Any other significant finding that in the opinion of the investigator would increase the risk of the individual having an adverse outcome from participating in this study |

## 2. Abbreviations

| | |
|---|---|
| AE | Adverse Event |
| BSC | Biological Safety Cabinet |
| CERMEL | Centre de Recherches Médicales de Lambaréné |
| CBC | Complete Blood Count |
| DSMB | Data Safety Monitoring Board |
| EBOV | Ebola Virus |
| EVD | Ebola Virus Diseases |
| EC | Ethics Committee |
| FDA | US Food and Drug Administration |
| GCP | Good Clinical Practice |
| GMP | Good Manufacturing Practice |
| HBsAg | Hepatitis Surface Antigen |
| HCV | Hepatitis C Virus |
| HIV | Human Immunodeficiency Virus |
| HAS | Human Serum Albumin |
| LSM | Local Safety Monitor |
| NAI | Naturally Acquired Immunity |
| NHP | Non-Human Primates |
| PBS | Phosphate Buffered Saline |
| PCR | Polymerase Chain Reaction |
| PI | Principal Investigator |
| PMR | Parasite Multiplication Rate |
| RUNMC | Radboud University Nijmegen Medical Center |
| RT-PCR | Real Time Polymerase Chain Reaction |
| rVSV | Recombinant Vesicular Stomatitis Virus |
| SAE | Serious Adverse Event |
| SOP | Standard Operating Procedure |
| SmPC | Summary of Product Characteristics |
| SUSAR | Suspected Unexpected Serious Adverse Reaction |
| UKT | Universitätsklinikum Tübingen |
| VEBCON | Vaccine for Ebola Consortium |
| VSV | Vesicular Stomatitis Virus |
| WHO | World Health Organization |

# 3. Background and Rationale

## 3.1. Ebola Virus outbreaks

Ebola virus (EBOV) is a member of the family of Filoviridae together with and Marburg virus (MARV). So far, 5 different species of EBOV have been identified to be responsible of EVD in humans including *Zaire ebolavirus* (ZEBOV), *Sudan ebolavirus* (SEBOV), *Côte d'Ivoire*

*ebolavirus* (CIEBOV) and *Reston ebolavirus* (REBOV) and the recently discovered *Bundibugyo ebolavirus* (BEBOV)[1,2]. Among these species, ZEBOV is the most frequent and pathogenic (up to 90% case fatality rate), followed by SEBOV (approximately 50% case fatality rate) and BEBOV (approximately 40% case fatality rate)[2]. Table 1 shows a summary of the Ebola outbreaks occurrence. Ebola Virus Diseases (EVD) are historically sporadic outbreaks, which occur in remote areas of tropical forest mainly in Central Africa. Although EVD are associated to a high case fatality rate, Ebola virus transmission was so far low and outbreaks were often rapidly contained in their area of occurrence.

Table 1: Ebola virus outbreaks

| Viral species | Year | Outbreak location | # of human cases (% fatality) |
|---|---|---|---|
| Zaire ebolavirus | 1976 | Yambuku, Zaire (DRC) | 318 (88%) |
| | 1977 | Tandala, Zaire (DRC) | 1 (100%) |
| | 1994 | Ogooue-Ivindo province, Gabon | 51 (60%) |
| | 1995 | Kikwit, Democratic Republic of Congo | 315 (79%) |
| | 1996 | Mayibout, Gabon | 37 (57%) |
| | 1996 | Booue, Gabon and Johannesburg, South Africa | 61 (74%) |
| | 2001-02 | Ogooue-Ivindo province, Republic of Congo | 124 (79%) |
| | 2002-03 | Cuvette region, RC and Ogooue-Ivindo province, Gabon | 143 (90%) |
| | 2003 | Mboma and Mbandza, Republic of Congo | 35 (83%) |
| | 2005 | Etoumbi and Mbomo, Republic of Congo | 12 (75%) |
| | 2007 | Kasai Occidental province, Democratic Republic of Congo | 25 (not determined) |

| | | | |
|---|---|---|---|
| | 2008/ 2009 | Democratic Republic of the Congo | 32 (47%) |
| Sudan ebolavirus | 1976 | Nzara, Maridi, Tembura, Juba, Sudan | 284 (53%) |
| | 1979 | Nzara, Yambio, Sudan | 34 (65%) |
| | 2000-01 | Gulu, Masindi, Uganda | 425 (53%) |
| | 2004 | Yambio, Sudan | 17 (41%) |
| | 2011 | Uganda (Luwero District) | 1 (100%) |
| Taï ebolavirus | 1994 | Tai forest, Ivory Coast | 1 (0%) |
| | 1995 | Liberia, Liberia | 1995 |
| Reston ebolavirus | 1989 | Reston, USA | 4 (0%) |
| | 1992 | Siena, Italy | 0 |
| | 1996 | Alice, Texas | 0 |
| Bundibugyo | 2008 | Phillipines | 0 |
| ebolavirus | 2007/2008 | Uganda | 131 (37%) |

## 3.2. Pathogenesis of Ebola Virus, Host immune responses and Ebola Virus Disease severity

Ebola virus enters the human host body through mucosal surfaces as well as breaks and abrasions in the skin, or by parenteral introduction. Human-human transmission during outbreaks occurs by direct contact with infected patients or cadavers. In addition, sexual transmission and ingestion of contaminated food may be possible sources of infections with Ebola virus. Immune cells of the infected host including monocytes, macrophages, and dendritic cells contribute to the dissemination of the virus from initial site of infection to regional lymph nodes, probably through the lymphatic system, and to the liver and spleen through the blood. Then the virus invades a wide range of host cells including endothelial cells, fibroblasts, hepatocytes, adrenal cortical cells, and several types of epithelial cells. All these cells provide support for intense replication to the virus. Ebola virus-induced pro-inflammatory mediators including (but not exhaustive) interleukins 2, 6, 8, and 10; interferon-inducible protein 10; monocyte chemo-attractant protein 1, (RANTES); TNF$\alpha$; and reactive oxygen and nitrogen may be intense and imbalanced. Dysregulated pro-inflammatory responses seem to be associated with fatal cases of EVD while timely and adequately regulated inflammatory responses seem to confer recovery. In animal model, dysregulation by inhibition of the type I interferon response may be a key

player in the pathogenesis of Ebola virus and Marburg Virus. Virus particles like VP35 function as a type I interferon antagonist by blocking activation of interferon regulatory factor and possibly by preventing transcription of interferon β. Similarly, expression of VP24 of the Ebola virus interferes with type I interferon signaling pathways and favors the ability of Zaire Ebola virus to induce death in mouse[2]. These imbalanced immune responses are associated with high concentrations of nitric oxide in patients infected with Zaire Ebola virus and Sudan Ebola viruses[2,3,4]. Abnormal production of Nitric oxide may be a negative predictor of Ebola virus induced-disorders including apoptosis of bystander lymphocytes, tissue damage, and loss of vascular integrity and in turn contribute to EVD severe manifestation like hemorrhage and shock.

## 3.3. Vaccine against Ebola Virus

Although no causal treatment and vaccine are so far available to address EVD, researchers have made significant progress over the last years. Currently, the following vaccine platforms in pre-clinical development are demonstrated to be of interest against EBOV and MARV: virus-like-particles (VLPs), Venezuelan equine encephalitis virus replicons (VEEV RP), replication incompetent adenovirus serotype 5 vectors, replication competent recombinant human parainfluenza virus 3 (rHPIV3) and recombinant vesicular stomatitis virus (rVSV)[5,6]. The rVSV platform is one of the most promising vaccine candidate approaches against filoviruses. The

VSV is a nonsegmented, negative-stranded RNA virus in the family Rhabdoviridae, which is an animal pathogen and does not cause serious disease in humans. Two serotypes of the VSV circulate on the American continent, serotypes New Jersey and Indiana[5,7]. Livestock are the preferred host via transmission by mosquitos, sandflies or blackflies. Manifestations are vesicular lesions on the mouth and teats. Humans are rarely infected and even in the event of an infection, the disease course is generally asymptomatic or mild[7]. Several VSV characteristics makes it a good vaccine vector: replication in mammalian cell lines, growth to very high titers, and a strong induction of innate and adaptive (humoral as well as cellular) immune responses, very low levels of pre-existing immunity to VSV in the general population[5]. As an additional benefit, it induces neutralizing immune response primarily directed against the VSV glycoprotein (VSV-G). This characteristic is used in designing the Ebola vaccine. TheVSVΔG-ZEBOV vaccine consists of a recombinant Vesicular Stomatitis Virus (VSV), in which the Ebolavirus Zaire envelope glycoprotein has been substituted to the G coat glycoprotein. Thus the final recombinant vaccine conserves all other VSV functions, but no other Ebolavirus functions except those associated with the envelope glycoprotein.

The recombinant virus can be attenuated and be manufactured at high titers. The use of attenuated rVSV as platform for vaccine against filovirus has been demonstrated to be safe in mouse and NHP. Animal vaccinated with, rVSV-ZEBOV-GP, or rVSV-MARV-GP did not experience any major toxicity including neurotoxicity which is commonly reported in groups of animals infected with the wild type of VSV[8].In pre-exposure pre-clinical studies in NHP, the vaccine showed 100% efficacy . Post-exposure prophylaxis allows survival of 50% of non-human primates. The VSVΔG-ZEBOV vaccine has been given to laboratory worker in conditions of post exposure and showed to be safe.

### 3.4. Rationale of current project

Ebola Virus Diseases (EVD) are historically sporadic outbreaks, which occur in remote areas of tropical forest mainly in Central Africa. Although EVD are associated to a high case fatality rate, Ebola virus transmission was so far low and outbreaks were often rapidly contained in their area of occurrence. Vaccination to prevent infection with EV-outbreaks in human may have been considered impractical due to the sporadic incidence of the EVD despite evidence that recombinant vaccines can be effective in non-human primate models. However, the outbreak, which is occurring in West Africa, has changed the paradigm and a need for vaccine for pre-exposure of health care workers and post exposure of infected patients is now urgent. In contrast to the historical outbreak, this one is associated with an intense transmission in Guinea, Liberia and Sierra Leone. This Western African outbreak EVD is now recognized as a major and global health security concern as it spreads out unprecedentedly to capital cities and across countries; Nigeria and Senegal have so far reported imported cases. The burden on the already weak health systems in the 3 most affected countries is devastating and makes the outbreak out of control. Therefore, WHO is coordinating a global Response Road Map with the goal to stop EVD transmission globally within 6 to 9 months. Among the major mean to reach this objective, prompt clinical evaluation of safety and immunogenicity of existing candidate vaccine is a top priority. The present study aims to provide clinical data on the VSVΔG-ZEBOV candidate vaccine by end of 2014.

# 4. Trial Design

### 4.1. Overview

LA-BPSC10001-01 is a Phase I, open-label, randomized, dose escalation trial. LA-BPSC10001-01 is designed to establish safety, tolerability and immunogenicity of three dose levels of VSVΔG-ZEBOV, an Ebola Virus Vaccine Candidate for the first time in sub-Saharan African populations. In LA-BPSC10001-01 trial, 201 volunteers will be randomized. The study will be conducted in Lambaréné, Gabon. There will be seven cohorts. Cohort 1 will be of participants receiving the dose $3x10^5$ pfu, cohort 2 will receive $3x10^6$pfu of VSVΔG-ZEBOV and cohort 3 $2x10^7$pfu. Cohort 4 will be receiving the lowest dose of $3x10^3$ pfu, followed by cohort 5 which will receive $3x10^4$ pfu. Cohorts 6 and 7 will receive $2x10^7$ pfu. All doses will be administered intramuscularly. Vaccination of subjects in each cohort will proceed in a staggered fashion. Eligible adult volunteers will be randomized into the first 5 study cohorts. However, at the time of the submission of the study amendment 3, 60 eligible participants were already randomized and vaccinated in the cohort 1, 2 and 3 (20 into cohort 1, 39 into cohort 2 and one into cohort 3). Therefore, new randomization list will be generated according to the study requirements of having additional 61 vaccinees in the cohort 3, 20 in the cohort 4 and 20 in cohort 5.

On Day 1, the 1st volunteer of cohort 1 will be vaccinated, followed by the 2nd and 3rd volunteer on Day 3. If no safety concerns arise a group of 5 participants will be vaccinated on day 5. If the holding criteria have not been met and there are no safety concerns, the

next 5-10 participants will be vaccinated. Upon completion of visit 5 by the first 5-10 vaccinees of cohort 1 the DSMB′ will do a review of safety data. Cohorts 2 and 3 will be run in parallel upon the go decision of DSMB after safety data review. Participants will be enrolled in Cohorts 3,4,5,6 and 7 and vaccinated in an identical manner to the Cohort 1 and 2. Children and adolescents will be enrolled and vaccinated into cohort 6 and 7 respectively only after the first 10 participants vaccinated in cohort 3 have reach D 28 follow-up. Following the first 10 participants, volunteers will be vaccinated in groups of 5-10 per day irrespective of the cohort. Vaccinees will be observed 30 min to one hour after vaccination and occurrence of any reaction will be carefully monitored.

For each volunteer, there will be a 180-day period of active follow-up after vaccination. An additional 180 days of passive follow-up may take place upon a study amendment. Overall this study will be completed in a period of maximum 18 months. The primary endpoint will be reached for all study participants within 2 to 3 months.

.

## 4.2. Objectives

**Primary Objectives:**

- To evaluate the safety and tolerability of five different doses of VSVΔG-ZEBOV vaccine in three different age groups.

**Secondary Objectives:**

- To evaluate the ZEBOV-specific antibody responses induced by VSVΔG-ZEBOV vaccine
- To evaluate VSV vaccine viremia and excretion after administration of VSVG-ZEBOV vaccine
- To relate status of pre-vaccination asymptomatic exposure to filoviruses of volunteers to ZEBOV-specific antibody responses

**Exploratory objectives:**

- To evaluate immune response in the context of HLA allele expression
- To assay Ebola protein reactivity by Western Blot of any filovirus seropositives before vaccination
- To evaluate B-cell repertoire after vaccination
- To evaluate T-cell repertoire after vaccination
- To perform T-cell phenotyping after vaccination
- Obtain and preserve specimens to support assays to dissect the mechanism of the post-exposure protection observed in the NHP model
- To evaluate memory B- and T-cell responses up to 6 months after vaccination
- To isolate human antibodies in response to the vaccine

## 4.3. Endpoints

**Primary Endpoints**

The nature, frequency, and severity of adverse events (AEs) and/or serious adverse events (SAEs) associated with administration of BPSC1001 at 5 different doses measured as follow:

- Frequency and severity of local injection site reactogenicity signs and symptoms: pain, erythema, and induration until day 14 post vaccination (solicited local symptoms)
- Frequency and severity of systemic reactogenicity signs and symptoms until day 14 post vaccination (solicited general symptoms)
- Frequency of adverse events (AEs), severity and assessed relationship to study products until day 28 post vaccination (unsolicited AE)
- Detailed description of all serious adverse events (SAEs) until day 28 post vaccination
- Distribution of values of safety laboratory measures at baseline and at follow-up visits post-vaccination until day 28 post vaccination (reference ranges will be age appropriate)
- Number of participants with early discontinuation of vaccinations and reason for discontinuation until day 28 post vaccination

**Secondary endpoints:**

- Detailed description of all serious adverse events (SAEs) until study last visit (month 6)
- Concentration of ZEBOV envelope glycoprotein-specific binding antibody by ELISA
- Concentration of ZEBOV-specific neutralizing antibody at time points as considered in Table 3
- Concentration of rVSV in blood, urine, or saliva as detected by RT-PCR at time points as considered in Table 3

**Exploratory endpoints:**

Exploratory objective will be performed in sub samples of study participants. An experimental plan with detailed information will be specified in the course of the trial. However, blood will be collected and cells cryopreserved in all participants.

- To evaluate pre-vaccination of volunteers status on the ZEBOV-specific antibody responses

- Measurement of ZEBOV envelope glycoprotein-specific binding antibody by competition assays and Western blot analysis, including pre-vaccination positive samples
- Determination of HLA allele status at screening
- Determination of B-cell repertoire before and after vaccination (screening and day 28, 84, 180)
- To evaluate T-cell repertoire before and after vaccination (screening and day 28, 84, 180)

## 4.4. Investigational Products

IDT-BIOLOGIKA GMBH manufactured the investigational product that will be administered via IM injection.

BPSC1001 vaccine is shipped by air carrier to the clinical site on dry ice to maintain the product at ≤-70°C. Each shipment is accompanied by a Clinical Investigational Material Shipment Receipt Form and a Certificate of Analysis for product release testing. Upon receipt, at the clinical site, the product is unpacked by a designated person(s) and immediately transferred to ≤ -70°C storage.

BPSC1001 Dosage Form, Route of Administration and Use:

The vaccine is supplied individually packaged in sterile 1.0 mL vials. Each vial contains vaccine virus frozen in a mixture of USP water for injection, human serum albumin (25%, USP) and 10 mM Tris, ph 7.2. After thawing, the content of the vials is a clear liquid. The vaccine concentrations in the vials are formulated as: 1 x $10^8$ pfu/ml (stock). For dilution to 3 x $10^6$ and 3 x $10^5$ pfu/ml the vaccine can be prepared by mixing 0.32 ml of stock vaccine with 10 ml of normal saline (USP) or, vaccine at 2 x $10^7$ pfu/ml is prepared by mixing 1 ml of stock vaccine with 4 ml of normal saline (USP).One ml of vaccine is administered to the deltoid intramuscularly using a 3 cc syringe with a #25-gauge needle.

The lower doses of 3 x $10^3$ and 3x $10^4$ will be diluted as per the pharmacy manual to be provided by the company.

Table 2: Study products characteristics

| **Product Name** | **BPSC1001** |
|---|---|
| Dosage form | Liquid |
| Unit dose/Injection volume [i)] | 1x$10^8$ pfu/mL (stock) |
| Route of administration | IM injection deltoid region |
| Physical description | Clear, colorless solution |
| Manufacturer | IDT-BIOLOGIKA GMBH |
| Lot number | 003 05013 |
| Product indication | Intended for human use in adults as a prophylactic measure against EBOV infection |

## 4.5. Participation and study Duration

The expected duration of individual enrolled subject participation will be approximately 6-8 or 12 to 14 months from the screening period. The date of signature of the informed consent form by the first volunteer is taken as the study start. The end of the trial is the date of the last visit of the last volunteer.

## 4.6. Dose-adjustment Criteria

**Safety Criteria for Dose Adjustment or Stopping Doses**

There will be no dose modifications.

**Individual Holding Rules**

As volunteers will receive a single vaccine dose, there will be no individual holding rules. Participants not meeting criteria for vaccination will be replaced by alternates.

**Holding Rules**

Participants will be followed a minimum of 60 minutes after vaccination. As such, immediate hypersensitivity reaction (in the 60 minutes following vaccination) will be assessed. All vaccinees will be monitored the day after injection in case of cytokine storm (in the 24 hours following vaccination). If any of the subjects has a vaccine related SAE, vaccinations will be held for the remainder of the study participants until a decision on vaccination resumption is reached. Vaccinations will resume upon agreement of the DSMB, the PI, the sponsor and VEBCON partner.

The following holding rules apply to each cohort of subjects. Solicited and unsolicited AEs and safety laboratory data will be recorded and tracked by the PI after immunization. The research monitor will review the AEs (local, clinical systemic, and laboratory systemic).If any holding rules are met, no further vaccination will be administered in any cohort until the investigators have conferred with the safety monitor and a full written report has been submitted to the Sponsor, the CERMEL SRC, the CERMEL IRB and the Gabonese NEC.

- One or more participants experience any SAE related to the study product (possible, probable or related), or

  A suspected unexpected serious adverse drug reaction (SUSAR) occurs that is life-threatening or results in death.
- If any other observation occurs that in the opinion of the PI results in a recommendation to halt enrollment

After an holding rule is activated, a thorough review by the PI, safety monitor, and DSMB will occur. Vaccination of the subjects may resume only if the DSMB, the PI, and the sponsor agree it is safe to resume vaccinations and the following considerations are discussed.

- Relationship of the AE or SAE to the vaccine

- Relationship of the AE or SAE to the vaccine dose (eg, only associated with high dose group)
- If appropriate, additional screening or laboratory testing is provided to other subjects to identify subjects who may develop similar symptoms
- If any study related SAE is not listed on the current informed consent form (ICF), the PI will revise the ICF and volunteers will be asked to provide consent on the new ICF

The VEBCON partners will be notified if a holding rule is activated or released. If the clinical trial is halted and is unable to resume, a formal letter will be sent to the country specific regulatory authorities by the sponsor explaining the reasons for cessation of the study.

All vaccinated subjects will be followed for safety until resolution or stabilization (if determined to be chronic sequelae) of their AEs.

**Study Termination Criteria**

The PI, sponsor's representative, the DSMB, and the appropriate regulatory authorities (national or international) may stop or suspend the use of this product at any time.

Significant numbers of arthritis cases have occurred among participant receiving the same vaccine, same batch number at high concentration from 1. $10^7$ in Switzerland..

# 5. Selection and withdrawal of trial volunteers

## 5.1. Volunteer recruitment

Both health care workers and other community members, i.e. other adults living in the Lambarene area will be approached. Information on study recruitment is done by:

- Word-of-mouth, whereby health professionals working in the community and volunteers who have already been approached, mention the study to patients/family members/friends who go on to approach the study team. This is not solicited information sharing; merely an assumption that this will happen.
- Active approach by study team members, who will provide the Patient Information Sheet and discuss it with potential volunteers.
- Interviews and presentations (*e.g.* presentations at lectures or seminars).
- Advertising if requested

The investigators should be satisfied that there is no relationship or dependency that could coerce potentially vulnerable CERMEL or other health care workers to participate. In addition to the volunteers to be enrolled in the study, back-up volunteers who have been screened and are eligible to participate in the study will be identified. These volunteers will be available for enrolment at short notice should a planned volunteer withdraw consent or become ineligible immediately prior to enrolment. Backup volunteers will be asked to be available at short notice and may be asked to attend clinic on enrolment days.

## 5.2. Informed Consent

All volunteers need to sign and date the informed consent form before any study specific procedures are performed. The information sheet will be made available to the volunteer at least 24 hours prior to the screening visit. At the screening visit, the volunteer will be fully informed of all aspects of the trial, the potential risks and their obligations. The following general principles will be emphasised:

- Participation in the study is entirely voluntary
- Refusal to participate involves no penalty or loss of medical benefits
- The volunteer may withdraw from the study at any time, as described in section 5.5.
- The volunteer is free to ask questions at any time to allow him or her to understand the purpose of the study and the procedures involved
- There is no direct benefit from participating but travel to and from research site and time spent for participation will be compensated.

The aims of the study and all tests to be carried out will be explained. The volunteer will be given the opportunity to ask about details of the trial, and will then have time to consider whether or not to participate. The volunteer will be encouraged to discuss with their relatives. CERMEL has a research group working on the social components of clinical research. Before, during and after the trial, the study participants will be regularly reminded the purposes and procedures of the trial and clinical research in general. This approach showed to be more effective to ensure the understanding of the trial purposes by study participants.

Informed consent for children:

- Parents or guardians will be required to provide informed consent for minors (under the ages of 18), adhering to the same principles as above. This includes minors who are married.
- Assent will be sought from minors aged 11 to 17 years by engaging them in conversation about the study and ensuring they are not unwilling to participate. They will be asked to sign an assent document.
- Both assent and parental consent must be obtained for a minor aged 11 to 17 years to be enrolled in the study.
- As with recruitment for the adults, field workers who contact households are members of the community. They will seek to engage families, and will ensure minors and their parents/guardians will be given adequate time to discuss the study before being contacted again about consent.
- Contraceptive recommendations for minors will be primarily condoms; however oral contraception is also available. Parents will be made aware of contraceptive prescriptions for minors.
- Information on pregnancy and contraception will be provided to female minors who have reached menses, and to their parents in the assent and consent for parents/guardians. This will detail pregnancy testing, results and contraception.

## 5.3. Inclusion Criteria

The volunteer must satisfy all the following criteria to be eligible for the study:

- Healthy adult male or non-pregnant, non-lactating female, ages 18 to 50 (inclusive) at the time of screening (cohorts 1-5)
- Healthy male or non-pregnant female, ages 6-12 (inclusive) for cohort 6 and ages 13-17 –(inclusive) for cohort 7, at the time of screening.
- Have provided/had their guardians provide written informed consent/assent as appropriate, prior to screening procedures Free of clinically significant health problems, as determined by pertinent medical history and clinical examination prior to entry into the study
- Available, able, and willing to participate for all study visits and procedures
- Negative pregnancy-test for female volunteers
- Females, of childbearing potential, who are willing to use one month after vaccination effective methods of contraception.
    - A woman is considered of childbearing potential unless post-menopausal (≥ one year without menses) or surgically sterilized (tubal ligation, bilateral oophorectomy, or hysterectomy)
    - Effective contraception is defined as a contraceptive method with failure rate of less than 1% per year when used consistently and correctly and when applicable, in accordance with the product label for example:
        - Oral contraceptives, either combined or progestogen alone,
        - injectable progestogen,
        - implants of etenogestrel or levonorgestrel,
        - oestrogenic vaginal ring
        - percutaneous contraceptive patches,
        - intrauterine device or intrauterine system,
        - male partner sterilisation at least 6months prior to the female subject's entry into the study, and the relationship is monogamous,
        - male condom male condom
- Be willing to minimize blood and body fluid exposure of others for 5 days after vaccination
    - Be advised to use an effective barrier prophylaxis, such as latex condoms, during penetrative sexual intercourse
    - Avoiding the sharing of needles, razors, or toothbrushes
    - Avoiding open-mouth kissing

## 5.4. Exclusion Criteria

The volunteer will not enter the study if any of the following apply:

- History of severe local or systemic reactions to any vaccination or a history of severe allergic reactions
- Known allergy to the components of the BPSC1001 vaccine product

- Ongoing participation in another clinical trial
- Receipt of licensed vaccines within 14 days of planned study immunization (30 days for live vaccines)
- Acute or chronic, clinically significant psychiatric, hematologic, pulmonary, cardiovascular, or hepatic or renal functional abnormality as determined by the investigator based on medical history, physical exam, ECG, and/or laboratory screening test (including sickle cell anemia)
- Viral Serologies (HBsAg, anti-HCV Ab, anti-HIV Ab)
- Any confirmed or suspected immunosuppressive or immunodeficient condition, cytotoxic therapy in the previous 5 years, and/or diabetes
- Any chronic or active neurologic disorder, including migraines, seizures, and epilepsy, excluding a single febrile seizure as a child
- Have a known history of Guillain-Barré Syndrome
- Have an active malignancy or history of metastatic or hematologic malignancy
- Suspected or known alcohol and/or illicit drug abuse within the past 5 years
- Moderate or severe illness and/or fever >38°C within 1 week prior to vaccination
- Pregnant or lactating female, or female who intends to become pregnant during the study period
- Administration of immunoglobulins and/or any blood products within the 120 days preceding study entry or planned administration during the study period
- History of blood donation within 60 days of enrollment or plans to donate within the study period
- Administration of chronic (defined as more than 14 days) immunosuppressants or other immune-modifying drugs within 6 months of study entry
    - For corticosteroids, this will mean prednisone, or equivalent, greater than or equal to 0.5 mg/kg/day
    - Intranasal and topical steroids are allowed
- Have an immunocompromised household member
- Any other significant finding that in the opinion of the investigator would increase the risk of the individual having an adverse outcome from participating in this study

## 5.5. Withdrawal of Volunteers

In accordance with the principles of the current revision of the Declaration of Helsinki (updated 2008) and any other applicable regulations, a volunteer has the right to withdraw from the study at any time and for any reason, and is not obliged to give his or her reasons for doing so. A parent or guardian has the right to withdraw a minor from the study at any time and for any reason, and is not obliged to give his or her reasons for doing so. Minors aged 11 and above have the right to withdraw their assent at any time and for any reason, and are not obliged to give their reasons for doing so. The investigator may withdraw the volunteer at any time in the interests of the volunteer's health and well-being. In addition the volunteer may withdraw/be withdrawn for any of the following reasons:

- Administrative decision by the investigator. This may include but is not limited to, ill health of the participant, endangerment of study staff or other participants by the behaviour of the volunteer, a possible or probable adverse event.
- Ineligibility (either arising during the study or retrospectively, having been overlooked at screening). If participants have already received a vaccination prior to becoming (or being discovered to be) ineligible, they will only be withdrawn if continued participation jeopardizes their health.
- Significant protocol deviation, which as described in ICH GCP guidelines, include those which have an impact on the safety of a participant.
- Volunteer non-compliance with study requirements
- An AE, which requires discontinuation of the study involvement or results in inability to continue to comply with study procedures

The reason for withdrawal will be recorded in the CRF. If withdrawal is due to an AE, appropriate follow-up visits or medical care will be arranged, with the agreement of the volunteer, until the AE has resolved, stabilised or a non-trial related causality has been assigned. Any volunteer who is withdrawn from the study may be replaced, if that is possible within the specified time frame. The Local Safety Monitor (LSM) may recommend withdrawal of volunteers. If a volunteer withdraws from the study, blood samples collected before their withdrawal from the trial will be used/stored unless the volunteer specifically requests otherwise.

.

## 5.6. Pregnancy

Should a volunteer become pregnant during the trial, she will be followed up as other volunteers and in addition will be followed until pregnancy outcome. This follow up is not only related to the health of the pregnant woman, but also includes evaluation of the new born and other pregnancy outcomes. Non-essential venepunctures will not be performed on such volunteers.

## 5.7. Potential risks for volunteers

**Phlebotomy:**

The maximum volume of blood drawn over the study period (700 ml over 6 to 12 months, or 70 ml for children and adolescents, as described in table 3) should not compromise these otherwise healthy volunteers. There may be minor bruising, local tenderness or pre-syncopal symptoms associated with venepuncture, which will not be documented as AEs if they occur.

**Administration of study vaccines:**

Serious allergic reactions including anaphylaxis may occur and for this reason volunteers will be inoculated in a clinical area where Advanced Life Support trained physicians, equipment and drugs are immediately available for the management of any serious adverse reactions. Participants will be observed for one hour after vaccination.

**VSV-EBOV infection*:***

The vaccine has been tested in animals, including non-human primates, and it is safe for them. The risk related to replication of attenuated VSV after being injected with VSVΔG-ZEBOV vaccine is unlikely. However, volunteers will be carefully monitored. In a German laboratory worker who received a laboratory preparation of the vaccine, replication occurs temporarily at day 2 but at low titre in plasma, and no serious safety problem was noted. In monkeys the virus replicates for up to 2-3 days at a low level without any safety problems. A related VSV vaccine developed by a different manufacturer has been administered to 80 volunteers in USA without serious safety concerns.

The current formulation of the vaccine has been constructed by deleting the entire G protein, which is required for VSV infection, so reversion to wild type VSV is not possible. The deletion of entire G protein means that the vaccine VSV backbone is highly attenuated compared to wild type VSV.

There is a very low risk that, vaccinees may experience virus replication at a level that VSV becomes transmissible to others. Although unlikely, participants will be advised for prevention measures and VSV virus replication shall be closely monitored. If requested, anti-HIV protease inhibitors may be considered in case of out-of-control VSV replication.

.

### 5.8. Potential benefits for volunteers

This trial is being conducted in a context of an EVD unprecedented outbreak, which is considered as a major and global health security concern. The vaccine recipients may benefit from protection against future EVD as Gabon has experienced also previous outbreaks. However, this trial is the first administration of VSVΔG-ZEBOV vaccine to humans. Similar trials will be performed with this vaccine coincidently at sites in Germany and Switzerland. So the risks-benefits ratio is well known although no major or severe side effect occurred so far across the trials which are ongoing in Gabon, Kenya, Germany, Switzerland and US. There is also a rise of antibodies level in the vaccinees. Participants will be advised to not consider themselves protected against EVD at this stage of vaccine development.

## 6. Study procedures

### 6.1. Time points

Procedures will be performed at the time points indicated in the schedule of procedures (Table 3). Additional procedures or laboratory tests may be performed, at the discretion of the investigators if clinically necessary.

### 6.2. Screening Visit

Screening Visit 1; Day -30 to Day -1

The Participant Information document will be provided during the recruitment phase, and before screening visits are scheduled.

Potential subjects will undergo screening no more than 30 days prior to their planned initial injection (Day 0, Visit 2). Any potential subject falling outside the 30-day window will require rescreening.

The following procedures will be performed during the screening visit:

- Briefing and review of informed consent with subject
- Written informed consent for participation in the clinical study.
- Provision of copy of signed informed consent form to subject
- Check of inclusion and exclusion criteria
- Provision of demographic information and medical history by subject (including concomitant medications)
- Physical examination
- Blood Collection: approximately 120 mL whole venous blood (10mL for children and 15mL for adolescents) for the determination of:

WBC, ALT, Creatinin, Viral Serologies (HBsAg, anti-HCV Ab, anti-HIV Ab), VSV virus isolation and replication, further analysis for humoral and cellular immune responses

Saliva and urine for VSV virus isolation

Urine pregnancy test (females only)

Potential subjects will be notified of their eligibility as soon as all lab results are available and the investigator can make an assessment. If determined to be eligible, subjects will be scheduled for their initial vaccination (Day 0, Visit 2).

Regardless of eligibility, individuals will be informed of any significantly abnormal test results, in person. Appropriate counseling will be given regarding necessary medical follow-up.

Information on individuals with evidence of reportable infectious diseases (eg. HIV and hepatitis), will be addressed to the required public health institution as applicable. Potential subjects will be notified of this possibility during their informed consent process.

Priority for blood collected from minors will be given to vaccine specific antibody tests and rVSV detection. Remaining blood will be used for cellular immune responses (if feasible).

### 6.3. Study Enrollment/ vaccination (Visit 2, Day 0)

Vaccination with BPSC1001 will occur at Visit 2. Potential participants will be listed upon information sessions and their willingness to participate. They will be then screened by block of 5-10 volunteers after informed consent signature. Eligible participants will be given a study identification number which for adults will be used for random selection to one of 5 cohorts. The randomization will be done using a software. The randomization will be detailed in a study procedure.

Subjects will have only one vaccination visit during the course of the study. Each subject will receive an injection of BPSC1001 into the outer aspect of the subject's upper arm (deltoid area). The following procedures will be performed during each injection visit:

- Review of subject's medical history (including concomitant medications) since previous visit

- Check of inclusion / exclusion criteria
- Directed physical examination (proposed injection site) and vital signs check
- Urine sample collection for determination of:
  Urine pregnancy test (females only)*
- Injection with study product
- Post-injection observation period of at least 60 min.
- Study visits schedule , thermometer ( for axillary temperature), and ruler distribution and review of use
- Provision of emergency contact card
- Repeat examination of injection site and vital signs check
- Review of any immediate AE

*All female subjects of childbearing potential will be required to submit a urine sample for onsite pregnancy testing on the day of injection, and the test results must be negative prior to administration of the vaccine.

## 6.4. Follow-Up Visits: visit 3 to visit 10

There will be 8 follow-up visits (Visits 3 through 10). Table 3 outlines visits, study days, and visit windows. The following procedures will be performed during each follow-up visit:

- Review of subject's medical history (including concomitant medications and AE) since previous visit
- Directed physical examination and vital signs check
- Review solicited symptoms (4 visits; Visits 3, 4, 5, 6, )
- Collection of approximately 6 to 92 mL whole venous blood for the determination of:
  - WBC
  - Serum chemistry profiles (Creatinin, AST, ALT) will be performed only at visits 5,7,9,10.
  - Blood for immunological analyses
  - Blood for virus isolation
- Urine and saliva for virus isolation

Visit 10 (month 6) will be the final scheduled visit for all subjects. Subjects completing this visit will be considered to have completed the study. Subjects with ongoing AE at this visit will be followed until they have returned to normal, stabilized, or the event is otherwise explained or the subject is lost to follow-up.

## 6.5. Safety assessment

Safety monitoring will be conducted throughout the study; therefore, safety concerns will be identified by continuous review of the data by the PI, clinic staff, clinical monitor, safety monitor, DSMB, and sponsor.

**Specification of Safety Endpoints**

Safety will be assessed by evaluating reactogenicity (local reactions and systemic reactions such as fever, headache, and fatigue) during specified periods of the study. The following endpoints will be evaluated:

The nature, frequency, and severity of solicited AEs occurring from the time of injection through 14 days following the procedure (memory aid to assist)

The nature, frequency, and severity of unsolicited AEs from the time of injection through 28 days following the each injection

The nature, frequency, and severity of SAEs from the time of injection through the final study visit for each subject

**Solicited Adverse Events**

A solicited AE is a predetermined event, which may reflect safety concerns related to the investigational product. The solicited AEs for this study include:

- Swelling, or pain at site of injection
- Fever
- Chills
- Myalgia (described to the subject as generalized muscle aches)
- Arthralgia (described to the subject as generalized joint aches)
- Swelling of joint(s) or pain on motion of joint(s)
- Rash or blisters of the skin
- Blisters or ulcers in mouth, tongue or gums
- Fatigue
- Headache
- Gastrointestinal symptoms (nausea, vomiting, abdominal pain, and/or diarrhea)

Participants with objective signs of arthritis (swelling, tenderness, warmth etc) will be evaluated with ultrasound.

Vesicular lesions and joint effusions (if tapped) will be sampled for PCR and virus isolation.

### Serious Adverse Event or Serious Suspected Adverse Reaction

An AE or suspected adverse reaction is considered "serious" if, in the view of either the investigator or sponsor, it results in any of the following outcomes:

- Death
- Life-threatening adverse event defined as any adverse drug experience that, in the opinion of the investigator, places the subject at immediate risk of death from the reaction as it occurred. It does not include a reaction that, had it occurred in a more severe form, might have caused death.
- Inpatient hospitalization or prolongation of existing hospitalization
- Persistent or significant disability/incapacity defined as a "substantial disruption of the ability to conduct normal life functions"
- Congenital anomaly/birth defect (in the offspring of a subject)

An AE or suspected adverse reaction is considered "life-threatening" if, in the view of either the investigator or sponsor, its occurrence places the patient or subject at immediate risk of death. It does not include an adverse event or suspected adverse reaction that, had it occurred in a more severe form, might have caused death.

Important medical events that may not result in death, be life-threatening, or require hospitalization may be considered serious when, based upon appropriate medical judgment, they may jeopardize the patient or subject and may require medical or surgical intervention to prevent one of the outcomes listed in this definition. Examples of such medical events include allergic bronchospasm requiring intensive treatment in an emergency room or at home, blood dyscrasias or convulsions that do not result in inpatient hospitalization, or the development of drug dependency or drug abuse.

### Unexpected Adverse Event or Unexpected Suspected Adverse Reaction

An AE or suspected adverse reaction is considered "unexpected" if it is not listed in the investigator brochure or is not listed at the specificity or severity that has been observed; or, if an investigator brochure is not required or available, is not consistent with the risk information described in the general investigational plan or elsewhere in the current application, as amended. For example, under this definition, hepatic necrosis would be unexpected (by virtue of greater severity) if the investigator's brochure referred only to elevated hepatic enzymes or hepatitis. Similarly, cerebral thromboembolism and cerebral vasculitis would be unexpected (by virtue of greater specificity) if the investigator's brochure listed only cerebral vascular accidents. "Unexpected," as used in this definition, also refers to adverse events or suspected adverse reactions that are mentioned in the investigator brochure as occurring with a class of drugs or as anticipated from the pharmacological properties of the drug, but are not specifically mentioned as occurring with the particular drug under investigation.

**Unanticipated Problems Involving Risks to Subjects Or Others**

Unanticipated problems involving risks to subjects or others must be reported to the IRB within 48 hours. These events encompass a broader category of events than SAEs and may include issues such as problems with loss of control of subject data or the investigational product; adverse psychological reactions; or breach of confidentiality. Risks to others (eg, program personnel) must also be reported.

Unanticipated problems involving risks to subjects or others are any incident, experience, or outcome that meets all of the following criteria:

- Unexpected (in terms of nature, severity, or frequency) given (a) the procedures that are described in the protocol, investigators brochure or informed consent document; and (b) the characteristics of the subject population;
- Related or possibly related to a subject's participation in the study; and
- Suggests that the study places subjects or others at a greater risk of harm than was previously known or recognized.

The DSMB will evaluate the PI's and research monitor's reports to determine whether a given incident, experience or outcome constitutes an unanticipated problem involving risk to subjects or others and, in coordination with the sponsor, ensure upward reporting of the unanticipated problems involving risk to subjects or others to the appropriate regulatory offices.

Relationship to Investigational Product

The investigator must assign a relationship of each AE to the receipt of the investigational product. The investigator will use clinical judgment in conjunction with the assessment of a plausible biologic mechanism, a temporal relationship between the onset of the event in relation to receipt of the investigational product, and identification of possible alternate etiologies including underlying disease, concurrent illness or concomitant medications. The following guidelines should be used by investigators to assess the relationship of an AE to study product administration. Physicians will make this determination

- Not related: No relationship to investigational product. Applies to those events for which evidence exists that there is an alternate etiology.
- Unlikely: Likely unrelated to the investigational product. Likely to be related to factors other than investigational product, but cannot be ruled out with certainty.
- Possible: An association between the event and the administration of investigational product cannot be ruled out. There is a reasonable temporal association, but there may also be an alternative etiology such as the subject's clinical status or underlying factors including other therapy.

- Probable: There is a high degree of certainty that a relationship to the investigational product exists. There is a reasonable temporal association, and the event cannot be explained by known characteristics of the subject's clinical state or factors including other therapy.
- Definite: An association exists between the receipt of investigational product and the event. An association to other factors has been ruled out.

Severity Assessment

All AEs will be assessed for severity by the investigator. Inherent in this assessment is the medical and clinical consideration of all information surrounding the event including any medical intervention required. Each event will be assigned one of the following categories: mild, moderate, severe, or life-threatening. Refer to the grading scale in annex 2 for further guidance in the assignment of severity. The criteria below may be used for any symptom not included in the grading scale. Any grade 4 (life-threatening) AE must be reported as an SAE.

The CRF for AEs will reflect only the highest severity for continuous days an event occurred.

- Mild Grade 1 Does not interfere with routine activities
  Minimal level of discomfort
- Moderate Grade 2 Interferes with routine activities
  Moderate level of discomfort
- Severe Grade 3 Unable to perform routine activities
  Significant level of discomfort
- Potentially life-threatening Grade 4 Hospitalization or ER visit for potentially
  life-threatening event

FDA guidelines for toxicity will be followed; however, if a subject is evaluated in an emergency room for nonlife threatening illness or symptoms (ie, visits emergency department on weekend for mild problems because the physician's office is closed), the information from that visit will be reviewed and severity of the adverse event will be assessed according to the subject's clinical signs and symptoms.

As defined by the ICH guideline for GCP, the term "severe" is often used to describe intensity (severity) of a specific event (as in mild, moderate, or severe myocardial infarction); the event itself however, may be of relatively minor medical significance (such as severe headache). This is not the same as "serious", which is based on subject/event outcome or action criteria usually associated with events that pose a threat to a subject's life or functioning. Seriousness (not severity) serves as a guide for defining regulatory reporting obligations.

Recording Adverse Events

Methods/Timing for Assessing, Recording, and Analyzing Safety Endpoints

Solicited AEs will be reviewed within the 14 days of vaccination utilizing the provided study memory aids. When a diagnosis is known, the AE term recorded on the CRF will be the diagnosis rather than a constellation of symptoms. The investigator will assess all AEs for seriousness, relationship to investigational product, severity, and other possible etiologies. When an event has not resolved by study closure, it will be documented on the AE CRF as "ongoing".

The timeframe for the collection of unsolicited AEs begins at the first administration of investigational product through 28 days after the investigational product is administered. Solicited AEs will be collected from the time of administration of investigational product through 14 days following the procedure (assisted with a memory aid). The timeframe for the collection of SAEs begins at the first administration of investigational product through the end of the study.

All adverse events will be reported in concordance with ICH GCP reporting guidelines, with expedited reporting of SAEs being determined by these guidelines as well.

## 6.6. Immunogenicity Assessment

Specification of Immunogenicity

Primary Immunogenicity Endpoint:

The primary immunogenicity endpoint is the ZEBOV-specific antibodies geometric mean titre via conventional ELISA. This information will allow calculation of seroconversion rates.

Secondary Immunogenicity Endpoint:

The secondary immunogenicity endpoint will be production of ZEBOV neutralizing antibodies after vaccination as measured by PsVNA. GMT as measured by PsVNA and PRNT50 titers on select samples will be determined for additional information.

Exploratory Endpoints:

There are multiple exploratory endpoints related to the immunological response to the vaccine. Endpoints include:

- Cross-neutralizing antibodies GMT using other Ebola PsV (eg, Sudan)
- Epitope studies with Ebola glycoprotein as target
- Human subjects will be genotyped based on HLA allele expression.
- Evaluation of the B cell repertoire before and after vaccination via Ig VDJ rearrangement sequencing and Ig heavy chain characterization
- Serum immunoglobulin characterization via mass spectrometry/proteomic analysis
- Characterization of epitope targeting for humoral response via domain programmable arrays
- Analysis of T cell repertoire via T cell receptor characterization
- Using multicolor flow cytometry, characterization of cellular response to vaccine antigen or peptides by intracellular cytokine staining (ICS) and the presence of surface markers that indicate cell lineage (CD4+, CD8+), activation status, and expression of selected activation markers.

- Isolation of Human Antibodies

  The vaccine is eliciting a broad range of neutralizing antibodies. The genetic information of these antibodies can be isolated by the technology of phage display. For this, whole blood needs to be preserved in one Serum tube (2.5ml whole blood) and four PAXgene Blood RNA tubes (each tube à 2.5 ml whole blood). This blood withdrawal is needed 4 weeks after the last boost. From this blood whole RNA will be isolated, cDNA generated and the gene information from the variable fragments of the antibodies cloned into an appropriate phagemid system. From the resulting Fab-Phage Display Library, specific antibody fragments will be isolated, recombinantly produced and characterized for specific binding. The parallel blood withdrawal of serum is needed for preliminary tests of the blood samples for specific antigen reactivity.

| | **Visit 1 Screening Day -30-/ -1** | **Visit 2 Vaccination D0** | **Visit 3 Follow-up D1** | **Visit 4 Follow-up D2** | **Visit 5 Follow-up D7** | **Visit 6 Follow-up D14** | **Visit 7 Follow-up D28+/-7** | **Visit 8 Follow-up D56+/-7** | **Visit 9 Follow-up D84+/-7** | **Visit 10 Follow-up D180+/-14** | |
|---|---|---|---|---|---|---|---|---|---|---|---|
| **Community engagement** | **X** | | | | | | **X** | | | **X** | **X** |
| Informed consent | X | | | | | | | | | | |
| Full History | X | | | | | | | | | | |
| Clinical examination | X | | | | | | | | | | |
| Safety labs | X | | | | X | | X | | X | X | |
| Additional Screening Labs | X | X | | | | | | | | | |
| medical history demo information | X | X | X | X | X | X | X | X | X | X | |
| Inclusion/exclusion criteria | X | X | | | | | | | | | |
| pregnancy test counseling | X | X | | | | | | | | | |
| Randomization | | X | | | | | | | | | |
| Review study visits and provide rulers, and thermometers | | X | | | | | | | | | |
| Provide emergency contact cards | | X | | | | | | | | | |
| Record concomitant medications | X | X | X | X | X | X | X | X | X | X | |
| Physical Exam | X | X | X | X | X | X | X | X | X | X | |

| | | | | | | | | | | | |
|---|---|---|---|---|---|---|---|---|---|---|---|
| Monitor AEs | | X | X | X | X | X | X | | | | |
| Monitor SAEs | | X | X | X | X | X | X | X | X | X | |
| Immunogenicity | | | | | | | | | | | |
| Serological analysis/repository | X | | | | X | X | X | X | X | X | |
| T cell repertoire analysis | X | | | | X | | X | | X | X | |
| B cell repertoire analysis | X | | | | X | | X | | X | X | |
| Cellular repository | X | | | | X | | X | | X | X | |
| Viral isolation | X | | X | X | X | | | | | | |
| Antibody isolation | | | | | | | X | | | | |
| Blood Volume/visit (mL) adults | 140 | | 6 | 6 | 75 | 60 | 108 | 20 | 80 | 92 | |
| Cumulative volume for study adults | 140 | | 146 | 152 | 227 | 287 | 395 | 415 | 495 | 587 | |
| Blood Volume/visit (mL) children and adolescents | 10 | | | 5 | 5 | 10 | 10 | 10 | 10 | 10 | |
| Cumulative Blood Volume/visit (mL) children and adolescents | 10 | | | 15 | 20 | 30 | 40 | 50 | 60 | 70 | |

**Table 3:** Study procedures.

# 7. Statistics

Data analysis will consist of descriptive summaries for treatment groups. For primary endpoints, frequency on safety and reactogenicity events will be reported. For secondary and tertiary endpoints descriptive summaries and plots over the time course for both individual patient results and groups will be presented. Where appropriate highly skewed data will be log-transformed and presented as geometric means with 95% confidence intervals. Inference will be conducted with selected tests. For instance Fisher's exact test is used to compare seroconversion rate and percent of subject reporting AE between experimental groups. Logistic regression is used to evaluate the association between demographic information and seroconversion rate.

# 8. Quality control and quality assurance procedures

## 8.1. Investigator procedures

Approved site-specific SOPs will be used at all clinical and laboratory sites.

## 8.2. Monitoring

Monitoring will be performed according to ICH Good Clinical Practice (GCP) by a local monitor. Following written standard operating procedures, the monitors will verify that the clinical trial is conducted and data are generated, documented and reported in compliance with the protocol, GCP and the applicable regulatory requirements. The investigator sites will provide direct access to all trial related source data/documents and reports for the purpose of monitoring and auditing by the sponsor and inspection by local and regulatory authorities.

## 8.3. Modification to protocol

No amendments to this protocol will be made without consultation with, and agreement of, the sponsor and WHO. Any amendments to the trial that appear necessary during the course of the trial must be discussed by the Investigator, the sponsor and WHO. All VEBCON partners will be informed about protocol amendment. If agreement is reached concerning the need for an amendment, it will be produced in writing by the Principal Investigator and will be made a formal part of the protocol following ethical and regulatory approval.

The Investigator is responsible for ensuring that any major changes to an approved trial, during the period for which ethical approval has already been given, are not initiated without EC's review and approval, except to eliminate apparent immediate hazards to the volunteer.

### 8.4. Protocol deviation

Any deviations from the protocol will be documented in a protocol deviation form and filed in the trial master file. Protocol deviations will be reported to local committees according to their procedures and requirements.

### 8.5. Audit and inspection

The QA manager at the trial site will conduct internal audits to check that the trial is being conducted, data recorded, analysed and accurately reported according to the protocol, trial SOPs and incompliance with ICH GCP. The audits will also include laboratory activities according to an agreed audit schedule. The internal audits will supplement the external monitoring process and will review processes not covered by the external monitor.

The sponsor may carry out an audit to ensure compliance with the protocol, GCP and appropriate regulations.

### 8.6. Trial Progress

The progress of the trial will be overseen by the Principal Investigator and the steering committee of VEBCON partnership. .

## 9. Ethics

### 9.1. Declaration of Helsinki

The Investigator will ensure that this study is conducted according to the principles of the current revision of the Declaration of Helsinki 2008.

### 9.2. ICH Guidelines for Good Clinical Practice

The Investigator will ensure that this study is conducted in full conformity with relevant regulations and with the ICH guidelines for GCP (CPMP/ICH/135/95) July 1996.

### 9.3. Informed Consent

Written, informed consent will be obtained, as described above.

### 9.4. Research Ethics Committee

Ethical review will be done by institutional review boards of the research sites and WHO as well as by National Ethic committee of each Gabon and Kenya Principal Investigators

will submit and, where necessary, obtain approval for all subsequent substantial amendments to the protocol and informed consent document.

### 9.5. Volunteer Confidentiality

All data will be attributed a unique study number in the database to identify volunteer data. Separate confidential files containing identifiable information will be stored in secured locations. Only the sponsor representatives, investigators, and the monitor and VEBCON partners will have access to the records. Ethic committees, external auditors and regulatory authorities will also have access to the data

### 9.6. Volunteer consent withdrawal

Volunteers may withdraw from the study at any time. Refer to section 5.5

## 10. Data handling and record keeping

### 10.1. Data Handling

The Principal Investigator or his designee will be the data manager with responsibility for delegating the receiving, entering, cleaning, querying, analysing and storing all data that accrues from the study. All data will be entered in paper case record forms and transcribed by double entered into an electronic database. This includes safety data, laboratory data (both clinical and immunological) and outcome data. Volunteers can decide at any time if samples and data may be used later or will be permanently destroyed.

### 10.2. Record Keeping

All files and source documents will be kept confidentially in locked safety cabinets. The Principal Investigator, co-investigators and clinical research nurses will have access to records. The investigators will permit authorized representatives of the sponsor, regulatory agencies and the monitors to examine (and when required by applicable law, to copy) clinical records for the purposes of quality assurance reviews, audits and evaluation of the study safety and progress.

### 10.3. Sample storage

Biological material will be stored securely at CERMEL for 15 years unless a volunteer opts out, after which time samples will be discarded. At the end of the trial, samples are anonymized without the possibility to backtrack the identity of the volunteer. Samples may be used for future analyses of immunogenicity, virology and other tests related to EVD and to the vaccine candidate.

### 10.4. Source Data and Case Report Forms (CRFs)

All protocol-required information will be collected in CRFs designed by the investigator. All source documents will be filed in the CRF. Source documents are original documents, data, and records from which the volunteer's CRF data are obtained. For this study these

will include, but are not limited to; volunteer consent form, blood results, laboratory records and correspondence. In the majority of cases, CRF entries will be considered source data as the CRF is the site of the original recording (i.e. there is no other written or electronic record of data). In this study this will include, but is not limited to medical history, medication records, vital signs, physical examination records, urine assessments, blood results, adverse event data and details of study interventions. All source data and volunteer CRFs will be stored securely.

### 10.5. Data Protection

The study protocol, documentation, data and all other information generated will be held in strict confidence. No information concerning the study or the data will be released to any unauthorized third party, without prior written approval of VEBCON steering committee. Results from interim analyses will be discussed publicly under the supervision of WHO VEBCON steering committee and WHO will have access to the DSMB. Data from interim analyses and DSMB reports will serve WHO to decide the use of the candidate vaccine beyond Phase 1 as the trial progresses.

It has been agreed that the sites themselves own the data and it is agreed that publication will occur in a timely manner.

### 10.6. Sample Shipments

Samples will be sent internationally for analyses. These will include but are not limited to antibody isolation, ELISA antibody assays and PCR viral isolation. Technical service agreements will be signed between analysing laboratories and CERMEL. Samples will be de-identified, and storage of samples in these laboratories will not be kept for longer than 15 years, as per 10.3.

## 11. Financing and insurance

### 11.1. Financing

The study will be funded primarily by Wellcome Trust, UK, Bundesministerium für Bildung und Forschung (BMBF), Germany and The Bill and Melinda Gates Foundation.

### 11.2. Insurance

The trial will be covered by clinical trial insurance.

### 11.3. Compensation of volunteers

Volunteers are compensated or the time spent in the clinical trial and associated travel costs according to the number and duration of visits in the study center. Each day is compensated according local rules and incomes..

### 11.4. Treatment costs

Treatment costs will be covered by project funds. Costs for hospitalization are covered either by project funds or the insurance (depending on the reason for hospital admission).

## 12. Community awareness and engagement

**Background**

Community awareness (CA) of clinical research purpose and procedures as well as community engagement (CE) are recognized as essential for understanding by the communities and sustainability of clinical research activities, especially in poor resources settings of sub-Saharan Africa.

During the process of ethical clearance and regulatory approval of the LA- BPSC1001-01 study, the CEMEL has been recommended to establish and maintain along and beyond the study period an active community based communication to address potential misconception and misinterpretation in the community about Ebola diseases and Ebola vaccine trials.

Our research center has recently established a social sciences research group with the goal to investigate, to promote and to strengthen CA and CE about clinical research activities. Thus, Ethical and regulatory boards′ recommendations will be incorporated into scientific research questions and addressed in the most appropriate ways.

Together, an LA- BPSC1001-01 vaccine trial offers framework and opportunity to address scientific questions to understand societal factors and relations underlying CA about Ebola disease, vaccine development against Ebola as well as factors supporting CE for early clinical development of vaccines. This research will also support the communication strategies of the main trial according to Ethic and Regulatory recommendations.

**Objectives**

As part of LA- BPSC1001-01study,:

CA awareness about clinical research purpose and procedures and Ebola disease will be investigated prior enrolment, during the study and after the study conduct and upon availability of study results.

Social environment of study participants will be described and their impact on compliance to study procedures and completion of study procedures will be investigated.

Understanding of study specific procedures and methods will be continuously promoted until study completion

Disseminating clinical research purpose and procedure to the immediate social environment of participants of LA- BPSC1001-01 trial as well as to broader community

**Methods**

Nature and dynamic of Awareness about Ebola Disease and Ebola vaccine development of participants and characterization of participants′ social environment will be evaluated through semi-open questionnaires and in-depth interviews.

Participants′ social environment will be described and the nature and the dynamic of their impact on compliance to study procedure will be studied through semi-open questionnaires and in-depth interviews.

Focus Group Discussions will be organized to promote improved awareness and engagement.
CA and CE strength and underlying factors will be investigated before, during and after study conduct.

Study participants with their immediate social relationship and broader community will be targeted to improve CA and CE towards clinical research especially vaccine research.

## 13. References


1. Towner JS, Sealy TK, Khristova ML, et al. Newly discovered Ebola virus associated with hemorrhagic fever outbreak in Uganda. *PLoS Pathog*. 2008;4. doi:10.1371/journal.ppat.1000212.

2. Feldmann H, Geisbert TW. Ebola haemorrhagic fever. *Lancet*. 2011;377:849–862. doi:10.1016/S0140-6736(10)60667-8.

3. Baize S, Leroy EM, Georges AJ, et al. Inflammatory responses in Ebola virus-infected patients. *Clin Exp Immunol*. 2002;128:163–168. doi:10.1046/j.1365-2249.2002.01800.x.

4. Sanchez A, Lukwiya M, Bausch D, et al. Analysis of human peripheral blood samples from fatal and nonfatal cases of Ebola (Sudan) hemorrhagic fever: cellular responses, virus load, and nitric oxide levels. *J Virol*. 2004;78:10370–10377. doi:10.1128/JVI.78.19.10370-10377.2004.

5. Marzi A, Feldmann H, Geisbert TW. Vesicular Stomatitis Virus-Based Vaccines for Prophylaxis and Treatment of Filovirus Infections. *J Bioterror Biodef*. 2011;01. doi:10.4172/2157-2526.S1-004.

6. Falzarano D, Geisbert TW, Feldmann H. Progress in filovirus vaccine development: evaluating the potential for clinical use. *Expert Rev Vaccines*. 2011;10:63–77. doi:10.1586/erv.10.152.

7. Howley, P. M. & Lowy DR. Fields Virology. In: *Wolters Kluwer/Lippincott Williams & Wilkins, Philadelphia*.; 2007:2299–2354.

8. Mire CE, Miller AD, Carville A, et al. Recombinant vesicular stomatitis virus vaccine vectors expressing filovirus glycoproteins lack neurovirulence in nonhuman primates. *PLoS Negl Trop Dis*. 2012;6. doi:10.1371/journal.pntd.0001567.

## 14. Annexes

**List of Annexes**

1. Annex 1: Study site description
   CERMEL
   The Medical Research Center (CERMEL) of Lambaréné was established in 1981. Until June 2012, it was named "Medical Research Unit of the Albert Schweitzer Hospital (HAS – Hôpital Albert Schweitzer)". The recent changes of name and legal status from research unit to center reflect the increased activity of our institution.
   There are three research areas within the CERMEL: Clinical studies including epidemiology and clinical trials, Molecular Epidemiology and Immunology. The scientific focus so far on malaria and parasites research is being expanded to bacteriology and virology. Under these scientific areas, the topics of interest are pathogen biology, pathophysiology, pathogen-host interactions, host immune responses, as well as clinical aspects, chemotherapy and prevention of infectious diseases. A social sciences research group is currently being established whose scientific focus is to understand the patterns and impacts of health sciences on society in sub-Saharan Africa.
   In addition, two satellite sites have been added recently: the Medical Research Center of Fougamou, located 90 km south of Lambaréné in the province of Ngounié and the Medical Research Center of Makouké, located 40 km north-east of Lambaréné, opening up new research possibilities.
   The research center is integrated in the routine services of the ASH and is offering diagnostic and treatment follow up services to both patients consulting directly from Lambaréné and patients referred by collaborating governmental hospitals. The ASH (approximately 200 beds and 250 staff headed by 8 physicians) and run by an international foundation in collaboration with the Gabonese Ministry of Health, comprises the departments of internal medicine, surgery, pediatrics, obstetrics, a dental clinic, and a community health service where mother and child vaccinations are administered.
   The CERMEL is entirely self-financed through research projects and external grants. Current and past funders include the European Union, the European and Developing Countries Clinical Trials Partnership, PATH-MVI, the Medical Faculty of the University of Tübingen, the German Ministry of Education and Research, the US National Institute of Health, WHO/TDR, and pharmaceutical companies. More details on the Research Unit can be found at http://www.mru-lambarene.org and www.lambarene.org.

Clinical trials:

The site has extensive experience in the conduct of clinical trials, in accordance with ICH-GCP guidelines and the Declaration of Helsinki, including phase I to phase III trials on chemotherapy, prevention and candidate vaccines. The more recent clinical trials include the RTS,S malaria candidate vaccine, for which a total of three trials (two phase II trials and one phase III respectively) were conducted in Lambaréné during the past 7 years, with more than 1200 children (aged from 6 weeks to 59 months old) included and followed. Another malaria vaccine candidate GMZ2 was also evaluated in Lambaréné from phase Ib to phase II with more than 530 children (aged from 1-5 years old). On a smaller scale, we had conducted two trials on Influenza vaccine with the purposes to elicit the interactions between vaccine-induced responses and helminth infections.
Over 25 clinical trials have either been completed or are underway at the CERMEL (reference: http://clinicaltrials.gov/ct2/results?term=lambarene&pg=2). These achievements are reflected in our scientific output with an annual publications record of approximately 50 publications in international high impact peer-reviewed journals.

Brief description of study site infrastructure (e.g., vehicles, power, buildings/clinics, communications, facilities for cold storage, ability to send biological specimens overseas, ability to import vaccine from India, experience working with ICH/GCP trials and CROs, data management capabilities, record storage, demographic health surveillance system)
The CERMEL has two building facilities for clinical activities. The smallest is dedicated for early phase trials including Phase I/II studies. There are distinct areas in the phase I/II facility: an intervention area where vaccine administration will take place. In the post vaccine surveillance area, there 3 beds, life support equipment and drugs available for any major safety issue. In addition, CERMEL works closely with the two tertiary hospitals where hospitalization in mid or long term will take place. The main laboratory building hosts the department of hematology/biochemistry (called clinical laboratory) and the immunology and the molecular biology sub-units (together called research laboratory). The research laboratory hosts two more sub-units: the tuberculosis laboratory and the helminthes research laboratory (or parasitological laboratory). Staff ‘offices, meeting and conference rooms as well as the basement and the cold chain management area are located in the administrative building facility. CERMEL has an automobile park comprising of 5 cars including one bus. There is well functioning mobile phone coverage in Gabon, for national and international calls and communication.

There are IATA certified staff available to handle technical and logistic issues related to shipment and importation of vaccines. Import license for research goods has to be obtained at the drug office of the Ministry of Health in Libreville. IT, data entry and data analysis services are supported by VSAT internet connection via satellite, enabling efficient real time data entry and statistical support for clinical trials.

Health care services currently available to provide care for children with influenza and other respiratory illnesses

The health care infrastructure consists of two main hospitals, the Albert Schweitzer Hospital and the Lambaréné Regional Hospital (HRL). The HRL is situated near the airport of Lambaréné with a driving distance of about 15 km between HAS and HRL. In both hospitals, there are a pediatric ward, surgery, internal medicine, antenatal clinics and emergency services which can admit patients 24 hours per day as well as X-ray departments. In addition to these two hospitals, there are two primary health centers (Makouké and Fougamou) and about 10 functioning dispensaries in the villages and along the river Ogooué. The primary health centers and the dispensaries provide treatment for fever management.

Laboratory availability and experience (i.e., types of assays and indications)

There are 5 laboratory units in the CERMEL. One, the clinical laboratory runs the routine exams including (malaria diagnosis, hematology, biochemistry and rapid diagnosis test for several infectious diseases). The immunology laboratory and the molecular biology laboratories are equipped with BSL2 facilities for cell and parasite (P. falciparum) culture (flow hoods, CO2 and CO2/N2 incubators), as well as specialized instruments for molecular and cellular work, among which two flow cytometers: a bench top FacsCaliburTM (Becton Dickinson, 2 lasers/4 colours, multiparameter system) and a portable CyFlow TM (Partec, 3 colours, multiparameter system), cytokine analysis instruments (ELISA and ELISPOT readers, a Bioplex 200TM system), conventional and quantitative PCR cyclers, gels and gel photo documenting systems. The molecular biology laboratory also has nucleic acids amplification facilities with segregated pre- and post-PCR rooms. Sample storage at 4°C, -20°C, -80°C and -150°C is independent from the public electricity network via a high power electricity generator. The site is additionally equipped with its own liquid nitrogen production plant.

The microbiology laboratory performs in handling the pathogens culture and identification as well as antibiotic resistance. The Tuberculosis has the same scope as the microbiology laboratory but restricted to tuberculosis as requested by the BSL3 facilities. Finally, the helminthes research laboratory is specialized in providing accurate data on helminthes species, burden.

Description of ethics review committees and regulatory review agencies affiliated with your institution, including review timelines and their ability to review protocols according to international standards
The CERMEL has an Institutional Review Board. There is a National Ethics Committee in Gabon. Vaccine trials are submitted for national Ethics Committee approval. Upon ethical clearance, regulatory process is initiated applying for authorization through the "Direction Générale de la Santé" of the Gabonese Ministry of Health(MoH). In parallel, an authorization must be obtained from the Drug Office of the MoH to import research products. A time period of 6 up to 12 weeks is needed for ethical and regulatory clearance. The ethical review follows ICH-GCP and the Declaration of Helsinki.
Contact information and meeting frequency for ethics and national clinical trial approval committees.
•Gabon National Ethics Committee. Head: Dr Pierre Blaise Matsiegui (e-mail: pierre.blaise.matsiegui@gmail.com; contact: +241 07791200)
•MRU Institutional Ethics Committee. Head: Dr Ghyslain Mombo Ngoma (e-mail: ghyslain.mombongoma@gmail.com; contact: +241 07508437)
•MRU Scientific Review Board. Head: Prof Michael Ramharter (e-mail: michael.ramharter@meduniwien.ac.at, contact: +43 1404004485)

Annexe 2: Safety assessment, grading rules, Causality assessment, Reporting workflow

## SAFETY ASSESSMENT, GRADING RULES, CAUSALITY ASSESSMENT, REPORTING WORKFLOW

Safety will be assessed by the frequency, incidence, severity and nature of adverse events and serious adverse events arising during the study. A formal interim safety analysis will take place and may be repeated when approximately 50 % of participants are enrolled, subject to the advice of the DSMB as described in Section 4.4.

### Adverse events

An AE is any untoward medical occurrence in a subject that may occur during or after administration of an Investigational Medicinal Product (IMP) and does not necessarily have a causal relationship with the intervention. An AE can therefore be any unfavorable and unintended sign (including an abnormal laboratory finding), symptom or disease temporally associated with the study intervention, whether or not considered related to the study intervention.

Each AE will be graded according to the tables for AE severity below.The following guidelines will be used to determine whether or not an AE is recorded in the study database:

- Solicited AE (*i.e*, reactogenicity parameters) will be recorded on diary cards in the seven days and recorded in the CRF in the 14 days following injection
- Unsolicited AE of all severities will be recorded on diary cards in the seven days and recorded in the CRF in the 28 days following injection
- After Day 28 and throughout the remainder of the study period, only SAE will be recorded in the CRF

**Adverse reaction**

An adverse reaction (AR) is any untoward or unintended response to an IMP. This means that a causal relationship between the IMP and an AE is at least a reasonable possibility, i.e., the relationship cannot be ruled out. All cases judged by the reporting Investigator as having a reasonable suspected causal relationship to an IMP (i.e. possibly, probably or definitely related to an IMP) will qualify as AR.

**Serious adverse events**

A SAE is an AE that results in any of the following outcomes, whether or not considered related to the study intervention:

- Death
- Life-threatening event (i.e., the subject was, in the view of the Investigator, at immediate risk of death from the event that occurred). This does not include an AE that, if it occurred in a more severe form, might have caused death.
- Persistent or significant disability or incapacity (i.e., substantial disruption of one's ability to carry out normal life functions)
- Hospitalization, regardless of length of stay, even if it is a precautionary measure for continued observation. Hospitalization (including inpatient or outpatient hospitalization for an elective procedure) for a pre-existing condition that has not worsened unexpectedly does not constitute a SAE.

- An important medical event (that may not cause death, be life-threatening, or require hospitalization) that may, based upon appropriate medical judgment, jeopardize the subject and/or require medical or surgical intervention to prevent one of the outcomes listed above. Examples of such medical events include allergic reaction requiring intensive care in an emergency room or clinic, blood dyscrasias, or convulsions that do not result in inpatient hospitalization.
- Congenital anomaly or birth defect

**Serious adverse reaction (SAR)**

An AE (expected or unexpected) that is both serious and, in the opinion of the reporting investigator or Sponsors, believed to be possibly, probably or definitely due to an IMP or any other study treatments, based on the information provided.

**Suspected unexpected serious adverse reaction (SUSAR)**

A SUSAR is a SAE that is unexpected and thought to be possibly, probably or definitely related to an IMP. No category of SAE has been defined as 'expected.'

**Causality assessment**

For every unsolicited AE, an assessment of the relationship of the event to the administration of the vaccine will be undertaken. An intervention-related AE refers to an AE for which there is a probable or definite relationship to administration of a vaccine. An interpretation of the causal relationship of the intervention to the AE in question will be made, based on the type of event; the relationship of the event to the time of vaccine administration; and the known biology of the vaccine therapy.

Table 1: Guidelines for assessing the relationship of vaccine administration to an AE.

| Causality grading | | Explanation |
|---|---|---|
| 0 | **No Relationship** | No temporal relationship to study product ***and***<br><br>Alternate etiology (clinical state, environmental or other interventions); ***and***<br><br>Does not follow known pattern of response to study product |
| 1 | **Unlikely** | Unlikely temporal relationship to study product ***and***<br><br>Alternate etiology likely (clinical state, environmental or other interventions) ***and***<br><br>Does not follow known typical or plausible pattern of response to study product. |
| 2 | **Possible** | Reasonable temporal relationship to study product; ***or***<br><br>Event not readily produced by clinical state, environmental or other interventions; ***or***<br><br>Similar pattern of response to that seen with other vaccines |
| 3 | **Probable** | Reasonable temporal relationship to study product; ***and***<br><br>Event not readily produced by clinical state, environment, or other interventions ***or***<br><br>Known pattern of response seen with other vaccines |
| 4 | **Definite** | Reasonable temporal relationship to study product; ***and***<br><br>Event not readily produced by clinical state, environment, or other interventions; ***and***<br><br>Known pattern of response seen with other vaccines |

**Reporting procedures for all adverse events**

All AEs and SAEs occurring within the 28 and 184 days following injection and observed by the Investigator or reported by the volunteer, respectively, whether or not attributed to study intervention, will be recorded in the CRF. The investigator will assess every AE for seriousness and causality. A second assessment will be performed by a separate person (the Sponsor or delegate). All AEs that result in a volunteer's withdrawal from the study will be followed up until a satisfactory resolution occurs, or until a non-study related causality is assigned (if the volunteer consents to this).

**Reporting procedures for SAE**

Following the occurrence of any SAE, the trial subject's randomization code may be broken (see Section 6.4) and the PI shall report the event to the local safety monitor, the DSMB, the Sponsor, the vaccine manufacturer within 24 hours. In the case of a SAE considered possibly, probably or definitely related to the study medication, the PI will notify the other 3 PIs in the Consortium and WHO within 24 hours. For non-fatal SAE, the PI shall also report the event to the National EC and WHO EC, by means of the annual report. For SAE resulting in death, the PI shall report the event to the National EC as soon as possible but not later than 7 calendar days. WHO will report the event to its EC as soon as possible but not later than 7 calendar days.

**Reporting procedures for SUSAR**

Following the occurrence of a SUSAR, the trial subject's randomization code shall be broken (see Section 6.4) and the PI shall report the event to the local safety monitor, the DSMB, the Sponsor, the vaccine manufacturer, the other 3 PIs in the consortium and WHO within 24 hours. The PI shall also report the event to the National EC as soon as possible but not later than 7 calendar days for SUSAR resulting in death and 15 calendar days for non-fatal SUSAR. WHO will report the event to its EC as soon as possible but not later than 7 calendar days.

Of note, given that this first-in-human trial tests a GMO, the SAE will be assessed not only with regard to the GMO itself but also to the following aspects:

- Suspected viral or bacterial contamination from the GMO during initial preparation or later reconditioning
- A potential quality defect in the GMO or its components (medium, vector, etc.)
- A previously undetected disruption of the cold chain
- The administration (injection) of the GMO

**Assessment of severity**

The severity of clinical and laboratory AE will be assessed according to the scales in Tables 3 - 5.

Toxicity grading scale for local adverse events.

| **Toxicity grading scale for local adverse events** | | | | | |
|---|---|---|---|---|---|
| **Local Reaction** | **Grade 0**<br><br>**Normal** | **Grade 1**<br><br>**Mild** | **Grade 2**<br><br>**Moderate** | **Grade 3**<br><br>**Severe** | **Grade 4**<br><br>**Potentially life-threatening** |
| Redness/erythema* | <25 mm | 25-50 mm | 51-100 mm | >100 mm | Necrosis or exfoliative dermatitis |
| Swelling/induration** | <25 mm | 25-50 mm and does not interfere with activity | >50 mm or interferes with activity | prevents daily activity | Necrosis |
| Pain | None | Does not interfere with activity | Interferes with activity | Prevents daily activity | Emergency room visit or hospitalization |

*In addition to grading the measured local reaction at the greatest single diameter, the measurement should be recorded as a continuous variable.

**Induration/swelling should be evaluated and graded using the functional scale as well as the actual measurement.

From the FDA's 2007 voluntary guidance:
(http://www.fda.gov/downloads/BiologicsBloodVaccines/GuidanceComplianceRegulatoryInformation/Guidances/ Vaccines/ucm091977.pdf)

Table 4: Toxicity grading scale for physical observations.

| **Physical observations** | | | | | |
|---|---|---|---|---|---|
| **Observation** | **Grade 1** | **Grade 2** | **Grade 3** | **Grade 4** | **Grade 5** |
| Fever (axillary) | 38.0° - 39.0°C | > 39.0°C - 40.0°C | > 40.0°C for ≤ 24 hours | > 40.0°C for ≥ 24 hours | Death |
| Tachycardia (bpm)* | Asymptomatic, intervention not indicated | Symptomatic, non-urgent medical intervention indicated | Urgent medical intervention indicated | Life-threatening consequences, urgent intervention indicated | Death |
| Bradycardia (bpm)** | Asymptomatic, intervention not indicated | Symptomatic, medical intervention indicated | Severe, medically significant, urgent medical intervention indicated | Life-threatening consequences, urgent intervention indicated | Death |

| **Physical observations** | | | | | |
|---|---|---|---|---|---|
| **Observation** | **Grade 1** | **Grade 2** | **Grade 3** | **Grade 4** | **Grade 5** |
| Hypertension** | Prehypertension (systolic BP 120 - 139 mm Hg or diastolic BP 80 - 89 mm Hg) | Stage 1 hypertension (systolic BP 140 - 159 mm Hg or diastolic BP 90 - 99 mm Hg); medical intervention indicated; recurrent or persistent (>=24 h); symptomatic increase by >20 mm Hg (diastolic) or to >140/90 mm Hg if previously WNL; monotherapy indicated | Stage 2 hypertension (systolic BP >=160 mm Hg or diastolic BP >=100 mm Hg); medical intervention indicated; more than one drug or more intensive therapy than previously used indicated | Life-threatening consequences (e.g., malignant hypertension, transient or permanent neurologic deficit, hypertensive crisis); urgent intervention indicated | Death |
| Hypotension | Asymptomatic, intervention not indicated | Non-urgent medical intervention indicated | Medical intervention or hospitalization indicated | Life-threatening and urgent intervention indicated | Death |

| **Definitions** | |
|---|---|
| Fever | A disorder characterized by elevation of the body's temperature above the upper limit of normal. |
| Tachycardia | A disorder characterized by a dysrhythmia with a heart rate greater than 100 beats per minute. |
| Bradycardia | A disorder characterized by a dysrhythmia with a heart rate less than 60 beats per minute. |
| Hypertension | A disorder characterized by a pathological increase in blood pressure; a repeatedly elevation in the blood pressure exceeding 140 over 90 mm Hg. |
| Hypotension | A disorder characterized by a blood pressure that is below the normal expected for an individual in a given environment. |

*Taken after ≥10 minutes at rest; **when resting heart rate is between 60 – 100 beats per minute. Use clinical judgment when characterizing bradycardia among some healthy subjects, for example, conditioned athletes.

From CTCAE v4 (http://evs.nci.nih.gov/ftp1/CTCAE/CTCAE_4.03_2010-06-14_QuickReference_5x7.pdf)

Toxicity grading scale for systemic AEs excluding the physical observations listed above.

| Toxicity grading scale for systemic adverse events | | | | |
|---|---|---|---|---|
| **Systemic sign/symptom** | **Grade 1**<br><br>**Mild** | **Grade 2**<br><br>**Moderate** | **Grade 3**<br><br>**Severe** | **Grade 4**<br><br>**Potentially life-threatening** |
| Headache | No interference with activity | Some interference with activity | Significant; prevents daily activity | Medical consultation and/or hospitalization |
| Fatigue | No interference with activity | Some interference with activity | Significant; prevents daily activity | Medical consultation or hospitalization |
| Myalgia | No interference with activity | Some interference with activity | Significant; prevents daily activity | Medical consultation and/or hospitalization |
| Arthralgia | No interference with activity | Some interference with activity | Significant; prevents daily activity | Medical consultation and/or hospitalization |
| Chills | No interference with activity | Some interference with activity | Significant; prevents daily activity | Medical consultation and/or hospitalization |
| Sweats | No interference with activity | Some interference with activity | Significant; prevents daily activity | Medical consultation and/or hospitalization |

| | | | | |
|---|---|---|---|---|
| Subjective Fever | No interference with activity | Some interference with activity | Significant; prevents daily activity | Medical consultation and/or hospitalization |
| Nausea | No interference with activity | Some interference with activity not requiring medical intervention | Prevents daily activity and requires medical intervention | Medical consultation and/or hospitalization |
| Vomiting | No interference with activity | Some interference with activity not requiring medical intervention | Prevents daily activity and requires medical intervention | Medical consultation and/or hospitalization |
| Abdominal Pain | No interference with activity | Some interference with activity not requiring medical intervention | Prevents daily activity and requires medical intervention | Medical consultation and/or hospitalization |
| Diarrhea | No interference with activity | Some interference with activity not requiring medical intervention | Prevents daily activity and requires medical intervention | Medical consultation and/or hospitalization |
| Other systemic symptoms | No interference with activity | Some interference with activity | Prevents daily activity | Medical consultation and/or hospitalization |

From the FDA's 2007 voluntary guidance:
(http://www.fda.gov/downloads/BiologicsBloodVaccines/GuidanceComplianceRegulatoryInformation/Guidances/ Vaccines/ucm091977.pdf)
