# Supplementary material for: Safety and immunogenicity of rVSVΔG-ZEBOV-GP Ebola vaccine in adults and children in Lambaréné, Gabon: A phase I randomised trial
Source: PLoS Med. 2017 Oct 6;14(10):e1002402. doi: 10.1371/journal.pmed.1002402 (PMC5630143; doi:10.1371/journal.pmed.1002402)
Supplement: S2 Text — (DOC) [file pmed.1002402.s021.doc]

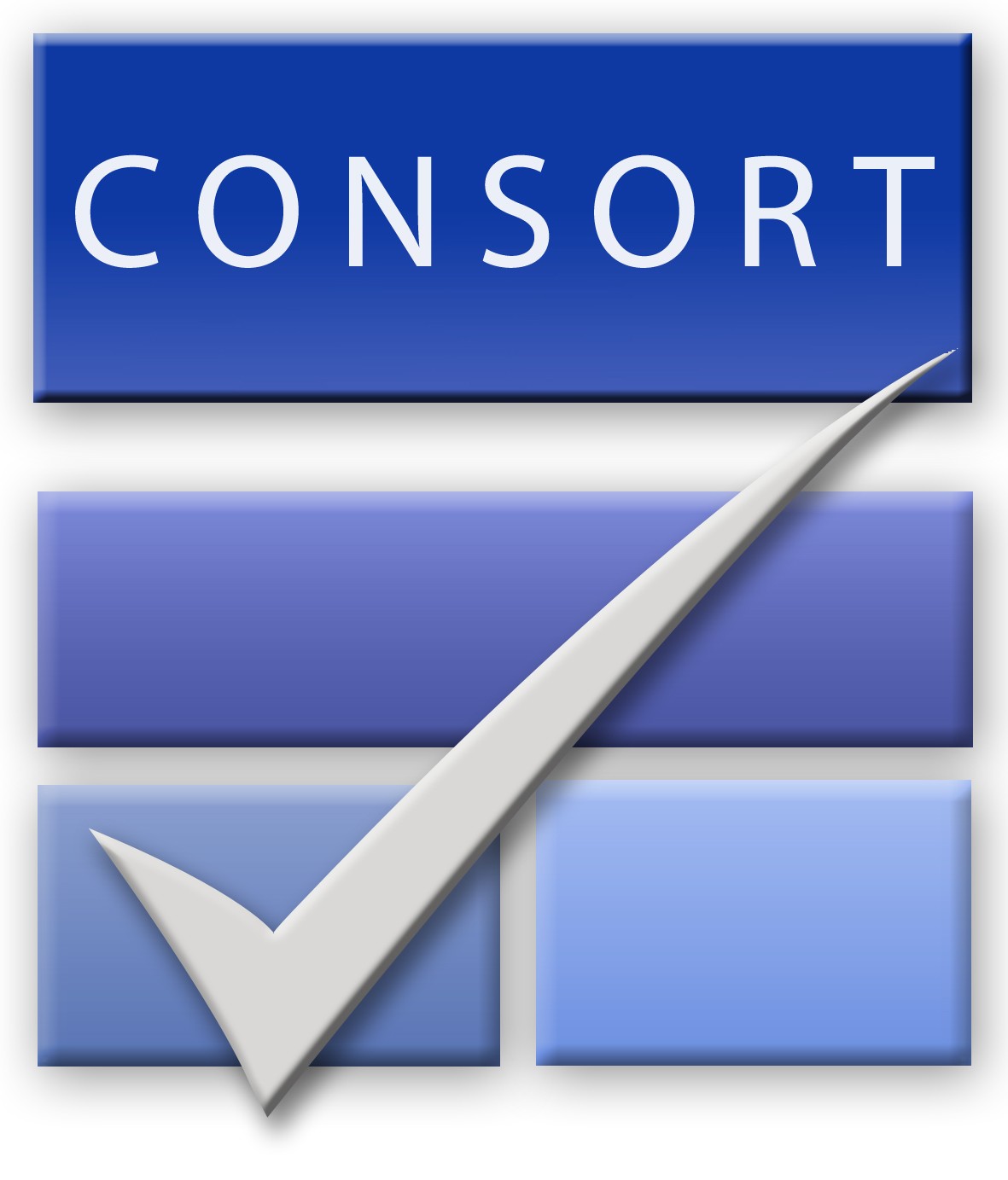
CONSORT 2010 checklist of information to include when reporting a randomised trial*

| Section/Topic | Item No | Checklist item | Reported on page No |
| --- | --- | --- | --- |
| Title and abstract | | | |
|  | 1a | Identification as a randomised trial in the title | Yes |
|  | 1b | Structured summary of trial design, methods, results, and conclusions (for specific guidance see CONSORT for abstracts) | Abstract |
| Introduction | | | |
| Background and objectives | 2a | Scientific background and explanation of rationale | Introduction paragraph 1-4 |
|  | 2b | Specific objectives or hypotheses | Introduction paragraph 4/methods paragraph 2 |
| Methods | | | |
| Trial design | 3a | Description of trial design (such as parallel, factorial) including allocation ratio | Methods/study design and participant paragraph 2 and 3 |
|  | 3b | Important changes to methods after trial commencement (such as eligibility criteria), with reasons | Methods /participants paragraph 2-3 and S2 Text |
| Participants | 4a | Eligibility criteria for participants | Method/Study design paragraph 2 and 3 |
|  | 4b | Settings and locations where the data were collected | Method paragraph 2 |
| Interventions | 5 | The interventions for each group with sufficient details to allow replication, including how and when they were actually administered | Methods/ Randomisation / treatment allocation paragraph 4-5 |
| Outcomes | 6a | Completely defined pre-specified primary and secondary outcome measures, including how and when they were assessed | Method/ Outcomes paragraph 9-10 |
|  | 6b | Any changes to trial outcomes after the trial commenced, with reasons | No |
| Sample size | 7a | How sample size was determined | Methods/Statistics paragraph 11 |
|  | 7b | When applicable, explanation of any interim analyses and stopping guidelines | Yes-Method/Randomization/treatment allocation paragraph 4-5 |
| Randomisation: |  |  |  |
| Sequence generation | 8a | Method used to generate the random allocation sequence | Yes-Method/Randomization/treatment allocation paragraph 4-5, S1 Text . |
|  | 8b | Type of randomisation; details of any restriction (such as blocking and block size) | Yes-Method/Randomization/treatment allocation paragraph 4-5, S1 Text . |
| Allocation concealment mechanism | 9 | Mechanism used to implement the random allocation sequence (such as sequentially numbered containers), describing any steps taken to conceal the sequence until interventions were assigned | Yes-Method/Randomization/treatment allocation paragraph 4-5, S1 Text . |
| Implementation | 10 | Who generated the random allocation sequence, who enrolled participants, and who assigned participants to interventions | Yes-Method/Randomization/treatment allocation paragraph 4-5, S1 Text . |
| Blinding | 11a | If done, who was blinded after assignment to interventions (for example, participants, care providers, those assessing outcomes) and how | Investigators were concealed to randomisation a list |
|  | 11b | If relevant, description of the similarity of interventions | NA |
| Statistical methods | 12a | Statistical methods used to compare groups for primary and secondary outcomes | Methods/Statistics paragraph 11 |
|  | 12b | Methods for additional analyses, such as subgroup analyses and adjusted analyses | No |
| Results | | | |
| Participant flow (a diagram is strongly recommended) | 13a | For each group, the numbers of participants who were randomly assigned, received intended treatment, and were analysed for the primary outcome | Yes -Fig 1 |
|  | 13b | For each group, losses and exclusions after randomisation, together with reasons | Yes-Fig 1 and Results paragraph 6 |
| Recruitment | 14a | Dates defining the periods of recruitment and follow-up | Yes-Results paragraph 1 |
|  | 14b | Why the trial ended or was stopped | NA |
| Baseline data | 15 | A table showing baseline demographic and clinical characteristics for each group | Yes- Results Table 1, |
| Numbers analysed | 16 | For each group, number of participants (denominator) included in each analysis and whether the analysis was by original assigned groups | Yes, Tables |
| Outcomes and estimation | 17a | For each primary and secondary outcome, results for each group, and the estimated effect size and its precision (such as 95% confidence interval) | Yes, Tables |
|  | 17b | For binary outcomes, presentation of both absolute and relative effect sizes is recommended | Yes |
| Ancillary analyses | 18 | Results of any other analyses performed, including subgroup analyses and adjusted analyses, distinguishing pre-specified from exploratory | NA |
| Harms | 19 | All important harms or unintended effects in each group (for specific guidance see CONSORT for harms) | Yes-Results paragraph 6 |
| Discussion | | | |
| Limitations | 20 | Trial limitations, addressing sources of potential bias, imprecision, and, if relevant, multiplicity of analyses | Yes-Discussion paragraph 11 |
| Generalisability | 21 | Generalisability (external validity, applicability) of the trial findings | Yes-Discussion paragraph 12 |
| Interpretation | 22 | Interpretation consistent with results, balancing benefits and harms, and considering other relevant evidence | Yes-discussion paragraph 1-12 |
| Other information | | |  |
| Registration | 23 | Registration number and name of trial registry | Yes-Method paragraph 1 |
| Protocol | 24 | Where the full trial protocol can be accessed, if available | Submitted with this manuscript |
| Funding | 25 | Sources of funding and other support (such as supply of drugs), role of funders | Yes-Funding |

*We strongly recommend reading this statement in conjunction with the CONSORT 2010 Explanation and Elaboration for important clarifications on all the items. If relevant, we also recommend reading CONSORT extensions for cluster randomised trials, non-inferiority and equivalence trials, non-pharmacological treatments, herbal interventions, and pragmatic trials. Additional extensions are forthcoming: for those and for up to date references relevant to this checklist, see [www.consort-statement.org](http://www.consort-statement.org/).
