# Supplementary material for: Safety and immunogenicity of rVSVΔG-ZEBOV-GP Ebola vaccine in adults and children in Lambaréné, Gabon: A phase I randomised trial
Source: PLoS Med. 2017 Oct 6;14(10):e1002402. doi: 10.1371/journal.pmed.1002402 (PMC5630143; doi:10.1371/journal.pmed.1002402)
Supplement: S3 Text — (DOCX) [file pmed.1002402.s022.docx]

# S3 Text. Dose escalation and Randomization

Participants were vaccinated into seven cohorts with five vaccine doses. Dose escalation was performed for cohort one-3x10^5^PFU, cohort two-3x10^6^PFU, and cohort three-2x10^7^PFU as follows:

The trial was initially designed to escalate doses of 3x10^5^, 3x10^6^ and 2x10^7^ PFU in 60 adults; it comprised of three randomised cohorts of 20 participants each. Vaccination in each cohort proceeded in a staggered fashion. On day one, the first volunteer of cohort one was vaccinated, followed by the 2nd and 3rd volunteer on Day three- Five participants were vaccinated on day five, as no safety alerts were reported in the first three vaccinations. As no safety concern was reported, the next five-ten participants were vaccinated. The DSMB reviewed the safety data captured up to the fifth visit for the first five to ten vaccinees in cohort one. Vaccinations into cohorts two, and three were performed after clearance from the DSMB. Participants were enrolled into cohorts two and three in the same manner as cohort one.

Randomization and allocation was performed by an investigator independent of the recruiting and clinical team. Randomisation lists were generated at www.randomization.com using randomly permuted blocks of varying sizes prior to study launch or prior to the start of screening for cohort four and five.

From November 17, 2014 until January 9, 2015, a randomization list was generated to assign 60 adults to the doses of either 3x10^5^, 3x10^6^ and 2x10^7^ PFU in a ratio of 1:1:1 respectively. During the trial, a temporary consortium-wide safety based hold (on December 9,2014) was placed on all doses due to adverse events reported at the Swiss study site with vaccine doses of 1x10^7^ and 5x10^7^ PFU. In Gabon, vaccination with 2x10^7^ PFU was discontinued, due to a sponsor request to hold vaccinations above 1x10^7^ PFU.

On 7^th^ March 2015, ethical approval was obtained for a protocol amendment to evaluate the two lower doses of 3x10^3^ PFU or 3x10^4^ PFU. A second randomization list was generated using random permuted block of varying sizes to assign 40 adult participants to either 3x10^4^ PFU or 3x10^3^ PFU, respectively in a 1:1 ratio.

A subsequent amendment included 20 adolescents and 20 children aged 13 to 17 years and 6 to 12 years, respectively, to be vaccinated with 2x10^7^ PFU. The National Ethics Committee of Gabon recommended that adults from this population should be vaccinated with the intended dose before administration to the paediatric cohorts, so an additional 15 adults were included in the study. Vaccinations of cohort seven (6-12 years) was only performed after the first ten adolescents (13-17 years) had completed day 28 and after all adults’ vaccinees had completed day 28.
