# Supplementary material for: Safety and immunogenicity of rVSVΔG-ZEBOV-GP Ebola vaccine in adults and children in Lambaréné, Gabon: A phase I randomised trial
Source: PLoS Med. 2017 Oct 6;14(10):e1002402. doi: 10.1371/journal.pmed.1002402 (PMC5630143; doi:10.1371/journal.pmed.1002402)
Supplement: S4 Text — (DOCX) [file pmed.1002402.s023.docx]

# S4 Text. Data collection, management and Safety Assessment

Data were captured into electronic case report forms launched at secuTrial® web-based clinical data management system; <https://secutrial.hcuge.ch/>. External Clinical Research Associates (CRA) reviewed 100% medical records, samples storage, and laboratory shipment records to ensure data completeness and integrity.

Investigators were responsible for documenting and reporting all events meeting the criteria of an adverse event (AE) or serious adverse event (SAE). The participants, parent or guardian were requested to contact the trial staff immediately if they or their children had any medical condition.

An adverse event (AE) was defined as any untoward medical occurrence in adults and children participating in the study associated with vaccination, whether it was considered related to the vaccine or not. Local injection site reactions such as swelling, pain, and erythema were classified as solicited local AE recorded within 14 days post vaccination.

Any untoward systemic medical condition reported within 14 days post vaccination, classified in the protocol as related to vaccine were considered as solicited systemic adverse events. The protocol was designed to assess fever as Objective or subjective.

Objective fever was defined as fever with axillary temperature greater than or equal to 38∙0°C. Subjective fever was considered as a feeling of fever described or reported by the participant or his/her parent or guardian not supported by an axillary temperature equal to or greater than 38∙0°C.

The occurrence of any other untoward medical condition not classified in the protocol as solicited, occurring post vaccination until day 28 were considered as unsolicited systemic adverse events.

In this trial, a SAE was defined as any untoward medical occurrence that resulted in death, was life threatening, required hospitalization or prolong hospitalization of existing hospitalization, resulted in disability/incapacity post vaccination and/or any condition considered by the investigator to be medically important. Abnormal laboratory findings, that were judge by the investigator to be clinically significant were reported as AEs, if they met the SAE criteria defined above, they were reported as SAE.

All AEs that were observed directly or reported by parents/guardians spontaneously or in response to a direct question were evaluated and reported. Assessments were made of the maximum intensity of all unsolicited AEs and SAEs during the period of the event. This assessment was based on the attending clinician’s medical judgment. A grade was assigned to all adverse events as follows; grade 1 (mild) - an AE which was easily tolerated by the participant, causing minimal discomfort and not interfering with everyday activities; grade 2 (moderate) - an AE which was sufficiently discomforting to interfere with normal everyday activities and grade 3 (severe) - an AE which prevented normal, everyday activities. All AEs were coded according to the MedDRA (Medical Dictionary for Drug Regulatory Activities).

Frequency and intensity of adverse events were described as counts and percentages. Only adverse events of the highest grade were reported for analysis. Chi-squared test was used to compare the distribution of events within cohorts. Fisher’s exact test was used- when expected counts within a cohort was less than five. We explored the distribution of adverse events with regards to baseline status of ZEBOV-GP-specific antibodies in count and percentages using Fisher’s exact test.

Hematology and biochemistry parameters were expressed in means and ranges. Kruskal-wallis test was used to perform inter-cohort comparisons at different study timepoints screening, day one (D1), day two (D2), day seven(D7), day 28 (D28), and day180 (D180).
